# Comparative survival analysis of multiparametric tests—when molecular tests disagree—A TEAM Pathology study

**DOI:** 10.1038/s41523-021-00297-7

**Published:** 2021-07-08

**Authors:** John M. S. Bartlett, Jane Bayani, Elizabeth Kornaga, Keying Xu, Greg R. Pond, Tammy Piper, Elizabeth Mallon, Cindy Q. Yao, Paul C. Boutros, Annette Hasenburg, J. A. Dunn, Christos Markopoulos, Luc Dirix, Caroline Seynaeve, Cornelis J. H. van de Velde, Robert C. Stein, Daniel Rea

**Affiliations:** 1 Diagnostic Development, Ontario Institute for Cancer Research, Toronto, ON Canada; 2 Laboratory Medicine and Pathobiology, University of Toronto, Toronto, ON Canada; 3 Edinburgh Cancer Research Centre, Edinburgh, UK; 4 Translational Laboratories, Tom Baker Cancer Centre, Calgary, AB Canada; 5 Department of Oncology, McMaster University, Kingston, ON Canada; 6 Department of Pathology, Glasgow, UK; 7 Informatics & Computational Biology, Ontario Institute for Cancer Research, Toronto, ON Canada; 8 Department of Medical Biophysics, University of Toronto, Toronto, Canada; 9 Department of Pharmacology & Toxicology, University of Toronto, Toronto, Canada; 10 Jonsson Comprehensive Cancer Center, University of California, Los Angeles, USA; 11 Dept of Gynecology and Obstetrics, University Center Mainz, Mainz, Germany; 12 University of Warwick, Coventry, UK; 13 National and Kapodistrian University of Athens, Medical School, Athens, Greece; 14 St. Augustinus Hospital, Antwerp, Belgium; 15 Erasmus MC Cancer Institute, Rotterdam, the Netherlands; 16 Leiden University Medical Center, Leiden, the Netherlands; 17 National Institute for Health Research University College London Hospitals Biomedical Research Centre, London, UK; 18 Cancer Research UK Clinical Trials Unit, University of Birmingham, Birmingham, UK

**Keywords:** Prognostic markers, Breast cancer

## Abstract

Multiparametric assays for risk stratification are widely used in the management of both node negative and node positive hormone receptor positive invasive breast cancer. Recent data from multiple sources suggests that different tests may provide different risk estimates at the individual patient level. The TEAM pathology study consists of 3284 postmenopausal ER+ve breast cancers treated with endocrine therapy Using genes comprising the following multi-parametric tests OncotypeDx^®^, Prosigna™ and MammaPrint^®^ signatures were trained to recapitulate true assay results. Patients were then classified into risk groups and survival assessed. Whilst likelihood *χ*^2^ ratios suggested limited value for combining tests, Kaplan–Meier and LogRank tests within risk groups suggested combinations of tests provided statistically significant stratification of potential clinical value. Paradoxically whilst Prosigna-trained results stratified Oncotype-trained subgroups across low and intermediate risk categories, only intermediate risk Prosigna-trained cases were further stratified by Oncotype-trained results. Both Oncotype-trained and Prosigna-trained results further stratified MammaPrint-trained low risk cases, and MammaPrint-trained results also stratified Oncotype-trained low and intermediate risk groups but not Prosigna-trained results. Comparisons between existing multiparametric tests are challenging, and evidence on discordance between tests in risk stratification presents further dilemmas. Detailed analysis of the TEAM pathology study suggests a complex inter-relationship between test results in the same patient cohorts which requires careful evaluation regarding test utility. Further prognostic improvement appears both desirable and achievable.

## Introduction

Multi-parametric molecular tests are central to the treatment management of early breast cancer and their use is incorporated into most major guidelines^1^ as a pre-requisite for the staging of breast cancer patients, to direct prognostication and to select patients for chemotherapy treatment^2,3^. Two major challenges related to their use need to be addressed. Firstly, reports highlighting disagreements between tests are disquieting for physicians, health care providers, and patients alike^4^ since they raise the question “*have I recommended/received the right test*?” Secondly, the lack of consistency at an individual patient level between different tests suggests additional prognostic information may result from novel tests. Recent results from the MINDACT and TAILORx studies validate the utility of tests to direct chemotherapy use in node-negative patients^2,5,6^, which may be extended as new evidence emerges from retrospective^3^ or prospective studies^7,8^. In this context an error in assigning appropriate risk classifications would have significant impact on patient treatment and outcomes. Additionally, given recent evidence documenting the long-term risk of relapse for ER+ve breast cancer and the increasing use of extended endocrine therapy^9^ the selection of the appropriate test to detect recurrence risk over extended time periods is also critical.

Reports of disagreements between tests, based on in silico analyses of existing expression array data, were frequently attributed to methodological challenges and incomplete gene coverage^10–14^. However, recently direct comparisons, where tests were performed exactly to vendor protocols, demonstrate marked disagreement in risk categorization and subtyping of individual tumors between widely used multiparameter assays^4^. Furthermore, comparisons between tests in clinical trials derived cohorts provide consistent evidence that combining test results generally improves prognostic value^15,16^. These results may reflect the relatively modest performance of individual multiparametric tests^17^.

To date, no direct comparison between different multiparameter assays in a large patient cohort with associated follow-up provides robust information on the impact of discrepant test results for patients. We developed a method to compare signatures using a combined quantitative mRNA array covering key molecular signatures^17^, trained against the results of the same signatures measured by original methodology^18^. We analyzed >3000 samples from the TEAM pathology cohort^19^ using “trained” signatures to demonstrate the impact of disagreements between tests on patient outcome in the context of a recent clinical trial cohort.

## Results

### Comparing signature-trained risk scores—Likelihood ratios

**Fig. 1:**
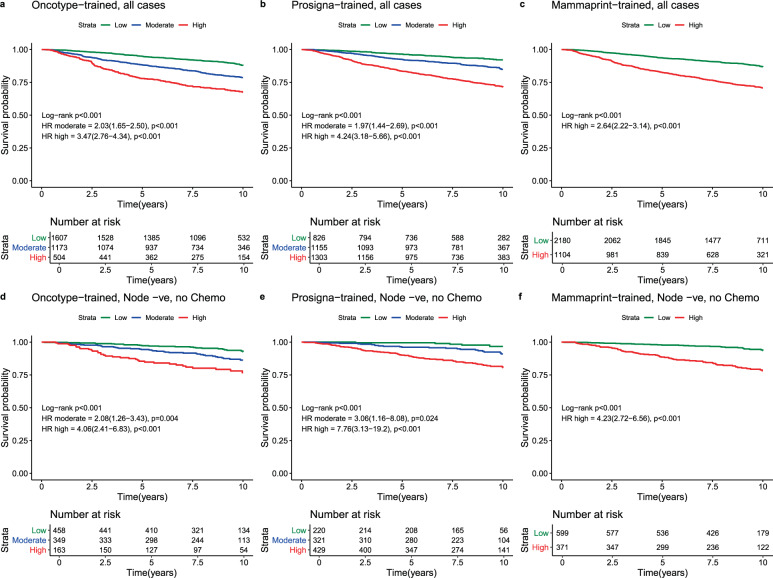
Test performance in ER+ve, HER2-ve breast cancer from the TEAM cohort. Kaplan–Meier survival curves with Log-rank Hazard ratios for cases of ER+ve, HER2−ve breast cancer from the entire TEAM cohort for Oncotype-trained (Panel **a**), Prosigna-trained (Panel **b**), and Mammaprint-trained results (Panel **c**) and for ER+ve, HER2−ve Node negative breast cancers treated without chemotherapy from the TEAM cohort for Oncotype-trained (Panel **d**), Prosigna-trained (Panel **e**), and Mammaprint-trained results (Panel 5). Log-Rank *P* values for each test are in brackets. Within each panel low (green), moderate (blue) and high (red) risk survival curves are plotted with LogRank Hazard ratios for high risk and intermediate risk (Oncotype-trained and Prosigna-trained only) calculated against low risk cases in each sub-group. 95% Confidence intervals for LogRank Hazard ratios are in brackets. For each group the number at risk (Low, moderate, high) are presented under the *X* axis.

We compared the ability of trained signatures to predict DMFS10 using the likelihood ratio *χ*^2^(LR*χ*^2^) based on the Cox models as a measure of the overall prognostic information provided by each model. We illustrated the performance of each “trained” test using Kaplan–Meier survival curves and estimated Hazard ratios as described above (see Fig. 1). We calculated the change in LR*χ*^2^ values(ΔLR*χ*^2^) between the reclassified and single signature models to assess prognostic improvement of reclassification with a second signature versus the single signature using existing trinary and binary (Table 1) cut points as outlined above.

**Table 1:** Likelihood *χ*^2^ ratios by test and cohort.

	ER+/HER2− (*N* = 3284)					
	Trinary classification			Binary classification		
	df	LR*χ*^2^	*p*-value	df	LR*χ*^2^	*p*-value
*Univariate models*						
Oncotype	2	118.0	<0.0001	1	109.87	<0.0001
Prosigna	2	146.9	<0.0001	1	127.31	<0.0001
Mammaprint	1	119.5	<0.0001	1	119.45	<0.0001
*Bivariate models*						
Oncotype + Prosigna	4	177.9	<0.0001	2	164.47	<0.0001
Oncotype + Mammaprint	3	145.7	<0.0001	2	143.34	<0.0001
Prosigna + Mammaprint	3	168.8	<0.0001	2	155.11	<0.0001
*Bivariate vs. univariate*						
Oncotype + Prosigna vs. Oncotype	2	59.97	<0.0001	1	54.60	<0.0001
Oncotype + Mammaprint vs. Oncotype	1	27.78	<0.0001	1	33.48	<0.0001
Prosigna + Oncotype vs. Prosigna	2	31.02	<0.0001	1	37.16	<0.0001
Prosigna + Mammaprint vs. Prosigna	1	21.89	<0.0001	1	27.80	<0.0001
MammaPrint + Oncotype vs. Mammaprint	2	26.28	<0.0001	1	23.89	<0.0001
Mammaprint + Prosigna vs. Mammaprint	2	49.34	<0.0001	1	35.65	<0.0001

LR*χ*^2^ = likelihood ratio chi-squared value, all models run exiting at 10 years. Likelihood *χ*^2^ ratios(LR*χ*^2^) for univariate(single test) or bivariate(two tests in sequence) derived using 10-year distant metastasis free survival as end point, ER+/HER2+ve cases = all ER+ve/HER2−ve cases (irrespective of nodal status and chemotherapy), ΔLR*χ*^2^ = change in Likelihood *χ*^2^ ratio when two tests are used sequentially. Trinary classification: results using results from Oncotype-Dx trained and Prosigna-trained tests categorized as low, intermediate, and high risk, binary classification: results using dichotomous results for all tests, see text for cut-points, ΔLR*χ*^2^ = change in LR*χ*^2^ for comparison of 2 tests versus a single test.

In ER+/HER2− cases (*n* = 3284), the Prosigna-trained signature provided greater prognostic information compared to Oncotype-trained and MammaPrint-trained signatures(LR*χ*^2^ = 146.9 vs. 118.0 and 119.5, respectively; Table 1). In bivariate models (combining 2 tests) the greatest LR*χ*^2^ was observed with Oncotype-trained and Prosigna-trained results (Table 1). Comparing bivariate and univariate results combining Oncotype-trained and Prosigna-trained results increased the LR*χ*^2^ to a far greater extent versus Oncotype-trained results (ΔLR*χ*^2^ = 60.0) than versus Prosigna-trained (ΔLR*χ*^2^ = 31.0) results. Similarly, when combining tests with Mammaprint-trained results adding Prosigna-trained results showed a greater increase in LR*χ*^2^ (ΔLR*χ*^2^ = 49.3) than did combining Mammaprint-trained results with Oncotype-trained results (ΔLR*χ*^2^ = 26.3). Adding Mammaprint-trained results to either Oncotype-trained or Prosigna-trained results to, versus either test produced the smallest improvements in the LR*χ*^2^ (Table 1). Nonetheless, all test combinations outperformed single tests to a highly statistically significant degree (*p* < 0.0001; Table 1).

When test results for Oncotype-trained and Prosigna-trained results were dichotomized, there were less marked differences in univariate models between these tests and Mammaprint-trianed results (Table 1). Again the largest increase in LR*χ*^2^ was observed when comparing combined Oncotype-trained and Prosigna-trained classification versus Oncotype-trained alone. All other bivariate models outperformed univariate models to a lesser, but still statistically significant, degree (*p* < 0.0001; Table 1).

### Analysis of test performance by outcome in reclassified patients

We analyzed agreement between tests by investigating the extent to which re-classifying results for individual patients by performing tests in sequence affected predicted outcome. Example, we estimated the effects of performing a Prosigna-trained test on tumors previously classified as intermediate risk by the Oncotype-trained test.

### Entire ER+ve/HER2−ve population

#### Oncotype-trained

**Fig. 2:**
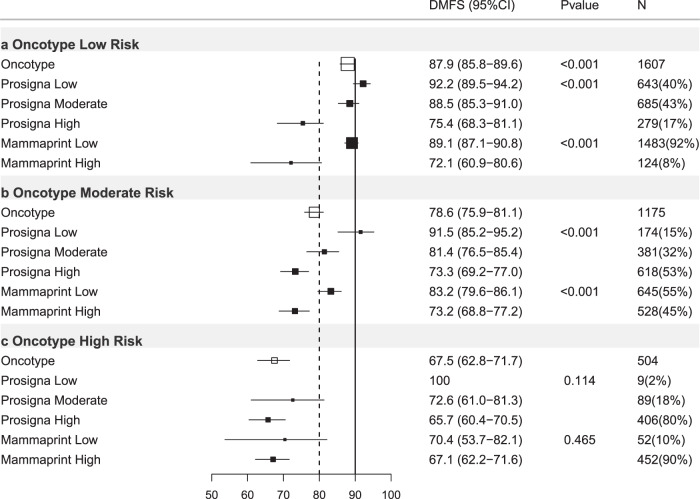
Forest plot of Oncotype-trained test results re-stratified by other tests, all ER+ve/HER2−ve cases. DMFS10 = distant metastasis free survival at 10 years post diagnosis. (95% CI) = 95% confidence interval, *P* *value* = *p* value, *N* = number of cases in each subgroup, % = percentage of cases within each risk strata. *X* axis = percent distant metastasis free survival. Open boxes represent primary test DMFS10 by risk group. Solid boxes represent sub-stratification by secondary tests with 95% confidence intervals (bars). Top panel (**a**) oncotype-trained low risk cases stratified by prosigna-trained and Mammaprint-trained results. Middle panel (**b**) oncotype-trained moderate risk group. Bottom panel (**c**) oncotype-trained high risk group.

Of 3284 ER+ve/HER2−ve breast cancers with results for the Oncotype-trained risk classification, 48.9% were classified low risk (DMFS10 = 87.9%), 35.8% intermediate risk (DMFS10 = 78.6%) and 15.3% high risk (DMFS10 = 67.5%) (Table 2; Figs. 1a, 2).

**Table 2:** Oncotype-trained results stratified by other test results, trinary classification.

Oncotype-trained first																											
	Oncotype-trained low risk									Oncotype-trained intermediate risk									Oncotype-trained high risk								
	HR (95% CI)			DMFS (95% CI)			P* (*N*)			HR (95% CI)			DMFS (95% CI)			*P* (*N*)			HR (95% CI)			DMFS (95% CI)			*P* (*N*)		
All cases	REF			87.9 (85.8–89.6)			<0.001 (1607)			2.03 (1.65–2.50)			78.6 (75.9–81.1)			<0.001 (1173)			3.47 (2.76–4.34)			67.5 (62.8–71.7)			<0.001 (504)		
N−Ch−	REF			92.5 (88.8–95.0)			<0.001 (458)			2.08 (1.26–3.43)			86.3 (81.6–89.9)			0.004 (349)			4.06 (2.41–6.83)			76.7 (68.4–83.0)			<0.001 (163)		
N+Ch−	REF			86.4 (83.1–89.1)			<0.001 (683)			2.03 (1.48–2.78)			77.0 (72.0–81.2)			<0.001 (403)			4.64 (3.32–6.47)			55.1 (46.4–63.0)			<0.001 (161)		
Ch+	REF			85.4 (81.2–88.8)			<0.001 (463)			2.03 (1.45–2.82)			73.8 (68.8–78.2)			<0.001 (418)			2.48 (1.68–3.65)			70.8 (63.0–77.2)			<0.001 (179)		
	**Prosigna-trained low**			**Prosigna trained Int**			**Prosigna trained high**			**Prosigna-trained low**			**Prosigna trained Int**			**Prosigna-trained high**			**Prosigna-trained low**			**Prosigna-trained Int**			**Prosigna-trained high**		
	**HR**	**DMFS**	P*	**HR**	**DMFS**	***P***	**HR**	**DMFS**	***P***	**HR**	**DMFS**	P*	**HR**	**DMFS**	***P***	**HR**	**DMFS**	***P***	**HR**	**DMFS**	P*	**HR**	**DMFS**	***P***	**HR**	**DMFS**	***P***
All cases	REF	92.2 (89.5–94.2)	<0.001 (643)	1.39 (0.94–2.06)	88.5 (85.3–91.0)	0.099 (685)	3.19 (2.12–4.82)	75.4 (68.3–81.1)	<0.001 (279)	REF	91.5 (85.2–95.2)	<0.001 (174)	2.41 (1.30–4.48)	81.4 (76.5–85.4)	0.005 (381)	3.70 (2.05–6.67)	73.3 (69.2–77.0)	<0.001 (618)	REF	100	0.114 (9)	NA	72.6 (61.0–81.3)	NA (89)	NA	65.7 (60.4–70.5)	NA (406)
N−Ch−	REF	97.1 (92.5–98.9)	0.011 (174)	2.52 (0.80–7.92)	92.4 (86.3–95.8)	0.114 (189)	5.08 (1.59–16.2)	83.8 (70.1–91.6)	0.006 (95)	REF	94.1 (65.0–99.1)	0.090 (43)	4.09 (0.52–31.9)	87.5 (77.3–93.4)	0.180 (106)	6.09 (0.83–44.8)	83.7 (77.1–88.5)	0.076 (200)	REF	100	0.058 (3)	NA	94.7 (68.1–99.2)	NA (26)	NA	72.8 (63.4–80.2)	NA (134)
N+Ch−	REF	89.3 (84.4–92.7)	<0.001 (263)	0.93 (0.53–1.62)	89.1 (84.1–92.5)	0.795 (309)	2.88 (1.63–5.07)	70.3 (58.1–79.6)	<0.001 (111)	REF	94.3 (83.4–98.1)	<0.001 (54)	2.81 (0.83–9.41)	81.8 (73.1–87.9)	0.094 (142)	5.68 (1.78–18.2)	69.0 (61.4–75.4)	0.003 (207)	REF	100	0.148 (1)	NA	74.5 (51.7–87.7)	NA (25)	NA	51.1 (41.5–59.8)	NA (135)
Ch+	REF	91.9 (86.5–95.2)	<0.001 (205)	1.98 (1.03–3.80)	83.4 (76.0–88.7)	0.041 (185)	3.76 (1.83–7.70)	71.7 (57.0–82.1)	<0.001 (73)	REF	87.9 (76.9–93.9)	0.007 (77)	2.26 (1.03–4.94)	76.2 (67.4–83.0)	0.041 (133)	3.03 (1.45–6.33)	67.4 (59.7–74.0)	0.003 (208)	REF	100	0.108 (5)	NA	56.4 (37.0–71.9)	NA (38)	NA	73.8 (65.1–80.7)	NA (136)
	**Mammaprint-trained low**						**Mammaprint trained high**			**Mammaprint-trained low**						**Mammaprint trained high**			**Mammaprint-trained low**						**Mammaprint trained high**		
	**HR**	**DMFS**	P*				**HR**	**DMFS**	***P***	**HR**	**DMFS**	P*				**HR**	**DMFS**	***P***	**HR**	**DMFS**	P*				**HR**	**DMFS**	***P***
All cases	REF	89.1 (87.1–90.8)	<0.001 (1483)				2.79 (1.83–4.25)	72.1 (60.9–80.6)	<0.001 (124)	REF	83.2 (79.6–86.1)	<0.001 (645)				1.70 (1.30–2.23)	73.2 (68.8–77.2)	<0.001 (528)	REF	70.4 (53.7–82.1)	0.465 (52)				1.24 (0.70–2.18)	67.1 (62.2–71.6)	0.466 (452)
N−Ch−	REF	93.8 (90.0–96.2)	0.002 (407)				3.57 (1.49–8.55)	80.8 (62.8–90.7)	0.004 (51)	REF	92.2 (85.6–95.8)	0.003 (174)				2.87 (1.40–5.88)	80.8 (73.4–86.4)	0.004 (175)	REF	100	0.034 (18)				NA	74.0 (65.0–81.0)	NA (145)
N+Ch−	REF	87.7 (84.4–90.3)	<0.001 (639)				2.94 (1.55–5.59)	67.6 (48.0–81.2)	0.001 (44)	REF	84.2 (78.2–88.7)	<0.001 (237)				2.30 (1.47–3.60)	66.9 (58.3–74.1)	<0.001 (166)	REF	54.2 (28.0–74.5)	0.945 (20)				1.03 (0.49–2.15)	55.3 (46.0–63.6)	0.945 (141)
Ch+	REF	86.8 (82.6–90.0)	0.002 (434)				3.03 (1.43–6.41)	64.2 (37.6–81.8)	0.004 (29)	REF	75.8 (69.1–81.2)	0.318 (233)				1.23 (0.82–1.83)	71.2 (63.0–77.9)	0.319 (185)	REF	61.1 (29.8–81.9)	0.537 (14)				0.75 (0.30–1.89)	71.9 (63.8–78.4)	0.538 (165)

HR = hazard ratio, 95% CI = 95% confidence interval, P* = *p* value of log-rank test to compare survival distributions, REF = reference group, *P* = *p* value of Wald test for comparison versus reference (low risk) group, DMFS = distant metastasis free survival at 10 years (see text), (*N*) = number of cases in subgroups, All cases = all ER+ve/HER2-ve cases, N−Ch− = Node negative cases treated without chemotherapy, N+Ch− = Node positive cases treated without chemotherapy, Ch+ = cases treated with chemotherapy (node negative and node positive combined), Int = intermediate, P*:*p*-value of log-rank test to compare survival distributions (global statistical significance of the model).*P*: *p*-value of Wald-test to evaluate whether the hazard ratio is 1 (statistical significance of each individual coefficient).

### Oncotype-trained stratified by Prosigna-trained

When Oncotype-trained results were further stratified by Prosigna-trained results a significant proportion (56.5%) of cases changed risk category (Supplementary Table 2). In Oncotype-trained low-risk cases, 279 (17.4%) were re-classified as high risk by Prosigna-trained results and 9 Oncotype-trained high-risk cases (1.8%) were re-classified as low risk by Prosigna-trained results. Oncotype-trained low risk/Prosigna-trained high-risk cases exhibited a significantly reduced DMFS10 (75.4%) relative to cases low risk by both signatures (HR = 3.19; 95%CI 2.12–4.82; *p* < 0.001; Table 2; Fig. 2). For Oncotype-trained intermediate-risk cases, 174 (14.8%) were classified as Prosigna-trained low risk with a DMFS10 = 91.5% (*p* < 0.001; Table 2; Fig. 2), and 618 (52.6%) were classified as Prosigna-trained high risk (DMFS10 = 73.3%; Table 2; Fig. 2). Few Oncotype-trained high-risk tumors were low risk by Prosigna-trained scores and no events were observed in these cases.

### Oncotype-trained stratified by MammaPrint-trained

124 Oncotype-trained low-risk cases (8%) were high risk by MammaPrint-trained (DMFS10 = 72.1%; Table 2; Fig. 2; *p* < 0.001). 52 Oncotype-trained high-risk cases (10%) were low risk by MammaPrint-trained (DMFS10 = 70.4%; Table 2; Fig. 2; *p* = 0.465). Finally 528 (45%) Oncotype-trained intermediate-risk cases were MammaPrint-trained high risk(DMFS10 = 73.2%; Table 2; Fig. 2; *p* < 0.001).

#### Prosigna-trained results

**Fig. 3:**
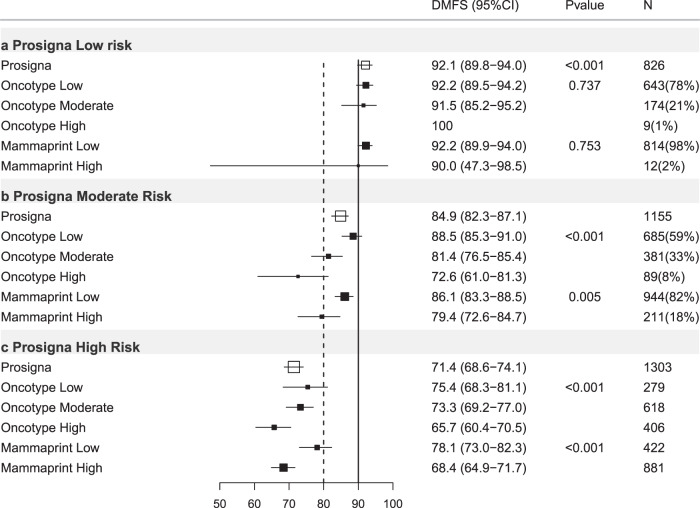
Forest plot of Prosigna-trained test results re-stratified by other tests, all ER+ve/HER2-ve cases. DMFS10 = distant metastasis free survival at 10 years post diagnosis, (95% CI) = 95% confidence interval, *P* = *p* value, *N* = number of cases in each subgroup, % = percentage of cases within each risk strata, *X* axis = percent distant metastasis free survival. Open boxes represent primary test DMFS10 by risk group. Solid boxes represent sub-stratification by secondary tests with 95% confidence intervals (bars). Top panel (**a**) prosigna-trained low risk cases stratified by Oncotype-trained and Mammaprint-trained results. Middle panel (**b**) prosigna-trained moderate-risk group. Bottom panel (**c**) prosigna-trained high risk group.

Of 3284 ER+ve/HER2−ve cases with results for Prosigna-trained risk available 25.2% were low risk (DMFS10 = 92.1%, 95%CI 89.8–94.0%), 35.2% intermediate risk (DMFS10 = 84.9%, 95%CI 82.3–87.1%) and 39.7% high risk (DMFS10 = 71.4%, 95%CI 68.6–74.1%; Table 3; Figs. 1b, 3).

**Table 3:** Prosigna-trained results stratified by other test results, trinary classification.

Prosigna-trained first																											
	Prosigna-trained low risk									Prosigna-trained intermediate risk									Prosigna-trained high risk								
	HR (95% CI)			DMFS (95% CI)			P* (*N*)			HR (95% CI)			DMFS (95% CI)			*P* (*N*)			HR (95% CI)			DMFS (95% CI)			*P* (*N*)		
All cases	REF			92.1 (89.8–94.0)			<0.001 (826)			1.97 (1.44–2.69)			84.9 (82.3–87.1)			<0.001 (1155)			4.24 (3.18–5.66)			71.4 (68.6–74.1)			<0.001 (1303)		
N−Ch−	REF			96.7 (92.0–98.7)			<0.001 (220)			3.06 (1.16–8.08)			91.0 (86.4–94.1)			0.024 (321)			7.76 (3.13–19.2)			80.5 (75.7–84.3)			<0.001 (429)		
N+Ch−	REF			90.1 (85.8–93.2)			<0.001 (318)			1.35 (0.85–2.15)			86.2 (82.1–89.4)			0.204 (476)			4.43 (2.93–6.69)			63.9 (58.6–68.7)			<0.001 (453)		
Ch+	REF			90.9 (86.3–94.0)			<0.001 (287)			2.64 (1.63–4.27)			77.8 (72.5–82.2)			<0.001 (356)			3.74 (2.37–5.92)			70.4 (65.2–74.9)			<0.001 (417)		
	**Oncotype-trained Low**			**Oncotype-trained Int**			**Oncotype-trained high**			**Oncotype-trained low**			**Oncotype-trained Int**			**Oncotype-trained high**			**Oncotype-trained low**			**Oncotype-trained Int**			**Oncotype-trained high**		
	**HR**	**DMFS**	P*	**HR**	**DMFS**	***P***	**HR**	**DMFS**	***P***	**HR**	**DMFS**	P*	**HR**	**DMFS**	***P***	**HR**	**DMFS**	***P***	**HR**	**DMFS**	P*	**HR**	**DMFS**	***P***	**HR**	**DMFS**	***P***
All cases	REF	92.2 (89.5–94.2)	0.737 (643)	1.07 (0.56–2.03)	91.5 (85.2–95.2)	0.835 (174)	NA	100	NA (9)	REF	88.5 (85.3–91.0)	<0.001 (685)	1.86 (1.31-2.66)	81.4 (76.5-85.4)	0.001 (381)	3.08 (1.89-5.01)	72.6 (61.0-81.3)	<0.001 (89)	REF	75.4 (68.3–81.1)	<0.001 (279)	1.27 (0.92–1.75)	73.3 (69.2–77.0)	0.148 (618)	1.78 (1.28–2.47)	65.7 (60.4–70.5)	0.001 (406)
N−Ch−	REF	97.1 (92.5–98.9)	0.955 (174)	1.03 (0.12–9.26)	94.1 (65.0–99.1)	0.976 (43)	NA	100	NA (3)	REF	92.4 (86.3-95.8)	0.395 (189)	1.69 (0.72-3.97)	87.5 (77.3-93.4)	0.232 (106)	0.69 (0.09-5.34)	94.7 (68.1-99.2)	0.721 (26)	REF	83.8 (70.1-91.6)	0.012 (95)	1.26 (0.61-2.59)	83.7 (77.1-91.6)	0.535 (200)	2.35 (1.15-4.78)	72.8 (63.4-80.2)	0.019 (134)
N+Ch−	REF	89.3 (84.4–92.7)	0.701 (263)	0.62 (0.19–2.07)	94.3 (83.4–98.1)	0.441 (54)	NA	100	NA (1)	REF	89.1 (84.1-92.5)	0.008 (309)	1.93 (1.09-3.43)	81.8 (73.1-87.9)	0.025 (142)	3.21 (1.32-7.80)	74.5 (51.7–87.7)	0.010 (25)	REF	70.3 (58.1–79.6)	<0.001 (111)	1.29 (0.80–2.09)	69.0 (61.4–75.4)	0.291 (207)	2.27 (1.41–3.65)	51.1 (41.5–59.8)	0.001 (135)
Ch+	REF	91.9 (86.5–95.2)	0.552 (205)	1.51 (0.63–3.60)	87.9 (76.9–93.9)	0.353 (77)	NA	100	NA (5)	REF	83.4 (76.0-88.7)	<0.001 (185)	1.72 (1.01-2.94)	76.2 (67.4-83.0)	0.046 (133)	3.47 (1.83-6.58)	56.4 (37.0–71.9)	<0.001 (38)	REF	71.7 (57.0–82.1)	0.644 (73)	1.24 (0.71–2.15)	67.4 (59.7–74.0)	0.445 (208)	1.06 (0.58–1.92)	73.8 (65.1–80.7)	0.854 (136)
	**Mammaprint-trained low**						**Mammaprint trained high**			**Mammaprint-trained low**						**Mammaprint-trained high**			**Mammaprint-trained low**						**Mammaprint-trained high**		
	**HR**	**DMFS**	P*				**HR**	**DMFS**	***P***	**HR**	**DMFS**	P*				**HR**	**DMFS**	***P***	**HR**	**DMFS**	P*				**HR**	**DMFS**	***P***
All cases	REF	92.2 (89.8–94.0)	0.753 (814)				1.37 (0.19–9.92)	90.0 (47.3–98.5)	0.754 (12)	REF	86.1 (83.3–88.5)	0.005 (944)				1.69 (1.16–2.44)	79.4 (72.6–84.7)	0.006 (211)	REF	78.1 (73.0–82.3)	<0.001 (422)				1.64 (1.26–2.12)	68.4 (64.9–71.7)	<0.001 (881)
N−Ch−	REF	96.6 (91.8–98.6)	0.763 (217)				NA	100	NA (3)	REF	93.1 (88.1–96.0)	0.031 (249)				2.47 (1.06–5.79)	83.8 (70.2–91.5)	0.037 (72)	REF	89.6 (80.6–94.5)	0.002 (133)				2.78 (1.43–5.44)	76.5 (70.6–81.4)	0.003 (296)
N+Ch−	REF	90.0 (85.7–93.1)	0.601 (315)				NA	100	NA (3)	REF	86.6 (82.2–90.0)	0.371 (415)				1.38 (0.68–2.84)	83.6 (70.7–91.1)	0.373 (61)	REF	75.7 (66.8–82.5)	<0.001 (166)				2.09 (1.41–3.10)	57.4 (50.8–63.5)	<0.001 (287)
Ch+	REF	91.1 (86.5–94.2)	0.280 (281)				2.88 (0.39–21.4)	75.0 (12.8–96.1)	0.302 (6)	REF	79.3 (73.3–84.2)	0.066 (278)				1.62 (0.96-2.73)	72.7 (60.8-81.5)	0.069 (78)	REF	69.0 (58.8-77.2)	0.948 (122)				0.99 (0.65-1.49)	70.9 (64.7-76.2)	0.948 (295)

HR = Hazard ratio. 95%CI = 95% confidence interval. P* = *p* value of log-rank test to compare survival distributions. REF = reference group. *P* = *p* value of Wald test for comparison versus reference (low risk) group. DMFS = distant metastasis free survival at 10 years (see text). (*N*) = number of cases in subgroups. All cases = all ER+ve/HER2-ve cases. N−Ch− = Node negative cases treated without chemotherapy. N+Ch− = Node positive cases treated without chemotherapy. Ch+= cases treated with chemotherapy (node negative and node positive combined). Int = intermediate.

### Prosigna-trained results stratified by Oncotype-trained results

In Prosigna-trained low-risk cases there were no significant differences in outcome across Oncotype-trained risk groups, all Prosigna trained low-risk cases experienced DMFS10 > 90% (Table 3; Fig. 3a). Similarly all Prosigna-trained high risk cases experienced a DMFS10 ≤ 80%; those that were also Oncotype-DX-trained high risk experienced significantly poorer outcome (DMFS10 = 65.7% 95%CI 60.4–70.5%, *p* < 0.001) than low or intermediate risk by Oncotype-trained (Table 3; Fig. 3c). Of 1155 Prosigna-trained intermediate-risk cases, 685 (59%) were classified low risk by the Oncotype-trained test (DMFS10 = 88.5%; *p* < 0.001), 89 cases (8%) were Oncotype-trained high risk (DMFS10 = 72.6%; *p* < 0.001, Table 3; Fig. 3b).

### Prosigna-trained stratified by MammaPrint-trained

Excluding Prosigna-trained intermediate-risk cases the majority of results (79.7%) remained in the same risk category (Supplementary Table 2). No stratification of Prosigna-trained low-risk cases occurred using MammaPrint-trained results (Table 3; Fig. 3a). All Prosigna-trained high-risk cases had DMFS10 < 80%, 32% were MammaPrint-trained low risk (Table 3; Fig. 3c). For Prosigna-trained intermediate-risk cases 18% were MammaPrint-trained high risk (DMFS10 = 79.4%; *p* = 0.005; Table 3, Fig. 3b).

### MammaPrint-trained

**Fig. 4:**
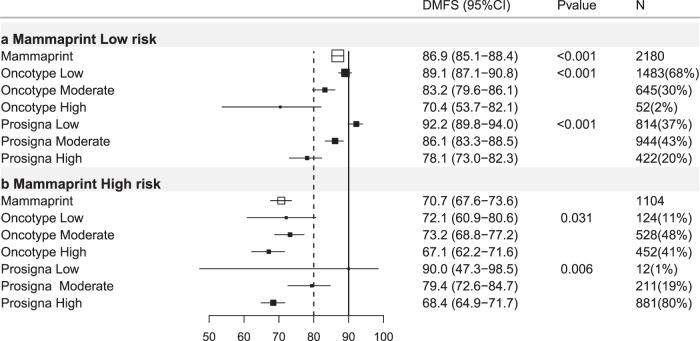
Forest plot of Mammaprint-trained test results re-stratified by other tests, all ER+ve/HER2-ve cases. DMFS10 = distant metastasis free survival at 10 years post diagnosis. (95% CI) = 95% confidence interval, *P* = *p* value, *N* = number of cases in each subgroup, % = percentage of cases within each risk strata, *X* axis = percent distant metastasis free survival. Open boxes represent primary test DMFS10 by risk group. Solid boxes represent sub-stratification by secondary tests with 95% confidence intervals (bars). Top panel (**a**) Mammaprint-trained low-risk cases stratified by Oncotype-trained and Prosigna-trained results. Bottom panel (**b**) Mammaprint-trained high-risk group.

Of 3284 ER+ve/HER2−ve breast cancers with MammaPrint-Trained risk classification, 66.3% were low risk (DMFS10 = 86.9%) and 33.7% high risk (DMFS10 = 70.7%; Table 4, Figs. 1c, 4).

**Table 4:** Mammaprint-trained results stratified by other test results, trinary classification.

	Mammaprint-trained first																	
	Mammaprint-trained low risk									Mammaprint-trained high risk								
	HR (95% CI)			DMFS (95% CI)			P* (*N*)			HR (95% CI)			DMFS (95% CI)			*P* (*N*)		
All cases	REF			86.9 (85.1–88.4)			<0.001 (2180)			2.64 (2.22–3.14)			70.7 (67.6–73.6)			<0.001 (1104)		
N-Ch−	REF			93.5 (90.5–95.6)			<0.001 (599)			4.23 (2.72–6.56)			78.2 (73.0–82.5)			<0.001 (371)		
N+Ch−	REF			85.9 (83.1–88.3)			<0.001 (896)			3.34 (2.56–4.36)			62.4 (56.5–67.7)			<0.001 (351)		
Ch+	REF			82.3 (78.8–85.3)			<0.001 (681)			1.86 (1.41–2.46)			71.2 (65.8–75.9)			<0.001 (379)		
	**Oncotype-trained low**			**Oncotype-trained Int**			**Oncotype-trained high**			**Oncotype-trained low**			**Oncotype-trained Int**			**Oncotype-trained high**		
	**HR**	**DMFS**	P*	**HR**	**DMFS**	***P***	**HR**	**DMFS**	***P***	**HR**	**DMFS**	P*	**HR**	**DMFS**	***P***	**HR**	**DMFS**	***P***
All cases	REF	89.1 (87.1–90.8)	<0.001 (1483)	1.74 (1.33–2.28)	83.2 (79.6–86.1)	<0.001 (645)	3.22 (1.82–5.69)	70.4 (53.7–82.1)	<0.001 (52)	REF	72.1 (60.9–80.6)	0.031 (124)	1.10 (0.72–1.68)	73.2 (68.8–77.2)	0.668 (528)	1.47 (0.97–2.24)	67.1 (62.2–71.6)	0.072 (452)
N−Ch−	REF	93.8 (90.0–96.2)	0.485 (407)	1.35 (0.62–2.92)	92.2 (85.6–95.8)	0.452 (174)	NA	100	NA (18)	REF	80.8 (62.8–90.7)	0.196 (51)	1.12 (0.49–2.56)	80.8 (73.2–86.4)	0.784 (175)	1.68 (0.74–3.80)	74.0 (65.0–81.0)	0.212 (145)
N+Ch−	REF	87.7 (84.4–90.3)	<0.001 (639)	1.49 (0.98–2.29)	84.2 (78.2–88.7)	0.064 (237)	5.14 (2.46–10.7)	54.2 (28.0–74.5)	<0.001 (20)	REF	67.6 (48.0–81.2)	0.047 (44)	1.22 (0.63–2.35)	66.9 (58.3–74.1)	0.552 (166)	1.83 (0.96–3.49)	55.3 (46.0–63.6)	0.067 (141)
Ch+	REF	86.8 (82.6–90.0)	<0.001 (434)	2.05 (1.38–3.06)	75.8 (69.1–81.2)	<0.001 (233)	3.52 (1.40–8.86)	61.1 (29.8–81.9)	0.007 (14)	REF	64.2 (37.6–81.8)	0.903 (29)	0.85 (0.40–1.81)	71.2 (63.0–77.9)	0.676 (185)	0.90 (0.42–1.92)	71.9 (63.8–78.4)	0.792 (165)
	**Prosigna-trained low**			**Prosigna-trained Int**			**Prosigna-trained high**			**Prosigna-trained low**			**Prosigna-trained Int**			**Prosigna-trained high**		
	**HR**	**DMFS**	P*	**HR**	**DMFS**	***P***	**HR**	**DMFS**	***P***	**HR**	**DMFS**	P*	**HR**	**DMFS**	***P***	**HR**	**DMFS**	***P***
All cases	REF	92.2 (89.8–94.0)	<0.001 (814)	1.77 (1.27–2.46)	86.1 (83.3–88.5)	0.001 (944)	3.01 (2.11–4.28)	78.1 (73.0–82.3)	<0.001 (422)	REF	90.0 (47.3–98.5)	0.006 (12)	2.17 (0.30–15.8)	79.4 (72.6–84.7)	0.446 (211)	3.57 (0.50–25.5)	68.4 (64.9–71.7)	0.204 (881)
N−Ch−	REF	96.6 (91.8–98.6)	0.069 (217)	2.24 (0.80–6.28)	93.1 (88.1–96.0)	0.126 (249)	3.37 (1.15–9.86)	89.6 (80.6-94.5)	0.027 (133)	REF	100	0.222 (3)	NA	83.8 (70.2-91.5)	NA (72)	NA	76.5 (70.6–81.4)	NA (296)
N+Ch−	REF	90.0 (85.7–93.1)	<0.001 (315)	1.28 (0.79–2.06)	86.6 (82.2–90.0)	0.315 (415)	2.65 (1.59–4.42)	75.7 (66.8–82.5)	<0.001 (166)	REF	100	0.002 (3)	NA	83.6 (70.7–91.1)	NA (61)	NA	57.4 (50.8–63.5)	NA (287)
Ch+	REF	91.1 (86.5–94.2)	<0.001 (281)	2.42 (1.45–4.03)	79.3 (73.3–84.2)	0.001 (278)	3.91 (2.26–6.79)	69.0 (58.8–77.2)	<0.001 (122)	REF	75.0 (12.8–96.1)	0.951 (6)	1.38 (0.19–10.3)	72.7 (60.8–81.5)	0.752 (78)	1.37 (0.19–9.83)	70.9 (64.7–76.2)	0.756 (295)

HR = hazard ratio, 95%CI = 95% confidence interval. P* = *p* value of log-rank test to compare survival distributions. REF = reference group. *P* = *p* value of Wald test for comparison versus reference (low risk) group. DMFS = distant metastasis free survival at 10 years (see text). (*N*) = number of cases in subgroups. All cases = all ER+ve/HER2-ve cases. N−Ch− = node negative cases treated without chemotherapy. N+Ch− = Node positive cases treated without chemotherapy. Ch+ = cases treated with chemotherapy (node negative and node positive combined). Int = intermediate.

### MammaPrint-trained stratified by Oncotype-trained

Of 2180 MammaPrint-trained low-risk cases, 68% were low risk by Oncotype-trained results (DMFS10 = 89.1%; Table 4; Fig. 4a). Mammaprint-trained low risk Oncotype-trained intermediate-risk cases (30%) exhibited DMFS10 = 83.2% (Table 4, *p* < 0.001) and Oncotype-trained high-risk cases exhibited DMFS10 = 70.4% (Table 4, *p* < 0.001; Fig. 4a). In MammaPrint-trained high-risk cases DMFS10 ranged from 73.2–67.3 across Oncotype-trained-subgroups and there were marked differences in outcome across Oncotype-trained categories (Table 4, Fig. 4b).

### MammaPrint-Trained results stratified by Prosigna-trained results

In MammaPrint-trained low-risk cases 20% were Prosigna-trained high risk (DMFS10 = 78.1%; Table 4, *p* < 0.001) and 43% intermediate risk (DMFS10 = 86.1% Table 4; *p* < 0.001, Fig. 4a). Amongst MammaPrint-trained high-risk cases, only a small (*n* = 12) subgroup of Mammaprint-trained high, Prosigna trained low results exhibited DMFS10 = 90% (*p* = 0.006, Fig. 4b).

### Sub-group analysis ER+ve/HER2-ve, Node-ve patients not treated with chemotherapy

#### Oncotype-trained

**Fig. 5:**
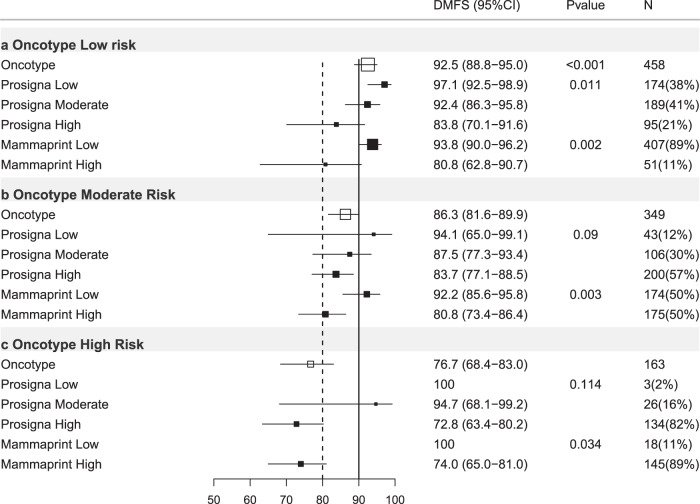
Forest plot of Oncotype-trained test results re-stratified by other tests, Node-ve ER+ve/HER2-ve cases treated without chemotherapy. DMFS10 = distant metastasis free survival at 10 years post diagnosis. (95% CI) = 95% confidence interval, *P* = *p* value, *N* = number of cases in each subgroup, % = percentage of cases within each risk strata. *X* axis = percent distant metastasis free survival. Open boxes represent primary test DMFS10 by risk group. Solid boxes represent sub-stratification by secondary tests with 95% confidence intervals (bars). Top panel (**a**) Oncotype-trained low-risk cases stratified by Prosigna-trained and Mammaprint-trained results. Middle panel (**b**) Oncotype-trained moderate risk group. Bottom panel (**c**) Oncotype-trained high-risk group.

Of 970 cases in this subgroup, 47.2% were Oncotype-trained low (DMFS10 = 92.5%), 36.0% intermediate (DMFS10 = 86.3%) and 16.8% high risk (DMFS10 = 76.7%, Table 2; Figs. 1d; 5) respectively.

#### Oncotype-trained results stratified by Prosigna-trained results

When Oncotype-trained results were stratified by Prosigna-trained results, 57.3% changed risk category (Supplementary Table 3). In Oncotype Dx-trained low risk 95 cases (21%) were Prosigna-trained high risk with DMFS10 = 83.8% (*p* = 0.006, Table 2; Fig. 5). In Oncotype-trained intermediate-risk cases 12% were Prosigna-trained low risk (DMFS10 = 94.1%; Table 2, *p* = 0.090; Fig. 5). The 57% of Oncotype-trained intermediate-risk cases classified as Prosigna-trained high risk exhibited DMFS10 = 83.7% (Table 2; *p* = 0.076, Fig. 5). Only three Oncotype-trained high-risk cases were Prosigna-trained low risk no events were observed in these cases.

#### Oncotype-trained stratified by MammaPrint-trained

11% of Oncotype-trained low-risk cases were MammaPrint-trained high risk (DMFS10 = 80.8%, *p* = 0.004; Table 2, Fig. 5a). In Oncotype-trained intermediate-risk patients 50% were MammaPrint-trained low risk(DMFS10 = 92.2%, *p* = 0.002; Table 2, Fig. 5b). In Oncotype Dx-trained high-risk cases 11% were MammaPrint-trained low risk, no events were observed in these 18 cases (Table 2, Fig. 5c). MammaPrint-trained scores identified 37.5% of Oncotype-trained cases (intermediate or high) as low risk (DMFS10 > 90%).

#### Prosigna-trained stratified by Oncotype-trained

**Fig. 6:**
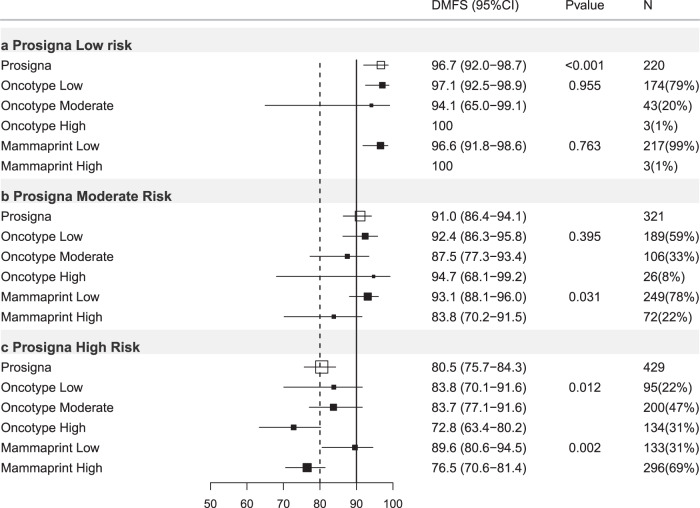
Forest plot of Prosigna-trained test results re-stratified by other tests, Node-ve ER+ve/HER2-ve cases treated without chemotherapy. DMFS10 = distant metastasis free survival at 10 years post diagnosis. (95% CI) = 95% confidence interval, *P* = *p* value, *N* = number of cases in each subgroup, % = percentage of cases within each risk strata, *X* axis = percent distant metastasis free survival. Open boxes represent primary test DMFS10 by risk group. Solid boxes represent sub-stratification by secondary tests with 95% confidence intervals (bars). Top panel (**a**) Prosigna-trained low-risk cases stratified by Oncotype-trained and Mammaprint-trained results. Middle panel (**b**) Prosigna-trained moderate risk group. Bottom panel (**c**) Prosigna-trained high risk group.

Neither Prosigna-trained low nor moderate risk cases showed statistically significant sub-stratification for outcome by Oncotype-trained risk scores (Table 3, Fig. 6a, b). Within Prosigna-trained high-risk cases 22% were Oncotype-trained low risk, however, DMFS10 for this group was 83.8% (Table 3, Fig. 6c).

#### Prosigna-trained stratified by MammaPrint-trained

No impact of MammaPrint-trained scores was observed in the Prosigna-trained low-risk group (Table 3, Fig. 6a), with only three discordant results. For both moderate and high risk Prosigna-trained results a group of MammaPrint-trained low-risk cases were identified (DMFS10 = 93.1% and 89.6%, respectively, Table 3; Fig. 6b, c).

### MammaPrint-trained results

**Fig. 7:**
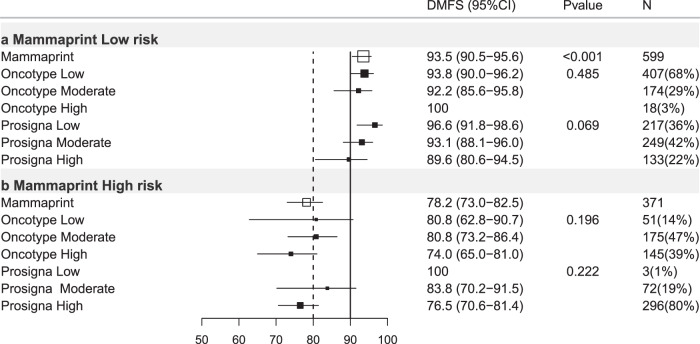
Forest plot of Mammaprint-trained test results re-stratified by other tests, Node-ve ER+ve/HER2−ve cases treated without chemotherapy. DMFS10 = Distant metastasis free survival at 10 years post diagnosis. (95% CI) = 95% confidence interval, *P* = *p* value, *N* = number of cases in each subgroup, % = percentage of cases within each risk strata, *X* axis = percent distant metastasis free survival. Open boxes represent primary test DMFS10 by risk group. Solid boxes represent sub-stratification by secondary tests with 95% confidence intervals (bars). Top panel (**a**) Mammaprint-trained low-risk cases stratified by Oncotype-trained and Prosigna-trained results. Bottom panel (**b**) Mammaprint-trained high-risk group.

No impact of Oncotype-trained on Mammaprint-trained scores was observed (Fig. 7; Table 4). In Mammaprint trained low-risk cases 22% were categorized as Prosigna-trained high risk, with a modest reduction in DMFS10 = 89.6% (*p* = 0.027, Table 4).

## Discussion

Our analysis of 3284 ER+ve/HER2−ve cases using trained signatures demonstrates that the Prosigna-trained signature provides potentially more prognostic information than either the Oncotype-trained or MammaPrint-trained signatures (Table 1). This result is consistent with results in the smaller TransATAC cohort^20^ using original vendor methodology.

Critical to our study is the close correlation between the computationally derived “signature trained” scores and true results as shown by us previously^18^. For ROR-PT results the correlation coefficient between “trained” and true assay results was 0.93, comparing true to “trained” results showed 90% of cases within the same risk category (low, intermediate, high—see ref. ^18^). Similarly for “Oncotype-Dx trained” results the correlation coefficient between true and “trained” results was 0.87 with 75% of results giving the same risk category (see ref. ^18^) and only 1% of cases disagreeing by more than 1 risk category. For Mammaprint trained results, which were calculated only as categorical high versus low risk groups, over 90% of cases were classified in the same risk group by “trained” and true results^18^. Full details of these results are reported elsewhere^18^.

We also show when two trained tests are combined the overall amount of information is always greater than a single test alone. In this study, adding stratification by Prosigna-trained results to Oncotype-trained results provided the greatest LR*χ*^2^, and the improvement was greater for this combined model versus Oncotype-trained results alone than for Prosigna-trained results alone. Collectively these results suggest that, in this study, Prosigna-trained results, either alone or combined with other test results, provide potentially greater prognostic information. However, most critically, all test combinations (where two tests were used for patient stratification) outperformed models with only one test to a highly statistically significant degree. This both confirms earlier reports^20^ and suggests that differences between tests reflect quantitative and qualitative differences in the degree of prognostic information collected. This conclusion is supported by recent comparisons by the ATAC group, showing the impact of different signaling modules in ER+ve/HER2−ve cases^21^ across different signatures. The conclusion from this work is that different tests capture different aspects of prognostic drivers and therefore that future improvements in prognostic testing remain achievable.

Critically, we dissected the effect of applying a second test to risk-stratified subgroups defined by the initial result; e.g. we examined the effect of applying the Prosigna-trained signature to the “intermediate risk” group identified by the Oncotype-trained signature etc. When combining tests, Prosigna-trained results added value to both Oncotype-trained and MammaPrint-trained results (Table 1). The improved prognostic impact of Prosigna-trained results applied across all ER+ve/HER2−ve cases after Oncotype-trained results was reflected by Prosigna-trained results sub-stratifying patients across both low and intermediate risk Oncotype trained groups (Fig. 2a, b). Even within the node negative ER+ve/HER2−ve population not treated with chemotherapy (Table 2; Fig. 5a, b) Oncotype-trained low and intermediate-risk groups were also further stratified by Prosigna-trained results and 20.7% of Oncotype-trained low-risk cases were identified as high risk by Prosigna-trained results, with DMFS10 of 83.8%, which is important as results from prospective trials suggest these cases may benefit from chemotherapy^2,6^. This difference was more striking when Oncotype-trained results were dichotomized using cut-points applied in the Tailor-X trial. In ER+HER2−ve, node negative patients treated without chemotherapy 17–24% of cases with Oncotype-trained results ≥25 were low risk (DMFS10 > 90%) when stratified by Mammaprint-trained or Prosigna-trained results respectively (Supplementary Table 4; Supplementary Fig. 2). Conversely 18–30% of Oncotype-trained low risk cases (<25) were high risk when stratified by Mammaprint-trained or Prosigna-trained results and exhibited DMFS < 90% (Supplementary Table 4; Supplementary Fig. 2)

Conversely, only in Prosigna-trained intermediate risk cases did Oncotype-trained results provide additional stratification by risk (Fig. 3; Table 3). However this stratification was not observed in the sub-group of node negative cases treated without chemotherapy (Fig. 6). No stratification of Prosigna-trained low or high risk cases was observed using either Oncotype-trained or Mammaprint trained results (Fig. 3; Table 3). When using dichotomized risk scores for Prosigna-trained ER+ve/HER2−ve node-negative cases treated without chemotherapy no further stratification using dichotomized Oncotype-trained results was seen (Supplementary Table 5; Supplementary Fig. 5) and all Prosigna-high risk cases exhibited DMFS10 < 85% regardless of dichotomized Oncotype-trained results (Supplementary Table 5; Supplementary Fig. 5). These results are illustrative of and highlight the potential clinical impact of disagreements between tests at an individual patient level previously demonstrated in the OPTIMA-prelim cohort^4^.

A number of conclusions that can be drawn from our analyses. Firstly that, as with previous analyses^20^ there is additional prognostic value to be gained from combining multiple molecular tests in the research setting. The corollary is that no single existing assay captures the sum of prognostic information available at the transcriptomic level. This confirms earlier findings^22^ that improvements in prognostic assays remain possible. Such improvements may, however, require integration of additional molecular features beyond transcriptomics^23,24^. Secondly, there was evidence, albeit from sub-group analyses, that the known interaction between clinical risk, treatment, and molecular risk profiling may differ depending on the test chosen. If taken at face value, this might provide support for the use of different testing strategies in different patient risk strata.

Our analysis has some potentially important limitations. In particular we have used a computational approach to generate test scores for the different tests described herein. At an individual tumor level, the trained score may not be identical to the equivalent generated using original methodology. We trained our signatures in an independent cohort using the same signatures measured using original methodology^18^, achieving extremely high correlations with commercial test results. Additionally, the broad agreement between our analysis with the(more limited) analysis of Sestak et al. ^20^ using original methodology and a slightly different statistical approach is highly reassuring.

Additionally, although our cohort is exclusively postmenopausal ER-positive, 30% of cases were treated with adjuvant chemotherapy. All patients in the TEAM trial were postmenopausal, with a median age of 64 years, results presented here may not be representative of the premenopausal population. We included chemotherapy-treated patients to maximize the power of our main analysis. However, the conclusions of our analysis performed on the node-negative subgroup who were not chemotherapy-treated are broadly similar to those in the analysis of the entire cohort, suggesting that these findings are robust both in this clinically critical node negative sub-group and indeed across all patients in the TEAM cohort.

The goal of our study was to provide robust information on the impact of discordant risk classification by different molecular prognostic signatures in postmenopausal, ER+ve early breast cancer. Existing evidence highlights discordance between tests^4,25^, which is reiterated here. There is clear evidence that adding clinical information to test results provides additional prognostic information^15,26–29^, which is supported by sub-group analyses performed here, and that information provided by any individual assay is relatively modest^17^. To date comparisons between tests have been limited either by relatively small sample sizes or by a lack of evidence that signatures extracted from global expression data reflect actual test performance and can therefore inform patients and clinicians on the impact of discordant test results on outcome in the real-world setting. This study provides data on a large clinical trial cohort (the TEAM trial) using test signatures trained in a second cohort (OPTIMA-prelim^4^) to match actual commercial test performance.

In summary, our study provides novel evidence for the potential clinical impact of discordant molecular test results in a large population. Further improvements in test performance are potentially within reach and would be of benefit to patients. Evidence presented here suggests the differences in test performance are more nuanced than previously reported and that careful consideration to test selection, in the context of treatment and clinical risk may be appropriate.

## Methods

### Study design

Our primary analyses explored the impact of signature-trained prognostic scores, categorized in accordance with published cut-points for each assay, for patients with centrally confirmed estrogen receptor positive (ER+ve) HER2 negative (HER2−ve) disease^30–32^. HER2 positive (HER2+ve) cases were excluded since during recruitment of the TEAM trial HER2 targeted therapies were not used in this setting. We performed a secondary analysis using dichotomized scores for Oncotype Dx and Prosigna to reflect the results of the TailorX study. We also report a complete cohort analysis, including HER2+ve cases (see Supplementary Information), since no assay used was trained on samples treated with HER2-targeted therapies. Supplementary analyses further sub-divide patient groups into node negative cases treated with endocrine therapy (but not chemotherapy), node positive cases treated with endocrine therapy (but not chemotherapy) and cases treated with chemotherapy and endocrine therapy (both node negative and node positive, supplementary methods, data and figures).

### Patient samples

Patient samples were derived from the Tamoxifen Exemestane Adjuvant Multicenter (TEAM) Trial pathology study (Supplementary Table 1; NCT00279448/NCT0032126/NCT0036270, NTR267, UMIN C000000057)^19,33^ and included only hormone receptor positive, post-menopausal cancers. Patients provided informed consent and this study was approved by the University of Toronto REB (protocol number 29021).

#### RNA profiling using NanoString

Profiling of all samples was performed using mRNA previously extracted and analyzed using a custom NanoString codeset as described previously^22^. Five 4 μm formalin-fixed paraffin-embedded (FFPE) sections per case were deparaffinised, tumor areas were macro-dissected and RNA extracted using the Ambion^®^ Recoverall™ Total Nucleic Acid Isolation Kit-RNA extraction protocol (Life TechnologiesTM, ON, Canada). RNA aliquots were quantified using a Nanodrop-8000 spectrophometer (Delaware, USA). All 3825 RNAs extracted from the TEAM pathology cohort were successfully assayed. Probes for each gene were designed and synthesized at NanoString^®^ Technologies (Seattle, WA, USA); and 250 ng of RNA for each sample were hybridized, processed and analyzed using the NanoString^®^ nCounter^®^ Analysis System, according to NanoString^®^ Technologies protocols.

### Signature-trained Risk Stratification Scores from candidate assays

We compared two different approaches to the generation of simulated risk scores^18^, and selected a training and validation approach using results obtained from the OPTIMA prelim study^4^ to fit risk stratification scores generated for this study to those derived from the relevant commercial assay. For all tests, we used the suffix-trained to discriminate the computationally derived assays scores from the commercially derived scores, e.g. Oncotype-trained vs. Oncotype-DX™.

### Methods for cross comparisons between Tests

Results were available for 3811 subjects. Cases were grouped into the pre-defined risk categories for each test as follows: Oncotype DX—low risk < 18, intermediate risk 18–31 (supplementary methods), high risk ≥ 31; Prosigna-ROR-PT—low risk < 41, intermediate risk 41–60, high risk ≥ 61^3,20,34^; MammaPrint—low risk and high risk^18^. We also performed a dichotomized risk analysis for Oncotype Dx using low/intermediate risk 0–25 and high risk > 25, in line with the TailorX study^2^, and for Prosigna RT using low/intermediate risk < 61 and high risk ≥ 61. Grouped analyses were performed as follows: (1) ER+/HER2−ve (*n* = 3284); and (2) hormone-receptor positive (HR+) regardless of HER2 status (*n* = 3811). Subjects were considered HR+ve if ER and/or progesterone receptor (PR) was reported as positive^33^. Differences in distant metastasis free survival (DMFS; i.e. time to first distant recurrence or death, excluding ipsilateral breast cancer recurrences but including distant metastasis, contralateral breast cancer and death from breast cancer) were evaluated using the Kaplan–Meier method with test equality of survivor functions assessed by log-rank and graphs with risk tables generated. 10-year survival function with 95% confidence intervals (95%CI) were calculated as DMFS10. Hazard ratios (HRs) were calculated using Cox proportional hazards regression models, with appropriate adjustments to obtain HRs for each risk level, with low risk set as reference. To assess the prognostic information of each signature, we evaluated the likelihood ratio *χ*^2^ (LR*χ*^2^) statistics based on the Cox models, and the difference in LR*χ*^2^(ΔLR*χ*^2^) was calculated to assess prognostic improvement. All analyses were performed using Stata 14.2 (StataCorp, College Station, TX) and R 4.0.2. Reported *p*-values were two-sided with *p* < 0.05 considered statistically significant.

### Reporting summary

Further information on research design is available in the Nature Research Reporting Summary linked to this article.

## Supplementary information

Supplementary Information

Reporting Summary

## Data Availability

The data generated and analyzed during this study are described in the following data record: 10.6084/m9.figshare.14617113^35^. The data generated and analyzed as part of this study take the form of 3811 individual Nanostring data files (one per sample). These data represent part of a clinical trial and were used under license for the current study, therefore restrictions apply to their availability. The data are housed in institutional storage at The Ontario Institute for Cancer Research (OICR) and are not publicly available, but can be made available upon request subject to approval from the TEAM steering committee and after appropriate data sharing agreements have been completed. Requests for data access should be directed to the senior author (J.M.S.B.).

## Code availability

The codes that support these findings are subject to patent applications and restrictions related to licenses. Codes are available from the author J.M.S.B. upon reasonable request and with the permission of the Ontario Institute for Cancer Research (OICR).
