# Supplementary material for: Comparative survival analysis of multiparametric tests—when molecular tests disagree—A TEAM Pathology study
Source: NPJ Breast Cancer. 2021 Jul 8;7:90. doi: 10.1038/s41523-021-00297-7 (PMC8266887; doi:10.1038/s41523-021-00297-7)

SUPPLEMENTARY METHODS:

TEAM (Trial design and Patients)

The TEAM trial is a multinational, randomised, open-label, phase III trial in which postmenopausal women with hormone receptor-positive luminal[1-4] early breast cancer were randomly assigned to receive exemestane (25 mg once daily), or tamoxifen (20 mg once daily) for the first 2.5-3 years followed by exemestane (total of 5 years treatment). This study complied with the Declaration of Helsinki, individual ethics committee guidelines, and the International Conference on Harmonisation and Good Clinical Practice guidelines; all patients provided informed consent. Distant metastasis free survival (DMFS) was defined as time from randomization to distant relapse or death from breast cancer or death from any cause following breast cancer recurrence[2]. The TEAM trial includes a pathology research study comprised of 4,736 patients from five countries with an average clinical follow-up of 6.86 years. RNA was available and successfully assayed from 3,825 samples[4] (Supplementary Table 1). Patients recruited to the TEAM trial[2] received a total of 5 years of ER targeted therapy, either 5 years of exemestane or 2.5 years of tamoxifen followed by 2.5 years of exemestane, no significant difference between these two regimens was observed, allowing the entire pathology cohort to be used to evaluate the impact of prognostic markers in the context of current endocrine and chemotherapies.

RNA extraction and mRNA analysis by NanoString:

Five 4 µm formalin-fixed paraffin-embedded (FFPE) sections per case were deparaffinised, tumor areas were macro-dissected and RNA extracted using the Ambion® Recoverall™ Total Nucleic Acid Isolation Kit-RNA extraction protocol (Life Technologies™, Ontario, Canada). RNA aliquots were quantified using a Nanodrop-8000 spectrophometer (Delaware, USA). All 3825 RNAs extracted from the TEAM pathology cohort were successfully assayed. Probes for each gene were designed and synthesised at NanoString® Technologies

(Seattle, Washington, USA); and 250 ng of RNA for each sample were hybridised, processed and analysed using the NanoString® nCounter® Analysis System, according to NanoString® Technologies protocols[4]

Generation and validation of "signature-trained" risk scores:

Previous studies comparing *in silico* generated risk signatures aimed to recapitulate performance of real world tests have been hampered by: a) incomplete gene coverage from some signatures and b) an inability to confirm the accuracy of simulated test results by comparison with data from actual test results.

We assessed two different approaches to the generation of simulated risk scores leading to the generation of two different simulated results for each of the multi-parametric tests examined. Both methods used data from all the representative genes (both those used for normalization and reporting) for each of the relevant tests. Method 2, resulting in scores regarded as "signature-trained", used a training and validation approach based on results obtained from the OPTIMA prelim study[5] and was shown in a parallel report to more accurately reflect actual test performance[6].

Signature trained risk stratification scores:

Performing analysis of the OPTIMA prelim samples using both commercial assays (OncotypeDx® (Genomic Health Inc.)[7,8], Prosigna™(NanoString Technologies, Inc.)[9,10], MammaPrint® (Agendia Inc.)[11] and our own gene profiling results[12] allowed us to use the OPTIMA-prelim cohort to train results for these multi-parametric assays. Briefly for each multiparametric assay (OncotypeDx®, Prosigna™, MammaPrint®) we divided the OPTIMA prelim cohort 50:50 randomly into training and validation sets. Information on the quantitative risk scores risk scores/sub-type classification in the training sets was used, in conjunction with the original

algorithms for each test and the quantitative mRNA expression levels determined by NanoString™ analysis[12], to establish the optimal fit between the NanoString expression data results and the commercial test results[6]. Once these approaches showed adequate performance in the training sets, we validated their performance in the validation set from OPTIMA prelim. For all commercial tests we used the suffix -trained to discriminate the computationally derived assays scores from the commercially derived scores, *e.g.* Oncotype-trained versus Oncotype-DX™.

*We used the following cut points 0-18, 18-<31 and $\geq$31 for the Oncotype Dx test in line with previous studies comparing tests[13,14]. To model reported results from the Tailor X trial[15,16] and our previous reported results from OPTIMA prelim[5] we dichotomized Oncotype Dx results using 25 as the cutpoint. For "Prosigna" results refer throughout to the ROR-PT risk score in clinical use, including tumour size, when used as a dichotomized score we combined low and intermediate risk groups.

SUPPLEMENTARY RESULTS

Methods for cross comparisons between Tests:

Results were available for 3,825 subjects for Oncotype-trained and MammaPrint-trained signatures, and 3,819 subjects for Prosigna-trained (six cases were missing tumour size which is required for calculation of the Prosigna score (ROR-PT)). Subsequent grouped analyses were performed as follows: (1) ER receptor positive (ER+) and HER2- (n=3,284); and (2) HR+ regardless of HER2 status (n=3,811). Subjects were considered HR+ if either ER or progesterone receptor (PR) was reported as positive (See Supplementary Table 1 for clinicopathological features of sub-groups used in this report). Differences in DMFS were evaluated using the Kaplan-Meier method with test equality of survivor functions assessed by log-rank. Kaplan-Meier graphs with risk tables were generated, with green representing low Risk, red representing high Risk, and blue

representing intermediate or moderate risk for Oncotype- and Prosigna-trained signatures, respectively. The 10-year survival function with 95% confidence intervals (95%CI) were also calculated and reported for DMFS. Hazard ratios (HR) were calculated using Cox proportional hazards regression models, with appropriate adjustments for Oncotype- and Prosigna-trained signatures to obtain HRs for each risk level, with low risk set as reference. To assess the prognostic information of each signature, we evaluated the likelihood ratio chi-squared ($LR\chi^2$) statistics based on the Cox models, and the difference in $LR\chi^2$ ($\Delta\ LR\chi^2$) was calculated to assess prognostic improvement; only those subjects who had "signature-trained" results for all three signatures evaluated were included for this analysis. All analyses were performed using Stata 14.2 (StataCorp, College Station, TX). Reported p-values were two-sided with $p<0.05$ considered statistically significant, no correction for multiple testing was applied.

BINARY TEST CATEGORIZATION:

Oncotype-trained results:

When Oncotype-trained results were stratified in accordance with cut-points used in the TailorX study 3284 cases with ER+ve/HER2-ve disease were divided into 72% low risk with DMFS10 of 85.8% and 28% high risk with DMFS10 70.2% (Supplementary Table 4; Supplementary Figure 1A, Supplementary Figure 2).

Stratification by test in all ER+/HER2-ve cases:

In cases with Oncotype-trained scores <25, 26% had Prosigna-trained scores $\geq$61 (high risk) with DMFS10 of 76.5%, 74% had Prosigna-trained scores <61 with DMFS10 89.0% ($p<0.001$; Supplementary Table 4; Supplementary Figure 2. In cases with Oncotype-trained scores $\geq$25, 26% exhibited Prosigna-trained scores <61 with DMFS10 79.6%, whilst those with Prosigna-trained

scores ≥61 showed DMFS10 of 66.9% (p<0.001; Supplementary Table 4; Supplementary Figure 2).

For Oncotype-trained scores <25, 16% were classified as high risk using Mammaprint-trained results with DMFS10 of 74.3% (p<0.001; Supplementary Table 4; Supplementary Figure 2). The remaining cases were Mammaprint-trained low with DMFS10 of 88.0% (p<0.001; Supplementary Table 4; Supplementary Figure 2). In Oncotype-trained high risk cases (≥25), modest stratification by Mammaprint-trained low vs high risk was observed (Supplementary Table 4; Supplementary Figure 2).

Stratification by test in ER+/HER2-ve Node-ve cases treated without chemotherapy:

In the sub-group of ER+ve/HER2-ve, node-negative cases treated without chemotherapy, 69.5% exhibited Oncotype-trained scores <25 with DMFS10 of 90.8%, 30.5% were Oncotype-trained high risk with DMFS10 80.2% (Supplementary Table 4, Supplementary figure 3).

In cases with Oncotype-trained scores <25, 30% were Prosigna-trained ≥61 with DMFS10 84.7, the remaining cases were Prosigna-trained <61 with DMFS10 93.4% (p<0.001; Supplementary Table 4, Supplementary Figure 3). In Oncotype-trained high risk cases (≥25), 24% were Prosigna-trained low risk (<61) with DMFS10 of 92.1%, versus 76% Prosigna-trained high risk with DMFS10 of 76.5% (p=0.013; Supplementary Table 4, Supplementary Figure 3).

When Oncotype-trained scores were <25, 19% were classified as high risk using Mammaprint-trained results with DMFS10 of 80.2%. The remaining cases were Mammaprint-trained low risk (DMFS10=93.3%, p<0.001; Supplementary Table 4; Supplementary Figure 3). In Oncotype-trained high risk cases (≥25), 17% were Mammaprint-trained low risk (DMFS10 95.5%) 83% were Mammaprint-trained high risk (DMFS10 77.1%, p=0.018; Supplementary Table 4; Supplementary Figure 3).

Prosigna-trained results:

When Prosigna-trained results were stratified as a binary distribution cases with ER+ve/HER2-ve disease were divided into 60.3% low risk with DMFS10 of 87.9% and 39.7% high risk with DMFS10 71.4 % (Supplementary Table 5; Supplementary Figure 1, Supplementary Figure 4).

Stratification of Prosigna-trained in all ER+/HER2-ve cases

In Prosigna-trained cases with scores <61, only 12% exhibited Oncotype-trained scores ≥25 with DMFS10 79.6% vs 88% with scores <25 and DMFS10 of 89.0% (p<0.001; Supplementary Table 5: Supplementary Figure 4). All Prosigna-trained high risk cases exhibited DFMS10<80%; 48% were Oncotype-trained <25 (DMFS10 76.5%) vs 52% ≥25 (DMFS10 66.9%, p<0.001; Supplementary Table 5: Supplementary Figure 4).

For Prosigna-trained cases with scores <61, 11% were Mammaprint-trained high risk (DMFS10 80.0%) vs. 89% Mammaprint-trained low risk (DMFS10 88.9%; p<0.001 Supplementary Table 5; Supplementary Figure 4). When stratified by Mammaprint-trained results, all Prosigna-trained high risk cases exhibited DMFS10 <80%, 32% were Mammaprint-trained low risk (DMFS10 78.1%) vs 68% Mammaprint-trained high risk (DMFS10 68.4%; p<0.001; Supplementary Table 5; Supplementary Figure 4).

Stratification of Prosigna-trained in ER+/HER2-ve Node-ve cases treated without chemotherapy:

When stratified by Oncotype-trained results all Prosigna-trained cases with scores <61 exhibited DMFS10 >90%, 87% were Oncotype-trained <25 (DMFS10 93.4%) vs 13% Oncotype-trained ≥25 (DMFS10 92.1%, p=0.419; Supplementary Table 5; Supplementary Figure 5). Again stratifying by Oncotype-trained results, all Prosigna-trained cases with scores ≥61 exhibited

DMFS10 <85%, 48% were Oncotype-trained <25 (DMFS10 84.7%) vs. 52% Oncotype-trained ≥25 (DMFS10 76.5% p=0.013; Supplementary Table 5: Supplementary Figure 5).

When stratified by Mammaprint-trained results, 14% of Prosigna-trained low risk cases were Mammaprint-trained high risk (DMFS10 84.6%) vs 86% low risk by both tests (DMF10 94.7%; p=0.005; Supplementary Table 5; Supplementary Figure 5). When stratified by Mammaprint-trained results, all Prosigna trained high risk cases exhibited DMFS10 <90%, 31% were Mammaprint-trained low risk (DMFS10 89.6%) and 69% Mammaprint trained high risk (DMFS10 76.5%; p=0.003; Supplementary Table 5: Supplementary Figure 5).

Mammaprint-trained results:

Stratification of Mammaprint-trained in all ER+/HER2-ve cases

In Mammaprint-trained low risk cases, 9% were Oncotype-trained high risk (DMFS10 75.9%) and 91% low risk by both tests (DMFS10 88.0%; p<0.001; Supplementary Table 6; Supplementary Figure 6). Amongst Mammaprint-trained high risk cases stratified by Oncotype-trained results DMFS10 was <75%, 34% were Oncotype-trained low risk (DMFS10 74.3%) and 66% high risk by both tests (DMFS10 68.7%; p=0.006; Supplementary Table 6; Supplementary Figure 6).

When Mammaprint-trained low risk cases were stratified by Prosigna-trained results, 19% were Prosigna-trained high risk (DMFS10 78.1%) and 81% low risk by both tests (DMFS10 89.9%; p<0.001; Supplementary Table 6; Supplementary Figure 6). In Mammaprint-trained high risk cases, stratified by Prosigna-trained results, DMFS10 was ≤80%; 20% were Prosigna-trained low risk (DMFS10 80.0%) and 80% were high risk by both tests (DMFS10 68.4%; p=0.002; Supplementary Table 6; Supplementary Figure 6).

Stratification of Mammaprint-trained in ER+/HER2-ve Node-ve cases treated without chemotherapy:

In Mammaprint-trained low risk cases no significant difference in outcome was observed between Oncotype-trained low and high risk cases, all cases exhibiting DMFS10 >90% (p=0.799; Supplementary Table 6; Supplementary Figure 7). Similarly in Mammaprint-trained high risk cases no significant difference in outcome was observed between Oncotype-trained low and high risk cases (p=0.248; Supplementary Table 6; Supplementary Figure 7).

Also for Mammaprint-trained low risk cases, stratified by Prosigna-trained results, although in Mammaprint-trained low risk/Prosigna-trained high risk cases DMFS10 was 89.6% this was not significantly lower than DMFS10 observed in cases low risk by both tests (DMFS10 94.7%; p=0.074). In Mammaprint-trained high risk cases no significant difference in outcome was observed between Prosigna trained low and high risk cases (Supplementary Table 6; Supplementary Figure 7).

SUPPLEMENTARY RESULTS: TRINARY CLASSIFICATION

"Oncotype trained" results stratified by Prosigna-trained results (ER+ve/HER2-ve Sub-group analyses):

Comparison of the performance of "signature-trained" risk score results in different sub-groups is compromised by a reduction in statistical power. However, there is a suggestion that clinical risk stratification may impact the performance of tests since it remains a consistent modifier of patient risk even in the context of most molecular prognostic assays. Given the relatively large number of patients in the TEAM study it was possible to extend the cross-test comparisons into three sub-groups:

a) Patients with ER+ve/HER-ve node negative disease treated without adjuvant chemotherapy:

See main Manuscript.

b) Patients with ER+ve/HER-ve node positive disease treated without adjuvant chemotherapy:

Of 1268 cases in this subgroup, 53.9% were Oncotype-trained low (DMFS10=86.4%), 31.9% intermediate (DMFS10=76.8%) and 14.3% high risk respectively (DMFS10=55.1%, Table 2; Supplementary Figure 8A).

Oncotype-trained results stratified by Prosigna-trained results:

When Oncotype-trained results were further stratified by Prosigna-trained results 42.6% remained in the same risk category (Supplementary Table 7). Here 8.8% of "Oncotype- trained" low risk cases were Prosigna-trained high risk (DMFS10 70.3%, p<0.001 Table 2; Supplementary Figure 9A). No significant difference in DMFS10 was observed between Oncotype-trained low risk cases categorized as Prosigna-trained low versus intermediate risk (Table 2). In "Oncotype- trained" intermediate risk cases 13.4% were Prosigna-trained low risk (DMFS10 94.3%, p=0.0002, Table 2, Supplementary Figure 9B). Also 35.1% "Oncotype -trained" intermediate risk patients were Prosigna-trained high risk (DMFS10 69.0%, p=0.003, Table 2, Supplementary Figure 9B). However, only 1 "Oncotype -trained" high risk case was Prosigna-trained low risk (Table 2, Supplementary Figure 9C). Overall 9.7% of all "Oncotype -trained" intermediate and high risk cases were Prosigna-trained low risk with DMFS10 >90%. A further 29.3% of all "Oncotype -trained" intermediate and low risk cases were Prosigna-trained high risk with DMFS10 of <71.0%.

c) Patients with ER+ve/HER-ve disease treated with chemotherapy (Node positive or negative).

The performance of "Oncotype -trained" results in this subgroup are represented in Supplementary Figure 10A.

Oncotype-trained results stratified by Prosigna-trained results:

When Oncotype-trained results were further stratified by Prosigna-trained results 474 cases (44.7%) remained in the same risk category (Supplementary Table 8). In "Oncotype -trained" low

risk cases 15.7% were Prosigna-trained high risk (DMFS10 71.7%, p<0.001, Supplementary Figure 11A). In “Oncotype -trained” intermediate risk cases 18.4% were Prosigna-trained low risk (DMFS10 87.9%, 95% C.I. 76.9-93.9%, p=0.007); Supplementary Figure 11B). A further 51% of “Oncotype-Dx-trained” intermediate risk cases were Prosigna-trained high risk (DMFS10 67.4%; 95% C.I.59.7-74.0%; Supplementary Figure 11). Finally, 2.8% of “Oncotype- trained” high risk cases were Prosigna-trained low risk (DMFS 100%; Supplementary Figure 11). Overall 13.7% of all “Oncotype -trained” cases (intermediate and high risk) were Prosigna-trained low risk with DMFS10 of >90.0%. A further 31.9% of all cases (“Oncotype -trained” intermediate and low risk) were Prosigna-trained high risk (DMFS10 <75.0%).

“Oncotype -trained” results stratified by Mammaprint-trained results (ER+ve/HER2-ve Sub-group analyses):

a) Patients with ER+ve/HER-ve node negative disease treated without adjuvant chemotherapy:

   See main Manuscript.

b) Patients with ER+ve/HER-ve node positive disease treated without adjuvant chemotherapy:

In “Oncotype -trained” low risk cases 6.4% were Mammaprint-trained high risk (DMFS10 67.6%, 95% C.I. 48.0-81.2%; p=0.001, Table 2; Supplementary Figure 9). In Oncotype-trained intermediate risk cases 41.0% were Mammaprint-trained high risk (DMFS10 66.9%, 95% C.I. 58.3-74.1, p<0.001, Table 2; Supplementary Figure 9). No stratification of Oncotype-trained high risk cases was observed (Supplementary Figure 9). Mammaprint-trained high risk scores identified 23.7% of “Oncotype-Dx-trained” low or intermediate risk cases as high risk with DMFS10 of <70%.

c) **Patients with ER+ve/HER-ve disease treated with chemotherapy (Node positive or negative).**

In "Oncotype -trained" low risk cases 6.3% were Mammaprint-trained high risk (DMFS10 64.2%, 95% C.I. 37.6-81.8%; p=0.004, Table 2; Supplementary Figure 11A). There was no evidence that sub-stratifying Oncotype-trained scores by Mammaprint-trained scores identified any significant differences across intermediate or high risk patients (Table 2; Supplementary Figure 11B-C).

Prosigna-trained results stratified by Oncotype-trained results (ER+ve/HER2-ve Sub-group analyses):

a) **Patients with ER+ve/HER-ve node negative disease treated without adjuvant chemotherapy:**

   See main manuscript.

b) **Patients with ER+ve/HER-ve node positive disease treated without adjuvant chemotherapy:**

Amongst Prosigna-trained low risk cases only 1 case was Oncotype-trained high risk (Table 3; Supplementary Table 8; Supplementary Figure 12A). For cases classified as Prosigna-trained high risk no group of Oncotype-trained cases exhibited DMFS10 >75% (Table 3; Supplementary Figure 12C). Amongst the 38.2% of cases classified as Prosigna-trained intermediate risk, 64.9% were Oncotype-trained low risk with a DMFS10 marginally below 90% (DMFS10 89.1%, 95% C.I. 84.1-92.5%) and 5.3% were Oncotype-trained high risk with DMFS <75% (DMFS10 74.5%, 95% C.I. 51.7-87.7%, p=0.010; Table 3; Supplementary Figure 12B).

c) **Patients with ER+ve/HER-ve disease treated with adjuvant chemotherapy (node –ve and node positive):**

No statistically significant stratification of Prosigna-trained low or high risk cases was observed using "Oncotype-Dx-trained" risk scores (Supplementary Figure 13A and C). In Prosigna-trained intermediate risk cases 52.0% were Oncotype-trained low risk with DMFS10 <90% (DMFS10 83.4%, 95% C.I. 76.0-88.7%) and 10.7% were Oncotype-trained high risk with DMFS10 <60% (DMFS10 56.4%, 95% C.I. 37.0-71.9%, p<0.001; Table 3; Supplementary Table 8; Supplementary Figure 13B).

Prosigna-trained results stratified by Mammaprint-trained results (ER+ve/HER2-ve Sub-group analyses):

a) **Patients with ER+ve/HER-ve node negative disease treated without adjuvant chemotherapy:**

See main manuscript

b) **Patients with ER+ve/HER-ve node positive disease treated without adjuvant chemotherapy:**

No statistically significant stratification was observed in Prosigna-trained low risk cases, where only 3 cases were Mammaprint-trained high risk or in intermediate risk cases (Table 3; Supplementary Figure 12A & B). In Prosigna-trained high risk cases, Mammaprint-trained results split cases into two groups both of which exhibited DMFS10 <80% (DMFS10 75.7% and 57.4% respectively; Table 3; Supplementary Figure 12C).

c) **Patients with ER+ve/HER-ve disease treated with chemotherapy (Node positive or negative).**

For Prosigna-trained low, intermediate and high risk cases there was no evidence that sub-stratifying by Mammaprint-trained scores identified significant differences (Table 3; Supplementary Figure 13A-C).

Mammaprint-trained results stratified by Oncotype-trained results: Sub group analyses:

a) **Patients with ER+ve/HER-ve node negative disease treated without adjuvant chemotherapy:**

See main manuscript.

b) **Patients with ER+ve/HER-ve node positive disease treated without adjuvant chemotherapy:**

Amongst Mammaprint-trained low risk cases only 2.2% were Oncotype-trained high risk (DMFS10 54.2%, 95% C.I. 28.0-74.5%, p<0.001, Table 4; Supplementary Figure 14A). Cases classified as Mammaprint-trained high risk across all Oncotype-trained risk categories exhibited DMFS10 <80% (Table 4; Supplementary Figure 14B).

c) **Patients with ER+ve/HER-ve disease treated with adjuvant chemotherapy (node –ve and node +ve):**

In patients who received chemotherapy, irrespective of nodal status, no further stratification was observed using the "Oncotype-Dx-trained" risk scores in patients classified as Mammaprint-trained high risk (Supplementary Figure 15B). Further stratification using "Oncotype-Dx-trained" risk scores appeared confined to cases classified as low risk by Mammaprint-trained results (Supplementary Figure 15A). 63.7% of these cases were classified as "Oncotype -trained" low risk with DMFS10 of 86.8% (95% C.I. 82.6-90.0%), 26.5% of Mammaprint-trained low risk cases were classified as "Oncotype -trained" intermediate risk with DMFS10 of 75.8% (95% C.I. 69.1-81.2%), finally 14 (1.6%) cases were classified as "Oncotype- trained" high risk with DMFS10 of 61.1% (95% C.I. 29.8-81.9%; Table 4; Supplementary Figure 15A).

Mammaprint-trained results stratified by Prosigna-trained results: Sub-group analyses

a) **Patients with ER+ve/HER-ve node negative disease treated without adjuvant chemotherapy:**

   See main manuscript.

b) **Patients with ER+ve/HER-ve node positive disease treated without adjuvant chemotherapy:**

In Mammaprint-trained low risk cases 18.5% were classified as high risk by Prosigna-trained results (DMFS10 75.7% 95% C.I. 66.8-82.5%; p<0.001; Table 4; Supplementary Figure 14A). In Mammaprint-trained high risk cases only 3 cases were Prosigna-trained low risk and a small group of Prosigna-trained moderate risk score cases exhibited improved outcome when compared to cases classified as high risk by both scores (DMFS10 83.6% 95% C.I. 70.7-91.1%; Table 4; Supplementary Figure 14B).

c) Patients with ER+ve/HER-ve disease treated with chemotherapy (Node positive or negative).

Across patients in Mammaprint-trained low risk categories Prosigna-trained results identified cases with moderate risk (40.7%) and high risk (17.9%) with DMFS10 of 79.3 & 69.0% respectively (Table 4; Supplementary Figure 15A). There was no evidence that sub-stratifying Mammaprint-trained high scores by Prosigna-trained scores identified significant differences (Supplementary Figure 15B).

Supplementary Discussion: Sub-group analyses

The key findings of this paper are reviewed in the main discussion, herein we are focused on particular nuances which may apply to sub-groups analyzed as part of the supplementary data presented above. As pointed out in many settings, sub-group analyses must be interpreted with caution, due to reduce power, multiple testing etc. As a result, we recognize that the interpretation

of these sub-group analyses is limited and strongly recommend readers review the data presented and draw their own conclusions.

Overall prognostic impact of different tests across subgroups:

Each of the tests studied provided clear separation of patients into low, intermediate (where applicable) and high risk strata. As expected and consistent with previous data, there are differences in DMFS by nodal status. For example in node negative low risk cases treated without chemotherapy 10 year DMFS ranges from 92.5-96.7% (dependent on test; Tables 2-4). For node positive cases, also treated without chemotherapy, the range is 85.9-90.1% (Tables 2-4). For high risk cases, the difference is more stark, with DMFS10 for node negative cases, treated without chemotherapy, ranging from 76.7-80.5% and for node positive cases from 55.1-63.9%. However, differences between risk estimates when HER2+ve cases are included or excluded are too small to be regarded as impactful (See below). Overall this data provides further support for interpretation of molecular signatures in the context of other clinicopathological variables. Increasing integration of clinicopathological variables into the development of future prognostic and predictive assays is recommended.

Oncotype Trained results stratified by Prosigna-trained and MammaPrint-Trained results:

Both Prosigna-trained and Mammaprint-trained results identified cases categorized as low risk by Oncotype-trained results, in both node negative and node positive patients treated with or without chemotherapy (Figure 5 & Supplementary Figures 9, 11), that were high risk. Further stratification of Oncotype-trained moderate risk cases by both Prosigna-trained and Mammaprint-trained results were also consistent between node negative and node positive cases, again irrespective of chemotherapy treatment (Figure 5 & Supplementary Figures 9, 11). This evidence suggests that both Oncotype-trained intermediate risk and low risk groups are markedly stratified further, by

other prognostic tests, and that this stratification is clinically meaningful identifying additional high risk cases, as evidenced by survival outcome, in Oncotype-trained intermediate and low risk cases, and further low risk cases in Oncotype-trained intermediate cases across all subgroups. Only in Oncotype-trained high risk cases was this not observed.

Overall for results observed when Oncotype-trained results were stratified by Prosigna-trained or MammaPrint-trained results were compared in subgroup analyses versus the main analyses reported in the main paper we saw minimal evidence to suggest the overarching conclusions were different in different patient sub-populations. Statistical error, or reduced statistical power when exploring small subgroups of cases within these stratified analyses remains the most likely explanation for any observed differences. However, further evidence for the true risk categorization of Oncotype-trained high risk Prosigna-trained low risk patients, and conversely Oncotype-trained low risk Prosigna trained high risk cases in node negative patients would be of value before such results are used to alter current approaches to risk stratification.

Prosigna Trained results stratified by Oncotype-trained and MammaPrint-Trained results:

When cases were stratified first by Prosigna-trained results neither Oncotype-trained nor Mammaprint-trained results produced further stratification of Prosigna-trained low risk cases, irrespective of nodal status (Figure 6, Supplementary Figure 12) or chemotherapy treatment (Supplementary Figure 13). In Prosigna-trained high risk cases, no marked stratification was seen using Oncotype-trained results across these subgroups (Figure 6, Supplementary Figure 12-13), although consistently cases deemed high risk by multiple tests experienced worse outcome. However, solely in node negative patients treated without chemotherapy classified as high risk by Prosigna-trained results, Mammaprint-trained low risk cases, experienced improved outcome (Figure 6), this result was not consistent in either chemotherapy naïve node positive patients

(Supplementary Figure 12) or chemotherapy treated cases (Supplementary Figure 13). This pattern was maintained (Figure 6; Supplementary Figures 12-13) for Prosigna-trained intermediate risk cases further stratified by Mammaprint-trained results. Conversely, only in Prosigna-trained intermediate risk cases, and only in node positive and chemotherapy treated subgroups, Oncotype-trained results correctly identified a small proportion (<10%) of high risk cases.

Focusing on sub-group analyses would suggest that in node negative, chemotherapy naïve cases, Mammaprint-trianed results may further stratify both intermediate and high risk Prosigna-trained results, consistent with this being the primary population for which this test was developed. However this would potentially also reflect a sub-group effect prone to increased statistical error. Conversely, the impact of Oncotype-trained results, whilst remaining consistently confined to Prosigna-trained intermediate risk cases, shows minimal effect in node negative chemotherapy naïve disease, and seems confined to a smaller group of node positive, or chemotherapy treated cases. Caution should be taken here since, in the more powered main analyses, the impact of Oncotype-trained results appears to be to clearly sub-stratify Prosigna-trained intermediate risk cases.

MammaPrint trained results stratified by Oncotype-trained and Prosigna-Trained results:

Amongst Mammaprint-trained high risk cases, sub-group analyses show no further stratification by Oncotype-trained results irrespective of sub-groups (Figure 7, Supplementary Figures 14-15). Broadly similar results are obtained using Prosigna-trained results in Mammaprint-trained high risk cases, although a small group of Prosigna-trained low risk cases were observed (DMFS10 83.6%) in node positive cases treated without chemotherapy (Supplementary Figure 14). In node negative chemotherapy naïve Mammaprint-low risk cases Oncotype-trained results did not produce further stratification, and whilst Prosigna-trained results did some some evidence of

further sub-stratification the effect was modest (Figure 7). However in node positive chemotherapy naïve (Supplementary Figure 14) and chemotherapy treated cases (Supplementary Figure 15) both Oncotype-trained and Prosigna-trained results showed marked, clinically meaningful further stratification of Mammaprint-trained low risk cases. Whilst both Oncotype-trained and Prosigna-trained results indentified a subset of Mammaprint-trained cases with DMFS10<80% in these sub-groups, the proportions were strikingly different, with 2% of cases being Oncotype-trained high risk versus 18% of cases classified as high risk by Prosigna-trained results (Supplementary Figures 14-15). Prosigna-trained results also identified a greater proportion of intermediate risk cases than Oncotype-trained results in this subgroup (Supplementary Figures 14-15).

Again this evidence suggests that Mammaprint-trained results perform better in chemotherapy naïve node negative cases, whilst in both chemotherapy naïve node positive disease and cases treated with chemotherapy, some further risk stratification by additional tests produces meaningful results. Here there does seem to be some evidence for differential performance, particularly of the Mammaprint-trained results, depending on both clinicopathological features and treatment decisions arising.

Since MammaPrint™ was originally trained in patients with node negative disease, who received no systemic therapy, this result is consistent with the development pattern of this test and may therefore reflect a true difference in test performance between clinical sub-groups. Further analyses are required to substantiate this observation.

Supplementary Material references;

1. Bartlett JM, Brookes CL, Piper T, et al: Do type 1 receptor tyrosine kinases inform treatment choice? A prospectively planned analysis of the TEAM trial. Br. J Cancer 109:2453-2461, 2013
2. van de Velde CJH, Rea D, Seynaeve C, et al: Adjuvant tamoxifen and exemestane in early breast cancer (TEAM): a randomised phase 3 trial. Lancet 377:321-331, 2011
3. Bartlett JMS, Brookes CL, Robson T, et al: Estrogen Receptor and Progesterone Receptor As Predictive Biomarkers of Response to Endocrine Therapy: A Prospectively Powered Pathology Study in the Tamoxifen and Exemestane Adjuvant Multinational Trial. Journal of Clinical Oncology 29:1531-1538, 2011
4. Bayani J, Yao CQ, Quintayo MA, et al: Molecular stratification of early breast cancer identifies drug targets to drive stratified medicine. npj Breast Cancer 3:3, 2017
5. Bartlett JM, Bayani J, Marshall A, et al: Comparing Breast Cancer Multiparameter Tests in the OPTIMA Prelim Trial: No Test Is More Equal Than the Others. J. Natl. Cancer Inst 108:djw050, 2016
6. Bartlett JMS, Bayani J, Kornaga EN, et al: Computational approaches to support comparative analysis of multiparametric tests: Modelling versus Training. PloS one 15:e0238593-e0238593, 2020
7. Paik S, Shak S, Tang G, et al: A multigene assay to predict recurrence of tamoxifen-treated, node-negative breast cancer. N. Engl. J. Med 351:2817-2826, 2004
8. Paik S, Tang G, Shak S, et al: Gene Expression and Benefit of Chemotherapy in Women With Node-Negative, Estrogen Receptor-Positive Breast Cancer. Journal of Clinical Oncology 24:3726-3734, 2006
9. Chia SK, Bramwell VH, Tu D, et al: A 50-Gene Intrinsic Subtype Classifier for Prognosis and Prediction of Benefit from Adjuvant Tamoxifen. Clinical Cancer Research 18:4465-4472, 2012
10. Nielsen TO, Parker JS, Leung S, et al: A Comparison of PAM50 Intrinsic Subtyping with Immunohistochemistry and Clinical Prognostic Factors in Tamoxifen-Treated Estrogen Receptor-Positive Breast Cancer. Clinical Cancer Research 16:5222-5232, 2010
11. van't Veer LJ, Dai HY, van de Vijver MJ, et al: Gene expression profiling predicts clinical outcome of breast cancer. Nature 415:530-536, 2002
12. Bayani J, Yao CQ, Quintayo MA, et al: Molecular stratification of early breast cancer identifies drug targets to drive stratified medicine. npj Breast Cancer 3:3, 2017
13. Sestak I, Buus R, Cuzick J, et al: Comparison of the Performance of 6 Prognostic Signatures for Estrogen Receptor–Positive Breast Cancer: A Secondary Analysis of a Randomized Clinical TrialPrognostic Signatures for Estrogen Receptor–Positive Breast CancerPrognostic Signatures for Estrogen Receptor–Positive Breast Cancer. JAMA Oncology 4:545-553, 2018
14. Sestak I, Ferree S, Shemesh I, et al: Abstract P5-06-05: Discordant classification and outcomes between Prosigna and Oncotype Dx Recurrence Score for ER-positive, HER2-negative, node-negative breast cancer. Cancer Research 80:P5-06-05, 2020
15. Sparano JA, Gray RJ, Ravdin PM, et al: Clinical and Genomic Risk to Guide the Use of Adjuvant Therapy for Breast Cancer. New England Journal of Medicine 380:2395-2405, 2019
16. Sparano JA, Gray RJ, Makower DF, et al: Adjuvant Chemotherapy Guided by a 21-Gene Expression Assay in Breast Cancer. New England Journal of Medicine 379:111-121, 2018

Comparative survival analysis of multiparametric tests -when molecular tests disagree- A TEAM Pathology study:

ANALYSIS OF ALL HR+VE CASES BOTH HER+VE AND HER-VE

Populations available for cross comparisons between Tests:

All analysis were performed as described in the main paper and supplementary methods. Subjects were considered HR+ if either ER or progesterone receptor was reported as positive. In this analysis both HER2+ve and HER2-ve cases were included, whilst in the analyses reported in the main paper and supplement HER2+ve cases were excluded. The rationale for exclusion of HER2+ve cases was that these cases were recruited prior to the widespread adoption of therapies targeted against HER2 in the adjuvant setting.

COMPARING SIGNATURE-TRAINED RISK SCORES – LIKELIHOOD RATIOS:

We compared the ability of trained signatures to predict DMFS10 using the likelihood ratio χ^2($LR\chi^2$) based on the Cox models as a measure of the overall prognostic information provided by each model (Supplementary Table 9). For this analysis we did not report results for binary categorization of either Prosigna-trained or Oncotype-Trained results. We illustrated the performance of each "trained" signature using Kaplin-Meier survival curves and estimated Hazard ratios as described above (See Supplementary Figure 16). We calculated the change in $LR\chi^2$ values ($\Delta LR\chi^2$) between the bivariate and univariate signature models to assess prognostic improvement of reclassification with a second signature *versus* the single signature using existing trinary cut points as outlined above(Supplementary Table 9).

In contrast to the main analysis, restricted to ER+ve/HER2-ve cases, in HR+/HER2any cases the $LR\chi^2$ values for individual tests were broadly similar (160.4-172.8) indicating approximately equivalent prognostic value across all 3 tests when HER2+ve cases were included. However, consistent with the main analysis, combining Prosigna-trained and Oncotype-trained results giave

the greatest overall LRχ^2 (Supplementary Table 9). Whilst the increase in LRχ^2 reported by combining Prosigna-trained and Oncotype-trained results compared with either test alone was comparable, inclusion of Mammaprint-trained results with either of the other two tests produced a more modest improvement in LRχ^2(Supplementary Table 9).

ANALYSIS OF TEST PERFORMANCE BY OUTCOME IN RECLASSIFIED PATIENTS:

We analyzed agreement between tests by investigating the extent to which re-classifying results for individual patients by performing tests in sequence affected predicted outcome. *i.e.*, we analysed the effects of performing a Prosigna-trained test on tumours previously classified as intermediate risk by the Oncotype-trained test.

"ONCOTYPE-DX-TRAINED" RESULTS: ALL HR+VE CASES (HER2+VE AND HER2-VE)

Of 3811 cases (HR+ve/HER2any) with results for "Oncotype DX-trained" risk classification results available; 44.0% were low risk (88.0% DMFS10, 95% C.I. 86.0-89.7%), 33.8% were intermediate risk (79.1% DMFS10, 95% C.I. 76.5-81.4%) and 22.2% high risk (66.6% DMFS10, 95% C.I. 63.0-69.9%) (Supplementary Table 10; Supplementary Figure 16A).

a) **"Oncotype-DX-trained" results stratified by "Prosigna-trained" results:**

When "Oncotype-DX-trained" results were further stratified by "Prosigna-trained" results a significant proportion (46.3%) of cases remained in the same risk category (Supplementary Table 11). However 294 "Oncotype-trained" low risk cases (17.5%) were classified as high risk by "Prosigna-trained" results with a DMFS10 of 75.6% (95% C.I. 68.7-81.2%; p<0.001; Supplementary Table 10; Supplementary Figure 17A). In the "Oncotype -trained" intermediate risk group 186 cases (14.4%) were classified as "Prosigna-trained" low risk with a DMFS10 of 90.9% (95% C.I. 84.8-94.6%; p<0.001; Supplementary Table 10; Supplementary Figure 17B). A further 678 (52.2%) of "Oncotype -trained" intermediate risk patients were classified as "Prosigna-

trained" high risk with DMFS10 of 74.4% (95% C.I. 70.5-77.8%; p<0.001). However, no significant stratification of "Oncotype-DX-trained" high risk cases was observed using "Prosigna-trained" subgroups (Supplementary Table 10; Supplementary Figure 17C).

Overall 186 "Oncotype -trained" intermediate risk cases (4.9% of all cases) were "Prosigna-trained" low risk with DMFS10 >90%. In contrast 972 "Oncotype -trained" low risk and intermediate risk cases (25.5% of all cases) were classified as "Prosigna-trained" high risk with DMFS10 of 74.4-75.6% (Supplementary Table 10).

b) **"Oncotype-DX-trained" results stratified by "MammaPrint-Trained" results:**

Comparing only cases classified as either high or low risk between these tests shows the majority of results (87.1%) remained in the same risk category (Supplementary Table 11); whilst the majority of "Oncotype-DX-trained" low cases were also "MammaPrint-trained" low risk (92.2%). Only 131 "Oncotype -trained" low risk cases (7.8%) were classified as high risk by "MammaPrint-trained" results with a DMFS10 of 72.7% (95% C.I. 62.0-80.9%; Supplementary Table 10; Supplementary Figure 17). In contrast 89 "Oncotype -trained" high risk cases were classified as low risk by "MammaPrint-trained" results, but these cases whilst of marginally lower risk than those classified as high risk by both tests exhibited a DMFS10 of 70.1% (95% C.I. 69.7-77.7%) still reflected a high risk population. Finally of the 1291 "Oncotype -trained" intermediate risk cases 586 (45.4%) were classified as "MammaPrint-trained" high risk with DMFS10 of 74.0% (95% C.I. 69.7-77.7%; Supplementary Table 10; Supplementary Figure 17B).

Across "Oncotype -trained" low and intermediate risk cases "MammaPrint-trained" results identified 18.8% of all cases as high risk and these cases exhibited DMFS10 <80% (Supplementary Table 10; Supplementary Figure 17A & 2C).

"PROSIGNA-TRAINED" RESULTS HR+VE, HER2+VE & -VE CASES:

Of 3811 HR+ve, HER2+ve and -ve breast cancers with results for "Prosigna-trained" risk classification results available 22.8% were low risk (91.4% DMFS10, 95% C.I. 89.0-93.3%), 33.9% were intermediate risk (84.8% DMFS10, 95% C.I. 82.4-86.9%) and 43.2% high risk (70.8% DMFS10, 95% C.I. 68.2-73.1%; Supplementary Table 12; Supplementary Figure 16B).

"Prosigna-trained" results stratified by "Oncotype-DX-trained" results:

In cases classified as "Prosigna-trained" low risk there were no significant differences in outcome between "Oncotype -trained" low or intermediate risk patients (Supplementary Table 12; Supplementary Figure 18A); Only 14 "Prosigna-trained" low risk cases (1.6%) were classified as "Oncotype -trained" high risk but experienced DMFS10 of 59.3% (95% C.I. 27.5-81.0%; $p<0.001$; Supplementary Table 12). "Prosigna-trained" high, "Oncotype-trained" high risk cases experienced significantly poorer outcome (DMFS10 64.9% 95% C.I. 60.9-68.6%) than "Prosigna-trained" high risk cases classified as either low or intermediate risk by "Oncotype -trained" results. However, in these cases those cases with "Prosigna-trained" high risk but "Oncotype -trained" low risk or intermediate risk still experienced DMFS10 of <80% (Supplementary Table 12; Supplementary Figure 18C). Of the 1293 "Prosigna-trained" intermediate risk cases, 716 (55.4%) were classified as low risk by "Oncotype -trained" but exhibited DMFS10 <90% (DMFS10 of 88.6% (95% C.I. 85.6-91.1%). A further 154 cases (11.9%) were classified as "Oncotype-trained" high risk with DMFS10 of 74.5% (95% C.I. 66.1-81.1%; Supplementary Table 12; Supplementary Figure 18B).

"Prosigna-trained" stratified by "MammaPrint-Trained" results:

Here when comparing just the high and low risk categories between these tests the majority of results (80.5%) remained in the same risk category (Supplementary Table 11). However whilst, 28.6% of "Prosigna-trained" high risk cases were classified as low risk by "MammaPrint trained"

results these cases still experienced DMFS10 <80% (78.0% 95% C.I. 73.2-82.0%). Amongst "Prosigna-trained" low risk cases (N=869) 2.0% were classified as high risk (N=17) by "MammaPrint-trained" results (DMFS10 67.0% 95% C.I. 37.7-84.9%, p<0.001; Supplementary Table 12; Supplementary Figure 18A). "Prosigna-trained" intermediate patients showed significantly different outcomes when further stratified by "MammaPrint-trained" classification (Supplementary Table 12; Supplementary Figure 18B); 21.3% were identified as "MammaPrint-trained" high risk with DMFS10 of 78.7% (95% CI 72.8-83.4%). Again whilst "MammaPrint-trained" low risk cases within the "Prosigna-trained" intermediate risk group experienced higher 10 DMFS (86.4% 95% CI 83.8-88.7%) this did not reach a level sufficient to classify these cases as "clinically-low risk" using a 90% 10 year estimated DMFS as a cut point (Supplementary Figure 18C; Supplementary Table 12).

"MAMMAPRINT-TRAINED" RESULTS:

Of 3811 HR+ve, HER2+ve or -ve breast cancers with results for "MammaPrint-Trained" risk classification results available 61.4% were low risk (86.7% DMFS10, 95% C.I. 85.0-88.2%) and 38.6% high risk (70.0% DMFS10, 95% C.I. 67.3-72.4 %) (Supplementary Table 13; Supplementary Figure 16).

"MammaPrint-trained" results stratified by "Oncotype-DX-trained" results:

Of the 2342 "MammaPrint-trained" low risk cases, 1548 (66.1%) were classified as low risk by "Oncotype -trained" results with DMFS10 of 89.2% (95% C.I. 87.2-90.9%), 705 (30.1%) were classified as "Oncotype-trained" intermediate risk with DMFS10 of 83.4% (95% CI 80.0-86.2%) and 89 (3.8%) as "Oncotype -trained" high risk with DMFS10 of 70.1% (95% C.I. 57.6-79.5%, p<0.001; Supplementary Table 13, Supplementary Figure 19A). Despite significant differences in outcome across cases classified as "MammaPrint-trained" high risk when further stratified by

"Oncotype -trained" low, intermediate or high risk patients no sub group exhibited DMFS >75% (Supplementary Table 13; Supplementary Figure 19B).

"MammaPrint-Trained" results stratified by "Prosigna-trained" results:

In "MammaPrint-trained" low risk cases, "Prosigna-trained" risk scores showed a significant added stratification of DMFS10; 20.1% of cases were identified as high risk (DMFS10 78.0% 95% C.I. 73.2-82.0%; p<0.001) and 43.4% of cases as intermediate risk (DMFS10 86.4% 95% C.I. 83.8-88.7%; Supplementary Table 13; Supplementary Figure 19A) vs DMFS10 of 91.9% for cases low risk by both tests. However, amongst cases classified as "MammaPrint-trained" high risk, despite a significant p value for trend, no marked stratification was achieved when "Prosigna-trained" results were used to further stratify these cases (Supplementary Table 13; Supplementary Figure 19B).

ALL PATIENTS (HR+VE/HER2 ANY) SUB-GROUP ANALYSES:

I) "ONCOTYPE -TRAINED" RESULTS STRATIFIED BY "PROSIGNA-TRAINED" RESULTS: SUB-GROUP ANALYSES:

a) Patients with HR+ve/HER +ve and –ve, node negative disease treated without adjuvant chemotherapy

The performance of "Oncotype-trained" results in this subgroup are represented in Supplementary Figure 20A. DMFS10 for "Oncotype-trained" low, intermediate and high risk groups were 92.6%, 86.4% and 73.0% respectively (Supplementary Table 10).

In node negative cases treated without chemotherapy when "Oncotype-trained" results were further stratified by "Prosigna-trained" results 46.2% of cases remained in the same risk category (Supplementary Table 14). Results for "Oncotype-trained" low risk cases classified as "Prosigna-trained" low or intermediate risk were not significantly different (DMFS10 96.9% vs 92.7%; p

=0.20), however 99 “Oncotype-trained” low risk cases (20.6%) were classified as high risk by “Prosigna-trained” results with DMFS10 of 84.6% (95% C.I. 71.6-92.0%, p=0.01; ; Supplementary Table 10, Supplementary Figure 21). In “Oncotype-trained” intermediate risk cases there was no further statistically significant stratification by “Prosigna-trained” scores. Finally, in “Oncotype -trained” high risk cases only 6 were classified as low risk by “Prosigna-trained” risk scores, but a small group of “Prosigna-trained” intermediate risk cases exhibited DMAS10 of 92.3% (95% C.I. 77.5-97.5; p=0.01; Supplementary Figure 21; Supplementary Table 10).

b) Patients with HR+ve/HER+ve & -ve node positive disease treated without adjuvant chemotherapy:

The performance of “Oncotype-trained” results in this subgroup are represented in Supplementary Figure 22. DMFS10 for “Oncotype-trained” low, intermediate and high risk groups were 86.2%, 77.5% and 56.8% respectively (Supplementary Table 10; Supplementary Figure 23).

Stratifying “Oncotype-trained” results by “Prosigna-trained” results 628 cases (45.3%) remained in the same risk category (Supplementary Table 15). Here 118 “Oncotype-trained” low risk cases (16.6%) were classified as high risk by “Prosigna-trained” results (DMFS10 69.3% 95% C.I. 57.2-78.5%; p<0.001; Supplementary Table 10; Supplementary Figure 23). Amongst cases classified as intermediate risk by “Oncotype-trained” results 57 (13.1%) were classified as low risk (DMFS10 92.9% (95% C.I. 82.1-97.3%: and 223 (51.4%) as high risk (DMFS10 70.4 (95% C.I. 63.2-76.4%; p=0.005; Supplementary Table 10; Supplementary Figure 23) by “Prosigna-trained” results. Amongst cases with high risk “Oncotype-trained” scores no significant differences in outcome were observed, with only 1 case classified as “Prosigna-trained” low risk and 37 (15.4%)

cases classified as intermediate risk using "Prosigna-trained" results (Supplementary Table 10; Supplementary Figure 23).

c) **Patients with HR+ve/HER+ve & -ve disease treated with chemotherapy (Node positive or negative).**

Results in this subgroup showed DMFS10 for "Oncotype-trained" low, intermediate and high risk groups were 85.9%, 74.3% and 68.5% respectively (Supplementary Table 10; Supplementary Figure 24).

When "Oncotype-trained" results were further stratified by "Prosigna-trained" results 667 cases (47.7%) remained in the same risk category (Supplementary Table 16). No significant differences in outcome were observed with "Oncotype-trained" high risk cases further stratified by "Prosigna-trained" results. However 77 "Oncotype-trained" low risk cases (15.8%) were classified as High risk by "Prosigna-trained" results (DMFS10 73.1%; 95.% C.I. 58.9-83.1; p<0.001) and a further 39.7% of "Oncotype-trained" low risk cases were "Prosigna-trained intermediate risk although outcome in this group was not significantly worse that cases low risk by both results (DMFS10 84.2% vs 91.9% respectively; p=0.06; Supplementary Table 10; Supplementary Figure 25). In "Oncotype -trained" intermediate risk cases 83 cases (18.1%) classified as "Prosigna-trained" low risk demonstrated DMFS10 of 87.5%, a further 227 cases (49.4) of cases classified as Prosigna-trained high risk exhibited DMFS10 of 68.0% (p=0.004; Supplementary Table 10; Supplementary Figure 25).

II) "ONCOTYPE-DX-TRAINED" RESULTS STRATIFIED BY "MAMMAPRINT-TRAINED" RESULTS: SUB-GROUP ANALYSES

a) **Patients with HR+ve/HER+ve or -ve node negative disease treated without adjuvant chemotherapy:**

In patients with “Oncotype-trained” low risk results, 55 (11.5%) were “Mammaprint-trained” high risk with DMFS10 of 82.6%, significantly lower than the 96.7% DMFS10 observed for cases low risk by both results (p=0.009; Supplementary Table 10; Supplementary Figure 21). Amongst “ONcotype-trained” intermediate cases, those classified as “Mammaprint-trained” low risk exhibited DMFS10 of 92.0% versus 81.0% (p=0.004) for those with “Mammaprint-trained” high risk results (Supplementary Table 10; Supplementary Figure 21). Amongst “Oncotype-trained” high risk cases 28 were “Mammaprint-trained” low risk with DMFS10 92.7% (Supplementary Table 10; Supplementary Figure 21).

b) Patients with HR+ve/HER+ve or -ve node positive disease treated without adjuvant chemotherapy:

In patients with node-positive disease treated without chemotherapy 47 “Oncotype -trained” low risk cases were classified as high risk by “MammaPrint-trained” scores (6.6%), and these patients experienced DMFS10 of 66.7% (95% C.I. 47.8-80.2%; Supplementary Table 10; Supplementary Figure 23). Similarly in “Oncotype-trained” intermediate risk patients 181 (41.5%) were classified as “MammaPrint-trained” high risk with 10 DMFS of 67.8% (95% C.I. 59.8-78.0; p<0.001; Supplementary Table 10; Supplementary Figure 23). However, no statistically significant stratification of “Oncotype-trained” high risk cases was observed (Supplementary Figure 21F). In node positive cases treated without chemotherapy “MammaPrint-trained” high risk scores identified 228 “Oncotype -trained” low or intermediate risk cases as high risk with DMFS10 of <70%.

c) Patients with HR+ve/HER+ve or -ve disease treated with chemotherapy (Node positive or negative).

Only 29 "Oncotype-trained" low risk cases (6.0%) were classified as high risk by "MammaPrint-trained" results with DMFS10 of 64.2% (95% C.I. 37.6-81.8%, p=0.003). Amongst "Oncotype - trained" intermediate and high risk cases no significant stratification by "MammaPrint-trained" scores was observed (Supplementary Table 10; Supplementary Figure 25).

III) "Prosigna-trained" results stratified by "Oncotype-Dx-trained" results: sub-group analyses:

a) Patients with HR+ve/HER+ve or -ve node negative disease treated without adjuvant chemotherapy:

The overall performance of the "Prosigna-trained" scores for this sub-group are represented in Supplementary Figure 20, DMFS10 was 95.0%, 90.9% and 78.6% for low, intermediate and high risk cases respectively. Within "Prosigna-trained" low risk cases, no significant differences between intermediate and low risk results from "Oncotype-trained" results was observed and only 6 cases (2.6%) were classified as high risk by "Oncotype-trained" results with DMFS10 of 50.0% (95% C.I. 11.1-80.4%; p<0.001: Supplementary Figure 26; Supplementary Table 12). In this sub-group of patients "Prosigna-trained" intermediate risk patients showed no statistically significant sub-stratification using "Oncotype -trained" low, intermediate or high risk scores (Supplementary Figure 26, Supplementary Table 12). Amongst "Prosigna-trained" high risk cases those identified as low risk (17.9%) and intermediate risk (40.6%) by "Oncotype -trained" results experienced 84.6% and 84.4% DMFS10 respectively (Supplementary Table 10; Supplementary Figure 26).

b) Patients with HR+ve/HER+ve or -ve node positive disease treated without adjuvant chemotherapy:

Cases with "Prosigna-trained" scores for this sub-group was associated with DMFS10 rates of 90.1%, 85.8% and 64% for low, intermediate and high risk groups respectively (Supplementary

Table 12, Supplementary Figure 22). Amongst node positive patients treated without chemotherapy only 1 “Prosigna-trained” low risk case was high risk by “Oncotype -trained” results, no significant difference in survival was observed between “Oncotype-trained” low and intermediate risk cases (Supplementary Table 12; Supplementary Figure 27). Of “Prosigna-trained” intermediate risk cases: 62.6% were “Oncotype-trained” low risk (DFMS10 88.8%, 95% C.I. 84.2-92.2) and 7.2% high risk (DMFS10 72.7% 95% C.I. 53.9-84.9%; p=0.002; Supplementary Table 12; Supplementary Figure 27). Amongst “Prosigna-trained” high risk cases a significantly higher risk was seen for cases classified as high risk by both results (DMFS10 53.7% 95% C.I. 46.1-60.8%; Supplementary Table 12; Supplementary Figure 27) however, no group of “Prosigna-trained” high risk cases, irrespective of their “Oncotype-trained” category, exhibited DMFS10 >75% (Supplementary Figure 27).

c) Patients with HR+ve/HER+ve or -ve disease treated with adjuvant chemotherapy (node –ve and node positive):

Overall “Prosigna-trained” stratification of this sub-group are represented showed DMFS10 of 90.1%, 78.3% and 69.4% for low, intermediate and high risk cases (Supplementary Table 12; Supplementary Figure 24). In patients who received chemotherapy, irrespective of nodal status, no further statistically significant stratification was observed using the “Oncotype-trained” risk scores following classification of these cases as either low or high risk by “Prosigna-trained” risk scores (Supplementary Figure 28; Supplementary Table 12). Only 7 cases (2.3%) “Prosigna-trained” low risk cases with “Oncotype-trained” high risk scores experienced DMFS10 (66.7% 95% C.I. 19.5-90.4%; p=0.01; Supplementary Table 12, Supplementary Figure 28). Apart from this statistically significant stratification was confined to those cases classified as intermediate risk by “Prosigna-trained” results. Amongst these cases 193 cases were classified as “Oncotype-

trained" low risk (46.7%) with DMFS10 of 84.2% (95% C.I. 77.1-89.2%), 147 as intermediate risk (35.6%, DFMS10 77.2%, 95% C.I. 69.0-83.5) and 73 cases as "Oncotype-trained" high risk (17.7%) with DMFS10 of 64.9% (95% C.I. 51.5-75.5%; p,0.001; Supplementary Figure 28; Supplementary Table 12).

IV) "Prosigna-trained" stratified by "MammaPrint-Trained" results: Sub-group analyses:

a) Patients with HR+ve/HER+ve or -ve node negative disease treated without adjuvant chemotherapy:

For cases of node-negative disease not receiving chemotherapy only 6 cases with "MammaPrint-trained" high scores were observed in the "Prosigna-trained" low risk group (Supplementary Figure 26), with DMFS10 of 67.0%. For both moderate and high risk "Prosigna-trained" results a group of low risk by "MammaPrint-trained" results (422 cases) exhibited DMFS10 of 93.2% & 89.6% respectively (95% C.I. 88.5-96.0% & 81.7-94.2%; Supplementary Figure 26; Supplementary Table 12).

b) Patients with HR+ve/HER+ve or -ve node positive disease treated without adjuvant chemotherapy:

No additional stratification was observed in "Prosigna-trained" low and intermediate risk cases when they were further split by "MammaPrint-trained" results (Supplementary Figure 27). In "Prosigna-trained" high risk cases, "MammaPrint-trained" results split cases into two groups with DMFS10 of 75.7% and 58.5% respectively (Supplementary Figure 27; Supplementary Table 12).

c) Patients with HR+ve/HER+ve or-ve disease treated with chemotherapy (Node positive or negative).

Amongst "Prosigna-trained" low risk cases 8 (2.6%) were categorized as "MammaPrint-trained" high risk (Supplementary Figure 28; DMFS10 53.6%). Amongst "Prosigna-trained" intermediate risk cases 73.8% were "MammaPrint-trained" low risk (Supplementary Figure 28; DMFS10 80.5%) and 26.2% high risk (DMFS10 72.4%; p=0.02). For patients in the "Prosigna-trained" high risk category there was no evidence that sub-stratifying scores by "MammaPrint-trained" scores identified any significant differences (Supplementary Figure 28; Supplementary Table 12).

V) "MAMMAPRINT-TRAINED" RESULTS STRATIFIED BY "ONCOTYPE -TRAINED" RESULTS: SUB-GROUP ANALYSES:

a) **Patients with HR+ve/HER+ve or -ve node negative disease treated without adjuvant chemotherapy:**

Results for "MammaPrint-trained" scores are shown in Supplementary Figure 20. Within this sub-group of patients those with "MammaPrint-trained" low risk results showed no statistically significant sub-stratification using "Oncotype-trained" low, intermediate or high risk scores (Supplementary Figure 29). In "Mammaprint-trained" high risk cases, DMFS10 was 82.6%, 81.0% and 70.9% (p=0.013) for "Oncotype-trained" low, intermediate and high risk cases respectively (Supplementary Figure 29; Supplementary Table 13).

b) **Patients with HR+ve/HER+ve or -ve node positive disease treated without adjuvant chemotherapy:**

Results for "MammaPrint-trained" scores are shown in Supplementary Figure 22. Amongst node positive patients treated without chemotherapy 31 cases with "MammaPrint-trained" low and "Oncotype -trained" high risk scores (3.3%) exhibited statistically significant reductions in outcome when compared with cases scored as low risk by both test results (p<0.001; Supplementary Figure 30) with DMFS10 of 57.2% (95% C.I. 35.3-74.0%). No differences in

outcome were observed between low or intermediate risk "Oncotype -trained" results in this subgroup (Supplementary Figure 30). Results for cases classified as "MammaPrint-trained" high risk were not further stratified by "Oncotype-trained" risk categories into clinically meaningful groups, all subgroups experienced DMFS10 <70% (Appndix Figure 15; Supplementary Table 13).

c) Patients with HR+ve/HER+ve or -ve disease treated with adjuvant chemotherapy (node –ve and node positive):

Results for "MammaPrint-trained" scores are shown in Supplementary Figure 24. Further stratification using "Oncotype-trained" risk scores was confined to cases classified as low risk by "MammaPrint-trained" results (Supplementary Figure 31). The majority (61.8%) of these cases were classified as "Oncotype-trained" low risk with DMFS10 of 87.2% (95% C.I. 83.2-90.3%), 34.1%% were classified as "Oncotype-trained" intermediate risk with DMFS10 of 75.9% (95% C.I. 69.6-81.1%), with 4.1% of cases were classified as "Oncotype-trained" high risk with DMFS10 of 62.8% (95% C.I. 39.8-79.1%; p<0.001 Supplementary Figure 31; Supplementary Table 13).

V) "MammaPrint-Trained" results stratified by "Prosigna-trained" results: Sub-group analyses

a) Patients with HR+ve/HER+ve or -ve node negative disease treated without adjuvant chemotherapy:

No statistically significant stratification of "MammaPrint-trained" low or high risk cases was observed when results were stratified by "Prosigna-trained" sub-groups (Supplementary Figure 29; Supplementary Table 13).

b) Patients with HR+ve/HER+ve or -ve node positive disease treated without adjuvant chemotherapy:

In “MammaPrint-trained” low risk cases 19.2% were classified as high risk by “Prosigna-trained” results (DMFS10 75.7% 95% C.I.67.3-82.2%; <0.001, Supplementary Figure 30). A further 46.5% were classified intermediate risk by “Prosigna-trained” results (DMFS10 86.5% 95% C.I. 82.3-89.8%), however there was no statistically significant difference in outcome between these cases and those regarded as low risk by both test results. In “MammaPrint-trained” high risk cases a small group of “Prosigna-trained” intermediate risk score cases (16.7%) exhibited improved outcome when compared to cases classified as high risk by both scores (DMFS10 81.4% Supplementary Figure 30; Supplementary Table 13). No events were observed in the 3 “Prosigna-trained” low risk “MammaPrint-trained” high risk cases.

c) **Patients with HR+ve/HER+ve or -ve disease treated with chemotherapy (Node positive or negative).**

Across patients in “MammaPrint-trained” low risk categories “Prosigna-trained” results identified cases with intermediate risk (41.3%) and high risk (18.3%) with DMFS10 of 80.5 & 68.1% respectively (p=0.002 and p<0.001 respecitvely; Supplementary Figure 31). There was no evidence that sub-stratifying “MammaPrint-trained” high scores by “Prosigna-trained” scores identified significant differences (Supplementary Figure 31; Supplementary Table 13).

Discussion:

The key findings of this paper are reviewed in the main discussion, herein we are focused on particular nuances which may apply to sub-groups analyzed as part of the data including HER2+ve cases presented above. As pointed out in many settings, sub-group analyses must be interpreted with caution, due to reduce power, multiple testing etc. Here, whilst HER2+ve cases are now included, the majority of cases (87%) were HER2-ve and thus these results are strongly influenced by the cases already reported in the main manuscript. As a result, we recognize that the

interpretation of these sub-group analyses is limited and strongly recommend readers review the data presented and draw their own conclusions.

Overall prognostic impact of different tests across subgroups:

Whilst evidence focusing solely on LRχ^2 results suggests a potential difference between this analysis and that reported in the main manuscript and restricted to ER+ve/HER2-ve cases, subsequent analysis of the outcomes in cases with discrepant results between tests do not appear to bear this difference out. In contrast to the main analysis, there appeared to be similar prognostic value, when measured by LRχ^2 values, garnered from all three test approaches (Supplementary Table 9). Consistent with the main analysis combining Oncotype-trained and Prosigna-trained results produced the optimal LRχ^2 metric whilst combinations of tests including Mammaprint-trained results appeared less effective. The differences observed here between the ER+/HER-ve and HR+/HER2any populations may represent a different weighting for HER2 related modules between the different prognostic tests, consistent with a recent report by Dowsett et al (add ref). This different would be consistent with the different approaches taken, and in particular populations used, to train the individual tests which appear to have resulted in different weights being given to HER2 overexpression/signaling modules and which would therefore differentially impact results from populations excluding or including HER2+ve cases.

Consistent with the main analyses reported, each of the tests studied provided clear separation of patients into low, intermediate (where applicable) and high risk strata. As expected and consistent with previous data, there are differences in DMFS10 by nodal status. For example in node negative low risk cases treated without chemotherapy DMFS10 ranges from 92.6-95.0 (dependent on test; Supplementary Tables 10, 12, 13). For node positive cases, also treated without chemotherapy, the range is 85.7-90.1 (Supplementary Tables 10, 12, 13). These results, despite the inclusion of

HER2+ve cases, treated within targeted HER2 therapies, are within 1-2% of those observed in the HER2-ve population reported in the main manuscript (Tables 2-4; See Supplementary Table 17 for direct comparison of DMFS10 results between groups). For high risk cases, the difference is more stark with DMFS10 for node negative cases ranging from 73.0-78.6% and for node positive cases from 56.8-64.0%, again in cases treated without chemotherapy (Supplementary Tables 10, 12, 13). Again whilst these results are marginally lower than those seen in the HER2-ve population the inclusion of HER2+ve cases does not dramatically alter the overall outcomes in any prognostic grouping defined by the tests applied here. Therefore, it is highly likely that differences between risk estimates for individual test comparisons when HER2+ve cases are included or excluded are only moderately different and that similar conclusions can be drawn from analyses, excluding or including these cases. Nonetheless these analyses are included here for completeness and to allow clinicians to review the data and draw their own conclusions in the context of current treatment approaches.

ONCOTYPE-TRAINED RESULTS STRATIFIED BY PROSIGNA-TRAINED AND MAMMAPRINT-TRAINED RESULTS:

Node negative patients treated without chemotherapy:

Results across all risk groups in patients with node negative disease treated without adjuvant chemotherapy were essentially consistent between the main analyses for ER+ve/HER2-ve cases and this HR+ve/HER2any group (Figure 5 & Supplementary Figure 21; Table 2 & Supplementary Table 10).

Node positive patients treated without chemotherapy:

Results in these cases were entirely consistent between ER+/HER2-ve cases (Supplementary Figure 9) and those observed in the HR+ve/HER2any group presented above (Supplementary Table 10; Supplementary Figure 23) for all risk groups and both Prosigna-trained and Mammaprint-trained results.

Patients treated with chemotherapy:

In patients HR+ve/HER2any cases treated with chemotherapy (a mixture of both node positive and node negative cases; Supplementary Figure 29) were entirely consistent with a similar analyses for ER+ve/HER2-ve (Supplementary Figure 11) for both Oncotype-trained and Mammaprint-trained results stratified by Prosigna-trained results.

Summary

Overall for results observed when Oncotype-trained results were stratified by Prosigna-trained or MammaPrint-trained results were compared in subgroup analyses versus the main analyses reported in the main paper we saw no evidence to suggest the overarching conclusions were different in this analysis including HER2+ve cases.

PROSIGNA TRAINED RESULTS STRATIFIED BY ONCOTYPE-TRAINED AND MAMMAPRINT-TRAINED RESULTS:

Node negative patients treated without chemotherapy:

In contrast to the ER+ve/HER2-ve analysis (Figure 6), 6 Prosigna-trained low risk cases were identified as high risk by Oncotype-trained results (Supplementary Table 12, Supplementary Figure 22) in ER+ve HER2 any cases. However results for Prosigna-trained intermediate risk and high risk cases were consistent between the ER+ve/HER2-ve group and the current analysis (Supplementary Table 12; Supplementary Figure 22).

Also using MammaPrint-trained stratification after Prosigna-trained classification, 6 low risk Prosigna-trained low risk cases were classified as high risk by Mammaprint-trained results with DMFS10 of 67.0% (Supplementary Figure 22). In intermediate and high risk cases MammaPrint-trained low risk cases exhibited 93% and 89.6% DMFS10 respectively (Supplementary Figure 22, Supplementary Table 12) suggesting in node negative cases some stratification may occur with MammaPrint-trained results in these cases consistent with the results of the main ER+ve/HER2-ve analysis (Figure 6).

Node positive patients treated without chemotherapy:

Results in Prosigna-trained risk groups across all risk strata, were consistent when further stratified by either Oncotype-trained or MammaPrint trained results between ER+/HER2-ve (Supplementary Figure 12) or HR+/HER2any (Supplementary Figure 27; Supplementary Table 12) cases.

Patients treated with chemotherapy:

Again, across all risk strata, when Prosigna-trained results were further stratified by Oncotype-trained or Mammaprint-trained results, there were no differences when comparing the HR+ve/HER2any results (Supplementary Figure 30) with those from the ER+ve/HER2-ve group (Supplementary Figure 12).

Summary

The only variation between these analyses of HR+ve/HER2any cases, when compared with the ER+ve/HER2-ve subgroup was the identification of a small (n=6) group of cases identified as high risk by either Oncotype-trained or Mammaprint-trained results amongst cases classified as low risk by Prosigna-trained results (Supplementary Figure 22). All other results were consistent with

the main analyses reported. Therefore overall we concluded that results of subgroup analyses for Prosigna-trained results stratified by other tests reflect the main reported analyses.

MammaPrint trained results stratified by Oncotype-trained and Prosigna-Trained results:

Node negative patients treated without chemotherapy:

Consistent with the results of the main analysis (Figure 7), for patients with node-negative disease treated without chemotherapy, there was no statistically significant stratification of MammaPrint-trained low or high risk cases when further stratified by either Oncotype-trained or Prosigna-trained (Supplementary Table 13; Supplementary Figure 29) results.

Node positive patients treated without chemotherapy:

Results in the HR+ve/HER2any sub-group of cases treated without chemotherapy (Supplementary Figure 27) were entirely consistent with both the main analysis of ER+ve/HER2-ve cases (Figure 4) and the sub-group analysis of ER+ve/HER2-ve cases treated without chemotherapy (Supplementary Figure 14).

Patients treated with chemotherapy:

Consistent with the results of the main analysis (Figure 4) in both ER+ve/HER2-ve (Supplementary Figure 15) and HR+ve/HER2any (Supplementary Figure 31) groups for cases with MammaPrint trained low risk results both Oncotype-trained results (Supplementary Figure 12A, 28A) and Prosigna-trained results (Supplementary Figure 12C, 28C) identified high risk subgroups. For MammaPrint-trained high risk groups, whilst statistically significant, stratification was achieved with both Oncotype-trained and Prosigna-trained results in the main analysis Figure 4), no statistically significant stratification of Mammaprint-trained high risk patients was observed

in the chemotherapy treated sub-group in either the ER+/HER2-ve (Supplementary Figure 15) or HR+ve/HER2any (Supplementary Figure 31) sub-groups.

Summary:

No differences were observed when Mammaprint-trained results were further stratified by either Prosigna-trained or Oncotype-trained results in this analysis of HR+ve/HER2any cases when compared to either the main analysis or sub-group analyses in the ER+ve/HER2-ve cases reported in the main paper and supplementary data.

Conclusion:

In the main analysis presented in the body of this paper and the supplementary data we restricted our reporting to patients with ER+ve/HER2-ve disease. This reflected the fact that, at the time when the TEAM trial was recruiting adjuvant therapies targeted HER2+ve cases were not available. This posed the risk that results in the HER2+ve population might not be representative of the current patient population. However, we also noted that none of the signatures in this comparison were developed using patients treated with HER2-targeted therapies. Despite marked differences in the relative LRχ^2 values obtained in the analyses focused on HER2-ve cases and the results reported here including HER2+ve cases, there was minimal impact on downstream analysis focused on outcome in cases with different classification between tests. In that context, in this analysis of patients, which included those with HER2-ve and HER2+ve cases, we observed no marked differences in results to those seen in the ER+ve/HER2-ve population. This may be due in part to overlapping cases between the two groups. However, this additional analysis supports the interpretation that observations and conclusions from ER+ve/HER2-ve cases, across all tests and sub-groups analysed, are applicable to the population including HER2+ve cases.

References;

1. Bartlett JM, Brookes CL, Piper T, et al: Do type 1 receptor tyrosine kinases inform treatment choice? A prospectively planned analysis of the TEAM trial. Br. J Cancer 109:2453-2461, 2013
2. van de Velde CJH, Rea D, Seynaeve C, et al: Adjuvant tamoxifen and exemestane in early breast cancer (TEAM): a randomised phase 3 trial. Lancet 377:321-331, 2011
3. Bartlett JMS, Brookes CL, Robson T, et al: Estrogen Receptor and Progesterone Receptor As Predictive Biomarkers of Response to Endocrine Therapy: A Prospectively Powered Pathology Study in the Tamoxifen and Exemestane Adjuvant Multinational Trial. Journal of Clinical Oncology 29:1531-1538, 2011
4. Bayani J, Yao CQ, Quintayo MA, et al: Molecular stratification of early breast cancer identifies drug targets to drive stratified medicine. npj Breast Cancer 3:3, 2017
5. Bartlett JM, Bayani J, Marshall A, et al: Comparing Breast Cancer Multiparameter Tests in the OPTIMA Prelim Trial: No Test Is More Equal Than the Others. J. Natl. Cancer Inst 108:djw050, 2016
6. Bartlett JMS, Bayani J, Kornaga EN, et al: Computational approaches to support comparative analysis of multiparametric tests: Modelling versus Training. PloS one 15:e0238593-e0238593, 2020
7. Paik S, Shak S, Tang G, et al: A multigene assay to predict recurrence of tamoxifen-treated, node-negative breast cancer. N. Engl. J. Med 351:2817-2826, 2004
8. Paik S, Tang G, Shak S, et al: Gene Expression and Benefit of Chemotherapy in Women With Node-Negative, Estrogen Receptor-Positive Breast Cancer. Journal of Clinical Oncology 24:3726-3734, 2006
9. Chia SK, Bramwell VH, Tu D, et al: A 50-Gene Intrinsic Subtype Classifier for Prognosis and Prediction of Benefit from Adjuvant Tamoxifen. Clinical Cancer Research 18:4465-4472, 2012
10. Nielsen TO, Parker JS, Leung S, et al: A Comparison of PAM50 Intrinsic Subtyping with Immunohistochemistry and Clinical Prognostic Factors in Tamoxifen-Treated Estrogen Receptor-Positive Breast Cancer. Clinical Cancer Research 16:5222-5232, 2010
11. van't Veer LJ, Dai HY, van de Vijver MJ, et al: Gene expression profiling predicts clinical outcome of breast cancer. Nature 415:530-536, 2002
12. Bayani J, Yao CQ, Quintayo MA, et al: Molecular stratification of early breast cancer identifies drug targets to drive stratified medicine. npj Breast Cancer 3:3, 2017
13. Sestak I, Buus R, Cuzick J, et al: Comparison of the Performance of 6 Prognostic Signatures for Estrogen Receptor–Positive Breast Cancer: A Secondary Analysis of a Randomized Clinical TrialPrognostic Signatures for Estrogen Receptor–Positive Breast CancerPrognostic Signatures for Estrogen Receptor–Positive Breast Cancer. JAMA Oncology 4:545-553, 2018
14. Sestak I, Ferree S, Shemesh I, et al: Abstract P5-06-05: Discordant classification and outcomes between Prosigna and Oncotype Dx Recurrence Score for ER-positive, HER2-negative, node-negative breast cancer. Cancer Research 80:P5-06-05, 2020
15. Sparano JA, Gray RJ, Ravdin PM, et al: Clinical and Genomic Risk to Guide the Use of Adjuvant Therapy for Breast Cancer. New England Journal of Medicine 380:2395-2405, 2019
16. Sparano JA, Gray RJ, Makower DF, et al: Adjuvant Chemotherapy Guided by a 21-Gene Expression Assay in Breast Cancer. New England Journal of Medicine 379:111-121, 2018

Supplementary Figure and Table legends:

Supplementary Table 1: Clinico-pathological characteristics of patient sub-groups in the TEAM Trial cohort ER+ve/HER2-ve population:

ER+ve/HER2-ve = Estrogen positive HER2 negative. HR+ve/HER2any = cases with either ER or progesterone receptor positive HER2 positive/negative cases. N-ve no chemotherapy = node negative cases treated without chemotherapy. N+ve no chemotherapy = node positive cases treated without chemotherapy. Chemotherapy = all cases (both node negative and node positive) treated with chemotherapy. Age = age at diagnosis.

Supplementary Table 2: Cross tabulation of Signature-trained results in the TEAM study for all ER+ve/HER-ve patients.

For comparisons between Oncotype-trained and Prosigna-trained n = 3286. For comparisons between Oncotype-trained and MammaPrint-trained n= 3290. Results for Prosigna-trained incorporate tumour size (4 samples were missing tumour size values and hence no Prosgina-trained result was available).

Supplementary Table 3: Cross tabulation of Signature-trained results in the TEAM study for Node negative, ER+ve/HER-ve patients treated without chemotherapy.

For comparisons between Oncotype-Trained and Prosigna-Trained n = 971. For comparisons between Oncotype-Trained and MammaPrint-Trained n= 972. Prosigna-trained results include tumour size (hence 1 sample had missing values).

Supplementary Table 4: Oncotype-trained results stratified by other test results, binary classification:
HR = Hazard ratio. 95%CI = 95% confidence interval. P* = p value of log-rank test to compare survival distributions. REF = reference group. P = p value of Wald test for comparison versus reference (low risk) group. DMFS = Distant metastasis free survival at 10 years (see text). (N) = number of cases in subgroups. All cases = all ER+ve/HER2-ve cases. N-Ch- = Node negative cases treated without chemotherapy. N+Ch- = Node positive cases treated without chemotherapy. Ch+ = cases treated with chemotherapy (node negative and node positive combined). Int = intermediate.

Supplementary Table 5: Prosigna-trained results stratified by other test results, binary classification:
HR = Hazard ratio. 95%CI = 95% confidence interval. P* = p value of log-rank test to compare survival distributions. REF = reference group. P = p value of Wald test for comparison versus reference (low risk) group. DMFS = Distant metastasis free survival at 10 years (see text). (N) = number of cases in subgroups. All cases = all ER+ve/HER2-ve cases. N-Ch- = Node negative cases treated without chemotherapy. N+Ch- = Node positive cases treated without chemotherapy. Ch+ = cases treated with chemotherapy (node negative and node positive combined). Int = intermediate.

Supplementary Table 6: Mammaprint-trained results stratified by other test results, binary classification:
HR = Hazard ratio. 95%CI = 95% confidence interval. P* = p value of log-rank test to compare survival distributions. REF = reference group. P = p value of Wald test for comparison versus

reference (low risk) group. DMFS = Distant metastasis free survival at 10 years (see text). (N) = number of cases in subgroups. All cases = all ER+ve/HER2-ve cases. N-Ch- = Node negative cases treated without chemotherapy. N+Ch- = Node positive cases treated without chemotherapy. Ch+ = cases treated with chemotherapy (node negative and node positive combined). Int = intermediate.

Supplementary Table 7: Cross tabulation of Signature-trained results in the TEAM study for Node positive, ER+ve/HER-ve patients treated without chemotherapy.

For comparisons between Oncotype-Trained and Prosigna-Trained n = 1246. For comparisons between Oncotype-Trained and MammaPrint-Trained n= 1247. Results for Prosigna-Trained represent ROR-PT scores incorporating tumour size (1 sample had missing tumour size).

Supplementary Table 8: Cross tabulation of Signature-trained results in the TEAM study for Node ER+ve/HER-ve patients treated with chemotherapy.

For comparisons between Oncotype-Trained and Prosigna-Trained n = 1060. For comparisons between Oncotype-Trained and MammaPrint-Trained n= 1064. Results for Prosigna-Trained represent ROR-PT scores incorporating tumour size (2 samples had missing values).

Supplementary Table 9: Likelihood χ^2ratios by test in HR+ve/HERany cohort:

Likelihood χ^2 ratios(LRχ^2) for univariate(single test) or bivariate(two tests in sequence) derived using 10 year distant metastasis free survival as end point. HR+/HER2any cases =all HR+ve cases (irrespective of HER2 status, nodal status and chemotherapy). ΔLRχ^2 = change in LRχ^2 for comparison of 2 tests versus a single test.

Supplementary Table 10: Oncotype-trained results stratified by other test results, trinary classification HR+ve/HER2any population:

HR = Hazard ratio. 95%CI = 95% confidence interval. P* = p value of log-rank test to compare survival distributions. REF = reference group. P = p value of Wald test for comparison versus reference (low risk) group. DMFS = Distant metastasis free survival at 10 years (see text). (N) = number of cases in subgroups. All cases = all HR+ve/HER2+ve & HER2-ve cases. N-Ch- = Node negative cases treated without chemotherapy. N+Ch- = Node positive cases treated without chemotherapy. Ch+ = cases treated with chemotherapy (node negative and node positive combined). Int = intermediate.

Supplementary Table 11: Cross tabulation of Signature-trained results in the TEAM study for all cases HR+ve HER2+ve and HER2-ve

Low = low risk, Int = intermediate risk, High = high risk. N = number of cases (3811).

Supplementary Table 12: Prosigna-trained results stratified by other test results, trinary classification HR+ve/HER2 any population:

HR = Hazard ratio. 95%CI = 95% confidence interval. P* = p value of log-rank test to compare survival distributions. REF = reference group. P = p value of Wald test for comparison versus reference (low risk) group. DMFS = Distant metastasis free survival at 10 years (see text). (N) = number of cases in subgroups. All cases = all HR+ve/HER2+ve & HER2-ve cases. N-Ch- = Node negative cases treated without chemotherapy. N+Ch- = Node positive cases treated without

chemotherapy. Ch+ = cases treated with chemotherapy (node negative and node positive combined). Int = intermediate.

Supplementary Table 13: Mammaprint-trained results stratified by other test results, trinary classification HR+ve/HER2any population:
HR = Hazard ratio. 95%CI = 95% confidence interval. P* = p value of log-rank test to compare survival distributions. REF = reference group. P = p value of Wald test for comparison versus reference (low risk) group. DMFS = Distant metastasis free survival at 10 years (see text). (N) = number of cases in subgroups. All cases = all HR+ve/HER2+ve & HER2-ve cases. N-Ch- = Node negative cases treated without chemotherapy. N+Ch- = Node positive cases treated without chemotherapy. Ch+ = cases treated with chemotherapy (node negative and node positive combined). Int = intermediate.

Supplementary Table 14: Cross tabulation of Signature-trained results in the TEAM study for Node negative, HR+ve HER2+ve and HER2-ve patients treated without chemotherapy.

Low = low risk, Int = intermediate risk, High = high risk. N = number of cases.

Supplementary Table 15: Cross tabulation of Signature-trained results in the TEAM study for Node positive, HR+ve HER2+ve and HER2-ve patients treated without chemotherapy.

Low = low risk, Int = intermediate risk, High = high risk. N = number of cases.

Supplementary Table 16: Cross tabulation of Signature-trained results in the TEAM study for HR+ve HER2+ve and HER2-ve patients treated with chemotherapy.

Low = low risk, Int = intermediate risk, High = high risk. N = number of cases.

Supplementary Table 17: 10 year DMFS by molecular risk group across all cases and by subgroups, combining ER+ve/HER2-ve cases and HR+ve HERany.

ER+ve/HER-ve = ER positive HER2 negative cases (see main paper and figures). Figures represent 10 year DMFS in percentage. Figures in brackets represent 95% confidence intervals for estimates. Data summarizes data presented in Forest plots in the main paper and supplementary data for signature trained results.

Supplementary Figure 1: Performance of BINARY test results in ER+ve/HER-ve sample from TEAM pathology cohort.

Kaplan-Meier survival curves with Log-rank Hazard ratios all for cases of ER+ve, HER2-ve breast cancer from the TEAM cohort for binary test groupings for Oncotype-trained (Panel A), Prosigna-trained (Panel B) and for ER+ve/HER2-ve node negative cases treated without chemotherapy for Oncotype-Trained (Panel C) and Prosigna-trained results. Log-Rank P values for each test are in brackets. Within each panel low (green) and high (red) risk survival curves are plotted with LogRank Hazard ratios for high risk and intermediate risk (Oncotype-Like and Prosigna-like only) calculated against low risk cases in each sub-group. 95% Confidence intervals for LogRank

Hazard ratios are in brackets. For each group the number at risk (Low, intermediate, high) are presented under the X axis.

Supplementary Figure 2: Forest plot of DMFS10 by test and reclassification Binary Oncotype first, all ER+ve/HER2-ve cases.

DMFS10 = Distant metastasis free survival at 10 years post diagnosis. (95% CI) = 95% confidence interval. P = p value. N = number of cases in each subgroup. % = percentage of cases within each risk strata. X Axis = percent distant metastasis free survival. Open boxes represent primary test DMFS10 by risk group. Solid boxes represent sub-stratification by secondary tests with 95% confidence intervals (bars). Top Panel = Oncotype-trained low risk cases stratified by Prosigna-trained and Mammaprint-trained results. Bottom Panel = Oncotype-trained high risk group.

Supplementary Figure 3: Forest plot of DMFS10 by test and reclassification Binary Oncotype first, ER+ve/HER2-ve Node negative no chemotherapy.

DMFS10 = Distant metastasis free survival at 10 years post diagnosis. (95% CI) = 95% confidence interval. P = p value. N = number of cases in each subgroup. % = percentage of cases within each risk strata. X Axis = percent distant metastasis free survival. Open boxes represent primary test DMFS10 by risk group. Solid boxes represent sub-stratification by secondary tests with 95% confidence intervals (bars). Top Panel = Oncotype-trained low risk cases stratified by Prosigna-trained and Mammaprint-trained results. Bottom Panel = Oncotype-trained high risk group.

Supplementary Figure 4: Forest plot of DMFS10 by test and reclassification Binary Prosigna first, all ER+ve/HER2-ve cases.

DMFS10 = Distant metastasis free survival at 10 years post diagnosis. (95% CI) = 95% confidence interval. P = p value. N = number of cases in each subgroup. % = percentage of cases within each risk strata. X Axis = percent distant metastasis free survival. Open boxes represent primary test DMFS10 by risk group. Solid boxes represent sub-stratification by secondary tests with 95% confidence intervals (bars). Top Panel = Prosigna-trained low risk cases stratified by Oncotype-trained and Mammaprint-trained results. Bottom Panel = Prosigna-trained high risk group.

Supplementary Figure 5: Forest plot of DMFS10 by test and reclassification Binary Prosigna first, ER+ve/HER2-ve Node negative no chemotherapy.

DMFS10 = Distant metastasis free survival at 10 years post diagnosis. (95% CI) = 95% confidence interval. P = p value. N = number of cases in each subgroup. % = percentage of cases within each risk strata. X Axis = percent distant metastasis free survival. Open boxes represent primary test DMFS10 by risk group. Solid boxes represent sub-stratification by secondary tests with 95% confidence intervals (bars). Top Panel = Prosigna-trained low risk cases stratified by Oncotype-trained and Mammaprint-trained results. Bottom Panel = Prosigna-trained high risk group.

Supplementary Figure 6: Forest plot of DMFS10 by binary test and reclassification Mammaprint first, all ER+ve/HER2-ve cases.

DMFS10 = Distant metastasis free survival at 10 years post diagnosis. (95% CI) = 95% confidence interval. P = p value. N = number of cases in each subgroup. % = percentage of cases within each risk strata. X Axis = percent distant metastasis free survival. Open boxes represent primary test DMFS10 by risk group. Solid boxes represent sub-stratification by secondary tests with 95% confidence intervals (bars). Top Panel = Mammaprint-trained low risk cases stratified by binary

Oncotype-trained and Prosigna-trained results. Bottom Panel = Mammaprint-trained high risk group.

Supplementary Figure 7: Forest plot of DMFS10 by binary test and reclassification Mammaprint first, ER+ve/HER2-ve Node negative no chemotherapy.

DMFS10 = Distant metastasis free survival at 10 years post diagnosis. (95% CI) = 95% confidence interval. P = p value. N = number of cases in each subgroup. % = percentage of cases within each risk strata. X Axis = percent distant metastasis free survival. Open boxes represent primary test DMFS10 by risk group. Solid boxes represent sub-stratification by secondary tests with 95% confidence intervals (bars). Top Panel = Mammaprint-trained low risk cases stratified by binary Oncotype-trained and Prosigna-trained results. Bottom Panel = Mammaprint-trained high risk group.

Supplementary Figure 8: Performance of "signature-trained" test results in ER+ve/HER-ve Node positive samples treated without chemotherapy from the TEAM pathology cohort.

Kaplan-Meier survival curves with Log-rank Hazard ratios all for cases of ER+ve, HER2-ve breast node positive cancers treated without chemotherapy from the TEAM cohort for test groupings for Oncotype-trained (Panel A), Prosigna-trained (Panel B) and Mammaprint-trained (Panel C) in ER+ve/HER2-ve node negative cases treated without chemotherapy Log-Rank P values for each test are in brackets. Within each panel low (green), intermediate (Prosigna-trained and Oncotype-trained only - blue) and high (red) risk survival curves are plotted with LogRank Hazard ratios for high risk and intermediate risk (Oncotype-Like and Prosigna-like only) calculated against low risk cases in each sub-group. 95% Confidence intervals for LogRank Hazard ratios are in brackets. For each group the number at risk (Low, intermediate, high) are presented under the X axis.

Supplementary Figure 9: Forest plot of DMFS10 by test and reclassification with Oncotype first in ER+ve/HER2-ve node positive cases treated without chemotherapy.

DMFS10 = Distant metastasis free survival at 10 years post diagnosis. (95% CI) = 95% confidence interval. P = p value. N = number of cases in each subgroup. % = percentage of cases within each risk strata. X Axis = percent distant metastasis free survival. Open boxes represent primary test DMFS10 by risk group. Solid boxes represent sub-stratification by secondary tests with 95% confidence intervals (bars). Top Panel = Oncotype-trained low risk cases stratified by Prosigna-trained and Mammaprint-trained results. Middle panel = Oncotype Trained-intermediate risk group. Bottom Panel = Oncotype-trained high risk group.

Supplementary Figure 10: Performance of "signature-trained" test results in ER+ve/HER-ve cases treated with chemotherapy from the TEAM pathology cohort.

Kaplan-Meier survival curves with Log-rank Hazard ratios all for cases of ER+ve, HER2-ve breast node positive cancers treated without chemotherapy from the TEAM cohort for test groupings for Oncotype-trained (Panel A), Prosigna-trained (Panel B) and Mammaprint-trained (Panel C) in ER+ve/HER2-ve node negative cases treated without chemotherapy Log-Rank P values for each test are in brackets. Within each panel low (green), intermediate (Prosigna-trained and Oncotype-trained only - blue) and high (red) risk survival curves are plotted with LogRank Hazard ratios for high risk and intermediate risk (Oncotype-Like and Prosigna-like only) calculated against low risk cases in each sub-group. 95% Confidence intervals for LogRank Hazard ratios are in brackets. For each group the number at risk (Low, intermediate, high) are presented under the X axis.

Supplementary Figure 11: Forest plot of DMFS10 by test and reclassification with Oncotype first in ER+ve/HER2-ve cases treated with chemotherapy.

DMFS10 = Distant metastasis free survival at 10 years post diagnosis. (95% CI) = 95% confidence interval. P = p value. N = number of cases in each subgroup. % = percentage of cases within each risk strata. X Axis = percent distant metastasis free survival. Open boxes represent primary test DMFS10 by risk group. Solid boxes represent sub-stratification by secondary tests with 95% confidence intervals (bars). Top Panel = Oncotype-trained low risk cases stratified by Prosigna-trained and Mammaprint-trained results. Middle panel = Oncotype Trained-intermediate risk group. Bottom Panel = Oncotype-trained high risk group.

Supplementary Figure 12: Forest plot of DMFS10 by test and reclassification with Prosigna first in ER+ve/HER2-ve node positive cases treated without chemotherapy.

DMFS10 = Distant metastasis free survival at 10 years post diagnosis. (95% CI) = 95% confidence interval. P = p value. N = number of cases in each subgroup. % = percentage of cases within each risk strata. X Axis = percent distant metastasis free survival. Open boxes represent primary test DMFS10 by risk group. Solid boxes represent sub-stratification by secondary tests with 95% confidence intervals (bars). Top Panel = Prosigna-trained low risk cases stratified by Oncotype-trained and Mammaprint-trained results. Middle panel = Prosigna Trained-intermediate risk group. Bottom Panel = Prosigna-trained high risk group.

Supplementary Figure 13: Forest plot of DMFS10 by test and reclassification with Prosigna first in ER+ve/HER2-ve cases treated with chemotherapy.

DMFS10 = Distant metastasis free survival at 10 years post diagnosis. (95% CI) = 95% confidence interval. P = p value. N = number of cases in each subgroup. % = percentage of cases within each risk strata. X Axis = percent distant metastasis free survival. Open boxes represent primary test DMFS10 by risk group. Solid boxes represent sub-stratification by secondary tests with 95% confidence intervals (bars). Top Panel = Prosigna-trained low risk cases stratified by Oncotype-trained and Mammaprint-trained results. Middle panel = Prosigna Trained-intermediate risk group. Bottom Panel = Prosigna-trained high risk group.

Supplementary Figure 14: Forest plot of DMFS10 by test and reclassification with Mammaprint first in ER+ve/HER2-ve node positive cases treated without chemotherapy.

DMFS10 = Distant metastasis free survival at 10 years post diagnosis. (95% CI) = 95% confidence interval. P = p value. N = number of cases in each subgroup. % = percentage of cases within each risk strata. X Axis = percent distant metastasis free survival. Open boxes represent primary test DMFS10 by risk group. Solid boxes represent sub-stratification by secondary tests with 95% confidence intervals (bars). Top Panel = Mammaprint-trained low risk cases stratified by Oncotype-trained and Prosigna-trained results. Bottom Panel = Mammaprint-trained high risk group.

Supplementary Figure 15: Forest plot of DMFS10 by test and reclassification with Mammaprint first in ER+ve/HER2-ve cases treated with chemotherapy.

DMFS10 = Distant metastasis free survival at 10 years post diagnosis. (95% CI) = 95% confidence interval. P = p value. N = number of cases in each subgroup. % = percentage of cases within each risk strata. X Axis = percent distant metastasis free survival. Open boxes represent primary test

DMFS10 by risk group. Solid boxes represent sub-stratification by secondary tests with 95% confidence intervals (bars). Top Panel = Mammaprint-trained low risk cases stratified by Oncotype-trained and Prosigna-trained results. Bottom Panel = Mammaprint-trained high risk group.

Supplementary Figure 16: Kaplan-Meier survival curves with Log-rank Hazard ratios for cases of HR+ve, HER2+ve and HER2-ve breast cancer from the TEAM cohort for Oncotype-trained (Panel A), Prosigna-trained (Panel B) and Mammaprint-trained results. Log-Rank P values for each test are in brackets. Within each panel low (green), intermediate (blue) and high (red) risk survival curves are plotted with LogRank Hazard ratios for high risk and intermediate risk (Oncotype-Like and Prosigna-like only) calculated against low risk cases in each sub-group. 95% Confidence intervals for LogRank Hazard ratios are in brackets. For each group the number at risk (Low, intermediate, high) are presented under the X axis.

Supplementary Figure 17: Forest plot of Oncotype-trained test results re-stratified by other tests, all HR+ve, HER2+ve and HER2-ve cases

DMFS10 = Distant metastasis free survival at 10 years post diagnosis. (95% CI) = 95% confidence interval. P = p value. N = number of cases in each subgroup. % = percentage of cases within each risk strata. X Axis = percent distant metastasis free survival. Open boxes represent primary test DMFS10 by risk group. Solid boxes represent sub-stratification by secondary tests with 95% confidence intervals (bars). Top Panel = Oncotype-trained low risk cases stratified by Prosigna-trained and Mammaprint-trained results. Middle Panel = Oncotype-trained moderate risk group. Bottom Panel = Oncotype-trained high risk group.

Supplementary Figure 18: Forest plot of Prosigna-trained test results re-stratified by other tests, all HR+ve, HER2+ve and HER2-ve cases

DMFS10 = Distant metastasis free survival at 10 years post diagnosis. (95% CI) = 95% confidence interval. P = p value. N = number of cases in each subgroup. % = percentage of cases within each risk strata. X Axis = percent distant metastasis free survival. Open boxes represent primary test DMFS10 by risk group. Solid boxes represent sub-stratification by secondary tests with 95% confidence intervals (bars). Top Panel = Prosigna-trained low risk cases stratified by Oncotype-trained and Mammaprint-trained results. Middle Panel = Prosigna-trained moderate risk group. Bottom Panel = Prosigna-trained high risk group.

Supplementary Figure 19: Forest plot of Mammaprint-trained test results re-stratified by other tests, all HR+ve, HER2+ve and HER2-ve cases

DMFS10 = Distant metastasis free survival at 10 years post diagnosis. (95% CI) = 95% confidence interval. P = p value. N = number of cases in each subgroup. % = percentage of cases within each risk strata. X Axis = percent distant metastasis free survival. Open boxes represent primary test DMFS10 by risk group. Solid boxes represent sub-stratification by secondary tests with 95% confidence intervals (bars). Top Panel = Mammaprint-trained low risk cases stratified by Oncotype-trained and Prosigna-trained results. Bottom Panel = Mammaprint-trained high risk group.

Supplementary Figure 20: Kaplan-Meier survival curves with Log-rank Hazard ratios for cases of HR+ve, HER2+ve and HER2-ve Node negative breast cancer treated without chemotherapy from the TEAM cohort for Oncotype-trained (Panel A), Prosigna-trained (Panel B) and Mammaprint-

trained results. Log-Rank P values for each test are in brackets. Within each panel low (green), intermediate (blue) and high (red) risk survival curves are plotted with LogRank Hazard ratios for high risk and intermediate risk (Oncotype-Like and Prosigna-like only) calculated against low risk cases in each sub-group. 95% Confidence intervals for LogRank Hazard ratios are in brackets. For each group the number at risk (Low, intermediate, high) are presented under the X axis.

Supplementary Figure 21: Forest plot of Oncotype-trained test results re-stratified by other tests, HR+ve, HER2+ve and HER2-ve cases treated without chemotherapy

DMFS10 = Distant metastasis free survival at 10 years post diagnosis. (95% CI) = 95% confidence interval. P = p value. N = number of cases in each subgroup. % = percentage of cases within each risk strata. X Axis = percent distant metastasis free survival. Open boxes represent primary test DMFS10 by risk group. Solid boxes represent sub-stratification by secondary tests with 95% confidence intervals (bars). Top Panel = Oncotype-trained low risk cases stratified by Prosigna-trained and Mammaprint-trained results. Middle Panel = Oncotype-trained moderate risk group. Bottom Panel = Oncotype-trained high risk group.

Supplementary Figure 22: Kaplan-Meier survival curves with Log-rank Hazard ratios for cases of HR+ve, HER2+ve and HER2-ve Node positive breast cancer treated without chemotherapy from the TEAM cohort for Oncotype-trained (Panel A), Prosigna-trained (Panel B) and Mammaprint-trained results. Log-Rank P values for each test are in brackets. Within each panel low (green), intermediate (blue) and high (red) risk survival curves are plotted with LogRank Hazard ratios for high risk and intermediate risk (Oncotype-Like and Prosigna-like only) calculated against low risk cases in each sub-group. 95% Confidence intervals for LogRank Hazard ratios are in brackets. For each group the number at risk (Low, intermediate, high) are presented under the X axis.

Supplementary Figure 23: Forest plot of DMFS10 by test and reclassification with Oncotype first in HR+ve, HER2+ve and HER2-ve, node positive cases treated without chemotherapy.

DMFS10 = Distant metastasis free survival at 10 years post diagnosis. (95% CI) = 95% confidence interval. P = p value. N = number of cases in each subgroup. % = percentage of cases within each risk strata. X Axis = percent distant metastasis free survival. Open boxes represent primary test DMFS10 by risk group. Solid boxes represent sub-stratification by secondary tests with 95% confidence intervals (bars). Top Panel = Oncotype-trained low risk cases stratified by Prosigna-trained and Mammaprint-trained results. Middle panel = Oncotype Trained-intermediate risk group. Bottom Panel = Oncotype-trained high risk group.

Supplementary Figure 24: Kaplan-Meier survival curves with Log-rank Hazard ratios for cases of HR+ve, HER2+ve and HER2-ve, Node any breast cancer treated with chemotherapy from the TEAM cohort for Oncotype-trained (Panel A), Prosigna-trained (Panel B) and Mammaprint-trained results. Log-Rank P values for each test are in brackets. Within each panel low (green), intermediate (blue) and high (red) risk survival curves are plotted with LogRank Hazard ratios for high risk and intermediate risk (Oncotype-Like and Prosigna-like only) calculated against low risk cases in each sub-group. 95% Confidence intervals for LogRank Hazard ratios are in brackets. For each group the number at risk (Low, intermediate, high) are presented under the X axis.

Supplementary Figure 25: Forest plot of DMFS10 by test and reclassification with Oncotype first in HR+ve, HER2+ve and HER2-ve cases treated with chemotherapy.

DMFS10 = Distant metastasis free survival at 10 years post diagnosis. (95% CI) = 95% confidence interval. P = p value. N = number of cases in each subgroup. % = percentage of cases within each risk strata. X Axis = percent distant metastasis free survival. Open boxes represent primary test DMFS10 by risk group. Solid boxes represent sub-stratification by secondary tests with 95% confidence intervals (bars). Top Panel = Oncotype-trained low risk cases stratified by Prosigna-trained and Mammaprint-trained results. Middle panel = Oncotype Trained-intermediate risk group. Bottom Panel = Oncotype-trained high risk group.

Supplementary Figure 26: Forest plot of Prosigna-trained test results re-stratified by other tests, Node-ve, HR+ve, HER2+ve and HER2-ve cases treated without chemotherapy

DMFS10 = Distant metastasis free survival at 10 years post diagnosis. (95% CI) = 95% confidence interval. P = p value. N = number of cases in each subgroup. % = percentage of cases within each risk strata. X Axis = percent distant metastasis free survival. Open boxes represent primary test DMFS10 by risk group. Solid boxes represent sub-stratification by secondary tests with 95% confidence intervals (bars). Top Panel = Prosigna-trained low risk cases stratified by Oncotype-trained and Mammaprint-trained results. Middle Panel = Prosigna-trained moderate risk group. Bottom Panel = Prosigna-trained high risk group.

Supplementary Figure 27: Forest plot of DMFS10 by test and reclassification with Prosigna first in HR+ve, HER2+ve and HER2-ve, node positive cases treated without chemotherapy.

DMFS10 = Distant metastasis free survival at 10 years post diagnosis. (95% CI) = 95% confidence interval. P = p value. N = number of cases in each subgroup. % = percentage of cases within each risk strata. X Axis = percent distant metastasis free survival. Open boxes represent primary test DMFS10 by risk group. Solid boxes represent sub-stratification by secondary tests with 95% confidence intervals (bars). Top Panel = Prosigna-trained low risk cases stratified by Oncotype-trained and Mammaprint-trained results. Middle panel = Prosigna Trained-intermediate risk group. Bottom Panel = Prosigna-trained high risk group.

Supplementary Figure 28: Forest plot of DMFS10 by test and reclassification with Prosigna first in HR+ve, HER2+ve and HER2-ve cases treated with chemotherapy.

DMFS10 = Distant metastasis free survival at 10 years post diagnosis. (95% CI) = 95% confidence interval. P = p value. N = number of cases in each subgroup. % = percentage of cases within each risk strata. X Axis = percent distant metastasis free survival. Open boxes represent primary test DMFS10 by risk group. Solid boxes represent sub-stratification by secondary tests with 95% confidence intervals (bars). Top Panel = Prosigna-trained low risk cases stratified by Oncotype-trained and Mammaprint-trained results. Middle panel = Prosigna Trained-intermediate risk group. Bottom Panel = Prosigna-trained high risk group.

Supplementary Figure 29: Forest plot of Mammaprint-trained test results re-stratified by other tests, Node-ve, HR+ve, HER2+ve and HER2-ve cases treated without chemotherapy

DMFS10 = Distant metastasis free survival at 10 years post diagnosis. (95% CI) = 95% confidence interval. P = p value. N = number of cases in each subgroup. % = percentage of cases within each risk strata. X Axis = percent distant metastasis free survival. Open boxes represent primary test DMFS10 by risk group. Solid boxes represent sub-stratification by secondary tests with 95% confidence intervals (bars). Top Panel = Mammaprint-trained low risk cases stratified by

Oncotype-trained and Prosigna-trained results. Bottom Panel = Mammaprint-trained high risk group.

Supplementary Figure 30: Forest plot of DMFS10 by test and reclassification with Mammaprint first in HR+ve, HER2+ve and HER2-ve node positive cases treated without chemotherapy.

DMFS10 = Distant metastasis free survival at 10 years post diagnosis. (95% CI) = 95% confidence interval. P = p value. N = number of cases in each subgroup. % = percentage of cases within each risk strata. X Axis = percent distant metastasis free survival. Open boxes represent primary test DMFS10 by risk group. Solid boxes represent sub-stratification by secondary tests with 95% confidence intervals (bars). Top Panel = Mammaprint-trained low risk cases stratified by Oncotype-trained and Prosigna-trained results. Bottom Panel = Mammaprint-trained high risk group.

Supplementary Figure 31: Forest plot of DMFS10 by test and reclassification with Mammaprint first in HR+ve, HER2+ve and HER2-ve cases treated with chemotherapy.

DMFS10 = Distant metastasis free survival at 10 years post diagnosis. (95% CI) = 95% confidence interval. P = p value. N = number of cases in each subgroup. % = percentage of cases within each risk strata. X Axis = percent distant metastasis free survival. Open boxes represent primary test DMFS10 by risk group. Solid boxes represent sub-stratification by secondary tests with 95% confidence intervals (bars). Top Panel = Mammaprint-trained low risk cases stratified by Oncotype-trained and Prosigna-trained results. Bottom Panel = Mammaprint-trained high risk group.

Supplementary Tables:

Supplementary Table 1: Clinico-pathological characteristics of patient sub-groups in the TEAM Trial cohort ER+ve/HER2-ve population.

	ER+/HER2-ve				HR+ve/HER2any			
Characteristic	All	N-ve no chemotherapy	N+ no chemotherapy	Chemotherapy	All	N-ve no chemotherapy	N+ no chemotherapy	Chemotherapy
Age ≤55	760 (13.7%)	133 (7.5%)	76 (4.5%)	551 (28.0%)	876 (14.3%)	160 (8.1%)	84 (4.5%)	632 (28.1%)
Age >55	4683 (86.0%)	1635 (92.5%)	1618 (95.5%)	1414 (72.0%)	5232 (85.7%)	1826 (91.9%)	1770 (95.5%)	1616 (71.9%)
Grade 1	587 (10.8%)	87 (4.9%)	350 (20.7%)	149 (7.6%)	616 (10.1%)	90 (4.5%)	367 (19.8%)	158 (7.0%)
Grade 2	2894 (53.2%)	1023 (57.9%)	854 (50.4%)	1011 (51.5%)	3163 (51.8%)	1118 (56.3%)	930 (50.2%)	1106 (49.2%)
Grade 3	1504 (27.6%)	521 (29.5%)	355 (21.0%)	622 (31.7%)	1829 (29.9%)	632 (31.8%)	414 (22.3%)	776 (34.5%)
Missing	458 (8.4%)	137 (7.8%)	135 (8.0%)	183 (9.3%)	500 (8.2%)	146 (7.4%)	143 (7.7%)	208 (9.3%)
Tumour size								
≤2.0	2625 (48.2%)	971 (54.9%)	807 (47.6%)	842 (42.9%)	2908 (47.6%)	1094 (55.1%)	868 (46.8%)	939 (41.8%)
>2-5.0	2265 (41.6%)	658 (37.2%)	739 (43.6%)	860 (43.8%)	2583 (42.3%)	739 (37.2%)	831 (44.8%)	1004 (44.7%)
>5.0	228 (4.2%)	34 (1.9%)	81 (4.8%)	111 (5.7%)	258 (4.2%)	41 (2.1%)	87 (4.7%)	128 (5.7%)
Missing	325 (5.97%)	105 (5.9%)	67 (4.0%)	152 (7.7%)	359 (5.9%)	112 (5.6%)	68 (3.7%)	177 (7.9%)
Number positive nodes								
0	2294 (42.2%)	N/A	N/A	526 (26.8%)	2601 (42.6%)	N/A	N/A	614 (27.3%)
1-3	1972 (36.2%)	N/A	1253 (74.0%)	718 (36.5%)	2178 (35.7%)	N/A	1368 (73.8%)	809 (36.0%)
4-9	471 (8.7%)	N/A	186 (11.0%)	285 (14.5%)	548 (9.0%)	N/A	210 (11.3%)	338 (15.0%)
10+	192 (3.5%)	N/A	70 (4.1%)	120 (6.1%)	234 (3.8%)	N/A	84 (4.5%)	148 (6.6%)
missing	514 (9.4%)	N/A	185 (10.9%)	316 (16.1%)	547 (9.0%)	N/A	192 (10.4%)	339 (15.1%)
HER2								
Positive	N/A	N/A	N/A	N/A	559 (9.2%)	193 (9.7%)	152 (8.2%)	212 (9.4%)
Negative	N/A	N/A	N/A	N/A	3820 (62.5%)	1169 (58.9%)	1378 (74.3%)	1261 (56.1%)
Missing	N/A	N/A	N/A	N/A	1729 (28.3%)	624 (31.4%)	324 (17.5%)	775 (34.5%)
chemo								
yes	1965 (36.1)	N/A	N/A	N/A	2248 (36.8%)	N/A	N/A	N/A
no	3475 (63.9%)	N/A	N/A	N/A	3856 (63.1%)	N/A	N/A	N/A
Missing	3 (0.1%)	N/A	N/A	N/A	4 (0.1%)	N/A	N/A	N/A

Supplementary Table 2: Cross tabulation of Signature-trained results in the TEAM study for all ER+ve/HER-ve patients.

		MammaPrint-trained		Oncotype-trained			Prosigna-trained		
		Low	High	Low	Int	High	Low	Int	High
MammaPrint Trained	Low			1483	645	52	814	944	422
	High			124	528	452	12	211	881
Oncotype Trained	Low	1483	124				643	685	279
	Int	645	528				174	381	618
	High	52	452				9	89	406
Prosigna-trained	Low	814	12	643	174	9			
	Int	944	211	685	381	89			
	High	422	881	279	618	406			

Supplementary Table 3: ER+ve/HER2-ve/Node Negative No chemotherapy.

		MammaPrint-trained		Oncotype-trained			Prosigna-trained		
		Low	High	Low	Int	High	Low	Int	High
MammaPrint Trained	Low			407	174	18	217	249	133
	High			51	175	145	3	72	296
Oncotype-Trained	Low	407	51				174	189	95
	Int	174	175				43	106	200
	High	18	145				3	26	134
Prosigna-trained	Low	217	3	174	43	3			
	Int	249	72	189	106	26			
	High	133	296	95	200	134			

Supplementary Table 4: Binary test classification: Oncotype-trained results stratified by other test results.

Oncotype Trained First						
	Oncotype Trained <25			Oncotype Trained ≥25		
	HR (95% CI)	DMFS (95% CI)	P* (N)	HR (95% CI)	DMFS (95% CI)	P (N)
All Cases	REF	85.8 (84.1-87.4)	<0.001 (2361)	2.58 (2.17-3.07)	70.2 (66.9-73.3)	<0.001 (923)
N-Ch-	REF	90.8 (87.7-93.1)	<0.001 (674)	2.71 (1.82-4.03)	80.2 (74.6-84.7)	<0.001 (296)
N+Ch-	REF	85.3 (82.5-87.7)	<0.001 (948)	3.53 (2.71-4.60)	60.5 (54.2-66.3)	<0.001 (299)
Ch+	REF	81.9 (78.5-84.8)	<0.001 (733)	1.92 (1.45-2.54)	70.4 (64.7-75.4)	<0.001 (327)

	Prosigna Trained <61			Prosigna Trained ≥61			Prosigna Trained <61			Prosigna Trained ≥61		
	HR	DMFS	P*	HR	DMFS	P	HR	DMFS	P*	HR	DMFS	P
ALL Cases	REF	89.0 (87.2-90.6)	<0.001 (1742)	2.33 (1.83-2.97)	76.5 (72.3-80.1)	<0.001 (619)	REF	79.6 (73.5-84.5)	<0.001 (239)	1.75 (1.26-2.42)	66.9 (62.9-70.6)	0.001 (684)
N-Ch-	REF	93.4 (90.0-95.7)	<0.001 (469)	2.59 (1.45-4.62)	84.7 (77.7-89.7)	0.001 (205)	REF	92.1 (81.9-96.7)	0.009 (72)	3.21 (1.28-8.08)	76.5 (69.7-82.0)	0.013 (224)
N+Ch-	REF	88.7 (85.8-91.1)	<0.001 (717)	2.55 (1.75-3.71)	73.8 (66.3-79.9)	<0.001 (231)	REF	79.5 (67.4-87.5)	<0.001 (77)	2.77 (1.58-4.86)	53.8 (46.3-60.6)	<0.001 (222)
Ch+	REF	85.7 (81.9-88.7)	<0.001 (553)	2.31 (1.58-3.37)	70.2 (61.8-77.1)	<0.001 (180)	REF	70.3 (58.9-79.0)	0.841 (90)	0.95 (0.60-1.52)	70.4 (63.5-76.2)	0.841 (237)

	Mammaprint Trained Low			Mammaprint Trained High			Mammaprint Trained Low			Mammaprint Trained High		
	HR	DMFS	P*	HR	DMFS	P	HR	DMFS	P*	HR	DMFS	P
ALL Cases	REF	88.0 (86.2-89.6)	<0.001 (1983)	2.31 (1.78-3.01)	74.3 (68.8-79.0)	<0.001 (378)	REF	75.9 (68.6-81.7)	0.044 (197)	1.40 (1.01-1.96)	68.7 (64.9-72.2)	0.045 (726)
N-Ch-	REF	93.3 (83.0-98.9)	<0.001 (548)	3.39 (1.89-6.07)	80.2 (70.6-86.9)	<0.001 (126)	REF	95.5 (83.0-98.9)	0.008 (51)	5.49 (1.33-22.6)	77.1 (70.7-82.4)	0.018 (245)
N+Ch-	REF	87.9 (85.0-90.2)	<0.001 (813)	2.84 (1.90-4.25)	69.6 (59.7-77.5)	<0.001 (135)	REF	67.7 (55.3-77.3)	0.106 (83)	1.45 (0.92-2.29)	57.7 (50.2-64.5)	0.108 (216)
Ch+	REF	83.4 (79.8-86.5)	0.020 (618)	1.69 (1.08-2.64)	73.3 (62.5-81.8)	0.021 (115)	REF	71.6 (57.5-81.8)	0.674 (63)	1.13 (0.66-1.95)	70.1 (63.6-75.6)	0.648 (264)

P*: p-value of log-rank test to compare survival distributions. (Global statistical significance of the model) P : p-value of Wald-test to evaluate whether the hazard ratio is 1. (Statistical significance of each individual coefficient)

Supplementary Table 5: Binary test classification: Prosigna-trained results stratified by other test results.

Prosigna Trained First												
	Prosigna Trained <61						Prosigna Trained ≥61					
	HR (95% CI)			DMFS (95% CI)		P* (N)	HR (95% CI)			DMFS (95% CI)		P (N)
All Cases	REF			87.9 (86.1-89.4)		<0.001 (1981)	2.73 (2.28-3.26)			71.4 (68.6-74.1)		<0.001 (1303)
N-Ch-	REF			93.2 (90.1-95.4)		<0.001 (541)	3.50 (2.25-5.46)			80.5 (75.7-84.3)		<0.001 (429)
N+Ch-	REF			87.8 (84.9-90.1)		<0.001 (794)	3.67 (2.78-4.83)			63.9 (58.6-68.7)		<0.001 (453)
Ch+	REF			83.5 (80.0-86.5)		<0.001 (643)	1.98 (1.50-2.62)			70.4 (65.2-74.9)		<0.001 (417)
	Oncotype Trained <25			Oncotype Trained ≥25			Oncotype Trained <25			Oncotype Trained ≥25		
	HR	DMFS	P*	HR	DMFS	P	HR	DMFS	P*	HR	DMFS	P
ALL Cases	REF	89.0 (87.2-90.6)	<0.001 (1742)	2.20 (1.58-3.08)	79.6 (73.5-84.5)	<0.001 (239)	REF	76.5 (72.3-80.1)	<0.001 (619)	1.68 (1.34-2.11)	66.9 (62.9-70.6)	<0.001 (684)
N-Ch-	REF	93.4 (90.0-95.7)	0.419 (469)	1.49 (0.56-3.93)	92.1 (81.9-96.7)	0.422 (72)	REF	84.7 (77.7-89.7)	0.012 (205)	1.87 (1.14-3.06)	76.5 (69.7-82.0)	0.013 (224)
N+Ch-	REF	88.7 (85.8-91.1)	0.012 (717)	2.06 (1.16-3.31)	79.5 (67.4-87.5)	0.014 (77)	REF	73.8 (66.3-79.9)	<0.001 (231)	2.33 (1.64-3.31)	53.8 (46.3-60.6)	<0.001 (222)
Ch+	REF	85.7 (81.9-88.7)	<0.001 (553)	2.58 (1.63-4.09)	70.3 (58.9-79.0)	<0.001 (90)	REF	70.2 (61.8-77.1)	0.730 (180)	1.07 (0.73-1.57)	70.4 (63.5-76.2)	0.730 (237)
	Mammaprint Trained Low			Mammaprint Trained High			Mammaprint Trained Low			Mammaprint Trained High		
	HR	DMFS	P*	HR	DMFS	P	HR	DMFS	P*	HR	DMFS	P
ALL Cases	REF	88.9 (87.1-90.5)	<0.001 (1758)	2.05 (1.45-2.92)	80.0 (73.4-85.1)	<0.001 (223)	REF	78.1 (73.0-82.3)	<0.001 (422)	1.64 (1.26-2.12)	68.4 (64.9-71.7)	<0.001 (881)
N-Ch-	REF	94.7 (91.5-96.7)	0.003 (466)	3.17 (1.42-7.06)	84.6 (71.6-91.9)	0.005 (75)	REF	89.6 (80.6-94.5)	0.002 (133)	2.78 (1.43-5.44)	76.5 (70.6-81.4)	0.003 (296)
N+Ch-	REF	88.1 (85.2-90.6)	0.283 (730)	1.46 (0.73-2.92)	84.3 (71.9-91.6)	0.286 (64)	REF	75.7 (66.8-82.5)	<0.001 (166)	2.09 (1.41-3.10)	57.4 (50.8-63.5)	<0.001 (287)
Ch+	REF	85.1 (81.4-88.2)	<0.001 (559)	2.27 (1.39-3.70)	73.1 (61.6-81.6)	0.001 (84)	REF	69.0 (58.8-77.2)	0.948 (122)	0.99 (0.65-1.49)	70.9 (64.7-76.2)	0.948 (295)

Supplementary Table 6: Binary test classification: Mammaprint-trained results stratified by other test results.

Mammaprint Trained First						
	Mammaprint Trained Low			Mammaprint Trained High		
	HR (95% CI)	DMFS (95% CI)	P* (N)	HR (95% CI)	DMFS (95% CI)	P (N)
All Cases	REF	86.9 (85.1-88.4)	<0.001 (2180)	2.64 (2.22-3.14)	70.7 (67.6-73.6)	<0.001 (1104)
N-Ch-	REF	93.5 (90.5-95.6)	<0.001 (599)	4.23 (2.72-6.56)	78.2 (73.0-82.5)	<0.001 (371)
N+Ch-	REF	85.9 (83.1-88.3)	<0.001 (896)	3.34 (2.56-4.36)	62.4 (56.5-67.7)	<0.001 (351)
Ch+	REF	82.3 (78.8-85.3)	<0.001 (681)	1.86 (1.41-2.46)	71.2 (65.8-75.9)	<0.001 (379)

	Oncotype Trained <25			Oncotype Trained ≥25			Oncotype Trained <25			Oncotype Trained ≥25		
	HR	DMFS	P*	HR	DMFS	P	HR	DMFS	P*	HR	DMFS	P
ALL Cases	REF	88.0 (86.2-89.6)	<0.001 (1983)	2.35 (1.68-3.28)	75.9 (68.6-81.7)	<0.001 (197)	REF	74.3 (68.8-79.0)	0.006 (378)	1.44 (1.11-1.87)	68.7 (64.9-72.2)	0.006 (726)
N-Ch-	REF	93.3 (90.1-95.5)	0.799 (548)	0.83 (0.20-3.50)	95.5 (83.0-98.9)	0.800 (51)	REF	80.2 (70.6-86.9)	0.248 (126)	1.36 (0.81-2.28)	77.1 (70.7-82.4)	0.249 (245)
N+Ch-	REF	87.9 (85.0-90.2)	<0.001 (813)	3.32 (2.10-5.24)	67.7 (55.3-77.3)	<0.001 (83)	REF	69.6 (59.7-77.5)	0.007 (135)	1.72 (1.15-2.57)	57.7 (50.2-64.5)	0.008 (216)
Ch+	REF	83.4 (79.8-86.5)	0.017 (618)	1.89 (1.11-3.23)	71.6 (57.5-81.8)	0.019 (63)	REF	73.3 (62.5-81.5)	0.272 (115)	1.29 (0.82-2.03)	70.1 (63.6-75.6)	0.274 (264)
	Prosigna Trained <61			Prosigna Trained ≥61			Prosigna Trained <61			Prosigna Trained ≥61		
	HR	DMFS	P*	HR	DMFS	P	HR	DMFS	P*	HR	DMFS	P
ALL Cases	REF	89.9 (87.1-90.5)	<0.001 (1758)	2.14 (1.62-2.82)	78.1 (73.0-82.3)	<0.001 (422)	REF	80.0 (73.4-85.1)	0.002 (223)	1.70 (1.21-2.38)	68.4 (64.9-71.7)	0.002 (881)
N-Ch-	REF	94.7 (91.5-96.7)	0.069 (466)	2.02 (0.93-4.38)	89.6 (80.6-94.5)	0.074 (133)	REF	84.6 (71.6-91.9)	0.098 (75)	1.79 (0.89-3.61)	76.5 (70.6-81.4)	0.103 (296)
N+Ch-	REF	88.1 (85.2-90.6)	<0.001 (730)	2.29 (1.51-3.48)	75.7 (66.8-82.5)	<0.001 (166)	REF	84.3 (71.9-91.6)	<0.001 (64)	3.23 (1.64-6.39)	57.4 (50.8-63.5)	0.001 (287)
Ch+	REF	85.1 (81.4-88.2)	<0.001 (559)	2.31 (1.52-3.51)	69.0 (58.8-77.2)	<0.001 (122)	REF	73.1 (61.6-81.6)	0.980 (84)	1.01 (0.62-1.63)	70.9 (64.7-76.2)	0.980 (295)

Supplementary Table 7: ER+ve/HER2-ve/Node Positive No chemotherapy.

		MammaPrint-trained		Oncotype-trained			Prosigna-trained		
		Low	High	Low	Int	High	Low	Int	High
MammaPrint Trained	Low			639	237	20	315	415	166
	High			44	166	141	3	61	287
Oncotype-Trained	Low	639	44				263	309	111
	Int	237	166				54	142	207
	High	20	141				1	25	135
Prosigna-trained	Low	315	3	263	54	1			
	Int	415	61	309	142	25			
	High	166	287	111	207	135			

Supplementary Table 8: ER+ve/HER2-ve any nodal status, treatment includes chemotherapy.

		MammaPrint-trained		Oncotype-trained			Prosigna-trained		
		Low	High	Low	Int	High	Low	Int	High
MammaPrint Trained	Low			434	233	14	281	278	122
	High			29	185	165	6	78	295
Oncotype-Trained	Low	434	29				205	185	73
	Int	233	185				77	133	208
	High	14	165				5	38	136
Prosigna-trained	Low	281	6	205	77	5			
	Int	278	78	185	133	38			
	High	122	295	73	208	136			

Supplementary Table 9: Likelihood Chi-square ratios by test and cohort all cases.

LRχ2 = Likelihood Ratio Chi-Squared Value	HR+/HER2any (N=3811)		
***All models run exiting at 10 years*	df	LRχ2	p-value
Univariate Models			
Oncotype-trained	2	172.8	<0.0001
Prosigna-trained	2	170.0	<0.0001
Mammaprint-trained	1	160.4	<0.0001
Bivariate Models			
Oncotype-trained + Prosigna-trained	4	225.3	<0.0001
Oncotype-trained + Mammaprint-trained	3	201.1	<0.0001
Prosigna-trained + Mammaprint-trained	3	206.0	<0.0001
Bivariate vs. Univariate		ΔLRχ2	p-value
Oncotype-trained + Prosigna-trained vs. Oncotype-trained	2	52.5	<0.0001
Oncotype-trained + Mammaprint-trained vs. Oncotype-trained	1	28.3	<0.0001
Prosigna-trained + Oncotype-trained vs. Prosigna-trained	2	55.3	<0.0001
Prosigna-trained + Mammaprint.trained vs. Prosigna-trained	1	36.0	<0.0001
Mammaprint-trained + Oncotype-trained vs. Mammaprint=trained	2	40.7	<0.0001
Mammaprint-trained + Prosigna-trained vs. Mammaprint-trained	2	45.6	<0.0001

Supplementary Table 10: Oncotype-trained results stratified by other test results (HR+ve/HER2+ve and HER-ve; trinary classification).

	Oncotype-Trained First																										
	Oncotype-Trained Low risk									Oncotype-Trained Intermediate Risk									Oncotype-Trained High Risk								
	HR (95% CI)			DMFS (95% CI)			P* (N)			HR (95% CI)			DMFS (95% CI)			P (N)			HR (95% CI)			DMFS (95% CI)			P (N)		
All Cases	REF			88.0 (86.0-89.7)			<0.001 (1679)			1.97 (1.61-2.41)			79.3 (76.7-81.6)			<0.001 (1287)			3.67 (3.01-4.47)			66.5 (62.9-69.8)			<0.001 (845)		
N-Ch-	REF			92.6 (89.1-95.1)			<0.001 (480)			2.07 (1.27-3.37)			86.6 (82.2-90.0)			0.003 (391)			4.88 (3.10-7.68)			73.0 (66.7-78.3)			<0.001 (279)		
N+Ch-	REF			86.2 (83.0-88.9)			<0.001 (710)			1.93 (1.42-2.63)			77.7 (73.0-81.7)			<0.001 (435)			4.46 (3.30-6.02)			56.8 (49.7-63.2)			<0.001 (241)		
Ch+	REF			85.9 (81.9-89.1)			<0.001 (486)			2.03 (1.46-2.81)			74.4 (69.7-78.6)			<0.001 (457)			2.87 (2.06-3.99)			68.3 (62.6-73.3)			<0.001 (323)		
	Prosigna-Trained Low			Prosigna trained Int			Prosigna Trained High			Prosigna-Trained Low			Prosigna trained Int			Prosigna Trained High			Prosigna-Trained Low			Prosigna trained Int			Prosigna Trained High		
	HR	DMFS	P*	HR	DMFS	P	HR	DMFS	P	HR	DMFS	P*	HR	DMFS	P	HR	DMFS	P	HR	DMFS	P*	HR	DMFS	P	HR	DMFS	P
ALL Cases	REF	92.2 (89.6-94.2)	<0.001 (669)	1.36 (0.93-2.00)	88.6 (85.6-91.1)	0.113 (716)	3.12 (2.09-4.66)	75.6 (68.7-81.2)	<0.001 (294)	REF	90.9 (84.8-94.6)	<0.001 (186)	2.13 (1.20-3.80)	82.0 (77.5-85.7)	0.010 (423)	3.22 (1.86-5.56)	74.4 (70.5-77.8)	<0.001 (678)	REF	59.3 (27.5-81.0)	0.095 (14)	0.56 (0.22-1.43)	74.5 (66.1-81.1)	0.796 (154)	0.82 (0.34-1.98)	64.9 (60.9-68.6)	0.656 (677)
N-Ch-	REF	96.7 (92.2-98.6)	0.023 (182)	1.99 (0.69-5.74)	92.7 (86.9-96.0)	0.201 (199)	4.01 (1.37-11.7)	84.6 (71.6-92.0)	0.011 (99)	REF	94.1 (65.0-99.1)	0.098 (46)	4.52 (0.59-34.8)	87.2 (78.0-92.8)	0.147 (121)	6.20 (0.85-45.5)	84.4 (78.3-88.9)	0.073 (224)	REF	50.0 (11.1-80.4)	0.014 (6)	0.12 (0.02-0.61)	92.3 (77.5-97.5)	0.010 (44)	0.51 (0.16-1.62)	70.2 (63.1-76.2)	0.252 (229)
N+Ch-	REF	89.6 (84.8-93.0)	<0.001 (270)	1.00 (0.58-1.72)	88.8 (84.0-92.2)	0.987 (322)	3.04 (1.74-5.29)	69.3 (57.2-78.5)	<0.001 (118)	REF	92.9 (82.1-97.3)	<0.001 (57)	2.12 (0.73-6.15)	82.5 (74.4-88.3)	0.167 (155)	4.22 (1.53-11.6)	70.4 (63.2-76.4)	0.005 (223)	REF	100	0.123 (1)	NA	72.8 (53.8-84.9)	NA (37)	NA	53.7 (46.1-60.8)	NA (203)
Ch+	REF	91.9 (86.7-95.1)	0.001 (216)	1.85 (0.98-3.51)	84.2 (77.1-89.2)	0.060 (193)	3.46 (1.71-7.01)	73.1 (58.9-83.1)	0.001 (77)	REF	87.5 (77.0-93.4)	0.008 (83)	2.07 (0.98-4.34)	77.2 (69.0-83.5)	0.055 (147)	2.80 (1.39-5.64)	68.0 (60.5-74.3)	0.004 (227)	REF	66.7 (19.5-90.4)	0.833 (7)	0.83 (0.20-3.51)	64.9 (51.5-75.5)	0.798 (73)	0.74 (0.18-3.02)	69.2 (62.7-74.9)	0.675 (243)
	Mammaprint-Trained Low						Mammaprint Trained High			Mammaprint-Trained Low						Mammaprint Trained High			Mammaprint-Trained Low						Mammaprint Trained High		
	HR	DMFS	P*				HR	DMFS	P	HR	DMFS	P*				HR	DMFS	P	HR	DMFS	P*				HR	DMFS	P
ALL Cases	REF	89.2 (87.2-90.9)	<0.001 (1548)				2.74 (1.81-4.15)	72.7 (62.0-80.9)	<0.001 (131)	REF	83.6 (80.3-86.4)	<0.001 (703)				1.68 (1.30-2.19)	74.0 (69.8-77.8)	<0.001 (584)	REF	70.1 (57.6-79.5)	0.263 (89)				1.28 (0.83-1.99)	66.1 (62.3-69.6)	0.264 (756)
N-Ch-	REF	93.8 (90.2-96.2)	0.006 (425)				3.18 (1.33-7.56)	82.6 (66.0-91.6)	0.009 (55)	REF	92.0 (86.1-95.5)	0.003 (197)				2.60 (1.34-5.07)	81.4 (74.4-86.6)	0.005 (194)	REF	92.7 (73.9-98.1)	0.043 (28)				3.86 (0.95-15.8)	70.9 (64.2-76.6)	0.060 (251)
N+Ch-	REF	87.5 (84.3-90.1)	<0.001 (663)				3.02 (1.63-5.59)	66.7 (47.8-80.2)	<0.001 (47)	REF	84.9 (79.2-89.1)	<0.001 (254)				2.33 (1.51-3.62)	67.8 (59.7-74.7)	<0.001 (181)	REF	57.2 (35.3-74.0)	0.766 (31)				1.10 (0.59-2.06)	56.7 (49.3-63.6)	0.766 (210)
Ch+	REF	87.2 (83.2-90.3)	0.002 (457)				3.13 (1.48-6.62)	64.2 (37.6-81.8)	0.003 (29)	REF	76.3 (70.0-81.5)	0.342 (251)				1.21 (0.82-1.78)	72.0 (64.2-78.4)	0.342 (206)	REF	62.8 (39.8-79.1)	0.992 (30)				1.01 (0.51-2.00)	68.8 (62.9-74.0)	0.982 (293)

P*: p-value of log-rank test to compare survival distributions. (Global statistical significance of the model)

P : p-value of Wald-test to evaluate whether the hazard ratio is 1. (Statistical significance of each individual coefficient)

Supplementary Table 11: Cross Tabulation All cases HR+ve HER2+ve and HER2-ve.

N=3811		MammaPrint-trained		Oncotype trained			Prosigna-trained		
		Low	High	Low	Int	High	Low	Int	High
MammaPrint Trained	Low			1548	703	89	852	1017	471
	High			131	584	756	17	276	1178
Oncotype Trained	Low	1548	131				669	716	294
	Int	703	584				186	423	678
	High	89	756				14	154	677
Prosigna-trained	Low	852	17	661	186	14			
	Int	1017	276	716	423	154			
	High	471	1178	294	678	677			

Supplementary Table 12: Prosigna-trained results stratified by other test results (HR+ve/HER2+ve and HER-ve; trinary classification).

	Prosigna-Trained First																										
	Prosigna-Trained Low risk									Prosigna-Trained Intermediate Risk									Prosigna-Trained High Risk								
	HR (95% CI)			DMFS (95% CI)			P* (N)			HR (95% CI)			DMFS (95% CI)			P (N)			HR (95% CI)			DMFS (95% CI)			P (N)		
All Cases	REF			91.4 (89.1-93.3)			<0.001 (869)			1.80 (1.35-2.41)			84.8 (82.4-86.9)			<0.001 (1293)			3.97 (3.05-5.17)			70.8 (68.2-73.1)			<0.001 (1649)		
N-Ch-	REF			95.0 (90.4-97.4)			<0.001 (234)			1.87 (0.87-3.98)			90.9 (86.7-93.8)			0.106 (364)			5.08 (2.57-10.1)			78.6 (74.5-82.2)			<0.001 (552)		
N+Ch-	REF			90.1 (85.9-93.1)			<0.001 (328)			1.40 (0.89-2.19)			85.8 (81.9-88.9)			0.146 (514)			4.44 (2.98-6.63)			64.0 (59.3-68.3)			<0.001 (544)		
Ch+	REF			90.1 (85.7-93.2)			<0.001 (306)			2.34 (1.50-3.64)			78.3 (73.5-82.3)			<0.001 (413)			3.54 (2.33-5.36)			69.4 (65.0-73.4)			<0.001 (547)		
	Oncotype-trained Low			Oncotype-trained Int			Oncotype-Trained High			Oncotype-Trained Low			Oncotype-trained Int			Oncotype-Trained High			Oncotype-trained Low			Oncotype-trained Int			Oncotype-Trained High		
	HR	DMFS	P*	HR	DMFS	P	HR	DMFS	P	HR	DMFS	P*	HR	DMFS	P	HR	DMFS	P	HR	DMFS	P*	HR	DMFS	P	HR	DMFS	P
ALL Cases	REF	92.2 (89.6-94.2)	<0.001 (669)	1.17 (0.64-2.13)	90.9 (84.8-94.6)	0.617 (186)	7.24 (2.87-18.3)	59.3 (27.5-81.0)	<0.001 (14)	REF	88.6 (85.6-91.1)	<0.001 (716)	1.83 (1.29-2.58)	82.0 (77.5-85.7)	0.001 (423)	2.92 (1.93-4.41)	74.5 (66.1-81.1)	<0.001 (154)	REF	75.6 (68.7-81.2)	<0.001 (294)	1.24 (0.90-1.69)	74.4 (70.5-77.8)	0.189 (678)	1.90 (1.40-2.57)	64.9 (60.9-68.6)	<0.001 (677)
N-Ch-	REF	96.7 (92.2-98.6)	<0.001 (182)	0.82 (0.10-7.00)	94.1 (65.0-99.1)	0.854 (46)	21.5 (5.13-90.3)	50.0 (11.1-80.4)	<0.001 (6)	REF	92.7 (86.9-96.0)	0.324 (199)	1.85 (0.82-4.20)	87.2 (78.0-92.8)	0.140 (121)	1.30 (0.36-4.68)	92.3 (77.5-97.5)	0.683 (44)	REF	84.6 (71.6-92.0)	<0.001 (99)	1.28 (0.62-2.61)	84.4 (78.3-88.9)	0.503 (224)	2.74 (1.40-5.36)	70.2 (63.1-76.2)	0.003 (229)
N+Ch-	REF	89.6 (84.8-93.0)	0.886 (270)	0.82 (0.28-2.36)	92.9 (82.1-97.3)	0.712 (57)	NA	100	NA (1)	REF	88.8 (84.2-92.2)	0.003 (322)	1.79 (1.02-3.13)	82.5 (74.4-88.3)	0.041 (155)	3.32 (1.56-7.03)	72.7 (53.9-84.9)	0.002 (37)	REF	69.3 (57.2-78.5)	<0.001 (118)	1.21 (0.76-1.92)	70.4 (63.2-76.4)	0.421 (223)	2.16 (1.39-3.35)	53.7 (46.1-60.8)	0.001 (203)
Ch+	REF	91.9 (86.7-95.1)	0.017 (216)	1.55 (0.68-3.55)	87.5 (77.0-93.4)	0.297 (83)	6.56 (1.50-28.8)	66.7 (19.5-90.4)	0.013 (7)	REF	84.2 (77.1-89.2)	<0.001 (193)	1.73 (1.02-2.92)	77.2 (69.0-83.5)	0.043 (147)	2.86 (1.62-5.04)	64.9 (51.5-75.5)	<0.001 (73)	REF	73.1 (58.9-83.1)	0.517 (77)	1.28 (0.74-2.21)	68.0 (60.5-74.3)	0.385 (227)	1.38 (0.80-2.37)	69.2 (62.7-74.9)	0.250 (243)
	Mammaprint-Trained Low						Mammaprint Trained High			Mammaprint-Trained Low						Mammaprint Trained High			Mammaprint-Trained Low						Mammaprint Trained High		
	HR	DMFS	P*				HR	DMFS	P	HR	DMFS	P*				HR	DMFS	P	HR	DMFS	P*				HR	DMFS	P
ALL Cases	REF	91.9 (89.6-93.7)	<0.001 (852)				5.13 (2.06-12.8)	67.0 (37.7-84.9)	<0.001 (17)	REF	86.4 (83.8-88.7)	<0.001 (1017)				1.83 (1.31-2.54)	78.7 (72.8-83.4)	<0.001 (276)	REF	78.0 (73.2-82.0)	<0.001 (471)				1.69 (1.33-2.15)	68.0 (64.9-70.8)	<0.001 (1178)
N-Ch-	REF	95.9 (91.3-98.1)	<0.001 (228)				11.4 (2.35-54.7)	66.7 (19.5-90.4)	0.002 (6)	REF	93.2 (88.5-96.0)	0.015 (269)				2.51 (1.16-5.43)	84.3 (73.5-91.0)	0.019 (95)	REF	89.6 (81.7-94.2)	<0.001 (153)				2.94 (1.61-5.38)	74.5 (69.4-78.9)	<0.001 (399)
N+Ch-	REF	90.0 (85.8-93.1)	0.600 (325)				NA	100	NA (3)	REF	86.5 (82.3-89.8)	0.137 (441)				1.61 (0.85-3.03)	81.4 (69.4-89.0)	0.141 (73)	REF	75.7 (67.3-82.2)	<0.001 (182)				2.08 (1.43-3.01)	58.5 (52.7-63.8)	<0.001 (362)
Ch+	REF	91.0 (86.6-94.0)	<0.001 (298)				6.97 (2.08-23.3)	53.6 (13.2-82.5)	0.002 (8)	REF	80.5 (74.9-85.0)	0.019 (305)				1.73 (1.09-2.74)	72.4 (62.4-80.1)	0.020 (108)	REF	68.1 (58.4-76.1)	0.791 (135)				1.05 (0.72-1.53)	69.8 (64.7-74.3)	0.791 (412)

Supplementary Table 13: Mammaprint-trained results stratified by other test results (HR+ve/HER2+ve and HER-ve; trinary classification).

	Mammaprint-trained first																	
	Mammaprint-Trained Low risk									Mammaprint-Trained High Risk								
	HR (95% CI)			DMFS (95% CI)			P* (N)			HR (95% CI)			DMFS (95% CI)			P (N)		
All Cases	REF			86.7 (85.0-88.2)			<0.001 (2342)			2.71 (2.32-3.18)			70.0 (67.3-72.4)			<0.001 (1475)		
N-Ch-	REF			93.2 (90.4-95.3)			<0.001 (650)			4.35 (2.94-6.43)			76.1 (71.6-80.0)			<0.001 (501)		
N+Ch-	REF			85.7 (83.0-88.1)			<0.001 (949)			3.33 (2.60-4.26)			62.5 (57.4-67.3)			<0.001 (438)		
Ch+	REF			82.3 (78.9-85.2)			<0.001 (739)			1.98 (1.54-2.54)			70.2 (65.8-74.2)			<0.001 (531)		
	Oncotype-trained Low			Oncotype-trained Int			Oncotype-Trained High			Oncotype-trained Low			Oncotype-trained Int			Oncotype-Trained High		
	HR	DMFS	P*	HR	DMFS	P	HR	DMFS	P	HR	DMFS	P*	HR	DMFS	P	HR	DMFS	P
ALL Cases	REF	89.2 (87.2-90.9)	<0.001 (1548)	1.74 (1.34-2.25)	83.4 (80.0-86.2)	<0.001 (705)	3.32 (2.12-5.22)	70.1 (57.6-79.5)	<0.001 (89)	REF	72.7 (62.0-80.9)	<0.001 (131)	1.09 (0.72-1.65)	74.0 (69.7-77.7)	0.687 (586)	1.59 (1.07-2.37)	66.2 (62.4-69.7)	0.023 (758)
N-Ch-	REF	93.8 (90.2-96.2)	0.562 (425)	1.41 (0.68-2.91)	92.0 (86.1-95.5)	0.351 (197)	1.67 (0.39-7.17)	92.7 (73.9-98.1)	0.491 (28)	REF	82.6 (66.0-91.6)	0.013 (55)	1.23 (0.54-2.78)	81.0 (74.0-86.2)	0.623 (195)	2.11 (0.97-4.61)	70.9 (64.2-76.6)	0.060 (251)
N+Ch-	REF	87.5 (84.3-90.1)	<0.001 (663)	1.46 (0.96-2.20)	84.5 (78.9-88.8)	0.075 (255)	4.70 (2.48-8.90)	57.2 (35.3-74.0)	<0.001 (31)	REF	66.7 (47.8-80.2)	0.022 (47)	1.16 (0.62-2.17)	67.8 (59.7-74.7)	0.651 (181)	1.76 (0.96-3.23)	56.7 (49.3-63.6)	0.066 (210)
Ch+	REF	87.2 (83.2-90.3)	<0.001 (457)	2.10 (1.43-3.10)	75.9 (69.6-81.1)	<0.001 (252)	3.20 (1.57-6.52)	62.8 (39.8-79.1)	0.001 (30)	REF	64.2 (37.6-81.8)	0.399 (29)	0.82 (0.39-1.73)	72.2 (64.4-78.5)	0.599 (207)	1.04 (0.50-2.15)	69.0 (63.2-74.2)	0.913 (295)
	Prosigna-Trained Low			Prosigna trained Int			Prosigna Trained High			Prosigna-Trained Low			Prosigna trained Int			Prosigna Trained High		
	HR	DMFS	P*	HR	DMFS	P	HR	DMFS	P	HR	DMFS	P*	HR	DMFS	P	HR	DMFS	P
ALL Cases	REF	91.9 (89.6-93.7)	<0.001 (852)	1.65 (1.20-2.27)	86.4 (83.8-88.7)	0.002 (1017)	2.87 (2.05-4.02)	78.0 (73.2-82.0)	<0.001 (471)	REF	67.0 (37.7-84.9)	0.006 (17)	0.60 (0.24-1.49)	78.7 (72.8-83.4)	0.269 (276)	0.96 (0.40-2.31)	68.0 (64.9-70.8)	0.920 (1178)
N-Ch-	REF	95.9 (91.3-98.1)	0.112 (228)	1.67 (0.67-4.13)	93.2 (88.5-96.0)	0.270 (269)	2.62 (1.03-6.65)	89.6 (81.7-94.2)	0.043 (153)	REF	66.7 (19.5-90.4)	0.103 (6)	0.37 (0.08-1.65)	84.3 (73.5-91.0)	0.192 (95)	0.68 (0.17-2.77)	74.5 (69.4-78.9)	0.594 (399)
N+Ch-	REF	90.0 (85.8-93.1)	<0.001 (325)	1.28 (0.80-2.05)	86.5 (82.3-89.8)	0.294 (441)	2.63 (1.60-4.33)	75.7 (67.3-82.2)	<0.001 (182)	REF	100	0.002 (3)	NA	81.4 (69.4-89.0)	NA (73)	NA	58.5 (52.7-63.8)	NA (362)
Ch+	REF	91.0 (86.5-94.0)	<0.001 (298)	2.20 (1.35-3.61)	80.5 (74.9-85.0)	0.002 (305)	3.81 (2.26-6.42)	68.1 (58.4-76.1)	<0.001 (135)	REF	53.6 (13.2-82.5)	0.650 (8)	0.57 (0.17-1.88)	72.4 (62.4-80.1)	0.358 (108)	0.60 (0.19-1.89)	69.8 (64.7-74.3)	0.384 (412)

Supplementary Table 14: HR+ve HER2+ve & HER2-ve, Node negative No chemotherapy.

N= 1153/1154		MammaPrint-trained		Oncotype Dx-trained			Prosigna-trained		
		Low	High	Low	Int	High	Low	Int	High
MammaPrint Trained	Low			425	197	28	228	269	153
	High			55	194	251	6	95	399
Oncotype Dx Trained	Low	425	55				182	199	99
	Int	197	194				46	121	224
	High	28	251				6	44	229
Prosigna-trained	Low	228	6	182	46	6			
	Int	269	95	199	121	44			
	High	153	399	99	224	229			

For comparisons between Oncotype-Trained and Prosigna-Trained n = 1154. For comparisons between Oncotype-Trained and MammaPrint-Trained n= 1153 Results for Prosigna-Trained represent Prosigna-trained-ROR-PT scores incorporating tumour size (hence 1 sample had missing value).

Supplementary Table 15: HR+ve HER2+ve & HER2-ve, Node positive No chemotherapy.

N=1389/1390		MammaPrint-trained		Oncotype Dx-trained			Prosigna-trained		
		Low	High	Low	Int	High	Low	Int	High
MammaPrint Trained	Low			663	254	31	325	441	182
	High			47	181	210	3	73	362
Oncotype Dx Trained	Low	663	47				270	322	118
	Int	254	181				57	155	223
	High	31	210				1	37	203
Prosigna-trained	Low	325	3	270	57	1			
	Int	441	73	322	155	37			
	High	182	362	118	223	203			

For comparisons between Oncotype-Trained and Prosigna-Trained n = 1390 For comparisons between Oncotype-Trained and MammaPrint-Trained n= 1389 Results for Prosigna-Trained represent Prosigna-trained-ROR-PT scores incorporating tumour size (hence 1 sample had missing value).

Supplementary Table 16: HR+ve HER2+ve & HER2-ve, Any Nodal status/Chemotherapy.

N=1268/1272		MammaPrint-trained		Oncotype Dx-trained			Prosigna-trained		
		Low	High	Low	Int	High	Low	Int	High
MammaPrint Trained	Low			457	251	30	298	305	135
	High			29	206	293	8	108	412
Oncotype Dx Trained	Low	457	29				216	193	77
	Int	251	206				83	147	227
	High	30	293				7	73	243
Prosigna-trained	Low	298	8	216	83	7			
	Int	305	108	193	147	73			
	High	135	412	77	227	243			

For comparisons between Oncotype-Trained and Prosigna-Trained n = 1272 For comparisons between Oncotype-Trained and MammaPrint-Trained n= 1268 Results for Prosigna-Trained represent Prosigna-trained-ROR-PT scores incorporating tumour size (hence 4 sample had missing values).

Supplementary Table 17: 10 Year DMFS by molecular risk group across all cases and by subgroups.

Signature	Risk group		
	Low	Intermediate	High
	ALL		
	ER+ve/HER-ve cases		
Oncotype-Trained	87.9 (85.8-89.6)	78.6 (75.9-81.1)	67.5 (62.8-71.7)
Prosigna-trained	92.1 (89.8-94.0)	84.9 (82.3-87.1)	71.4 (68.6-74.1)
MammaPrint-trained	86.9 (85.1-88.4)	N/A	70.7 (67.6-73.6)
	HR+ve/HERany cases		
Oncotype-Trained	88.0 (86.0-89.7)	79.3 (76.7-81.6)	66.5 (62.9-69.8)
Prosigna-trained	91.4 (89.1-93.3)	84.8 (82.4-86.9)	70.8 (68.2-73.1)
MammaPrint-trained	86.8 (85.1-88.3)	N/A	69.9 (67.3-72.4)
	NODE NEGATIVE NO CHEMOTHERAPY		
	ER+ve/HER-ve cases		
Oncotype-Trained	92.5 (88.8-95.0)	86.3 (81.6-89.9)	76.7 (68.4-83.0)
Prosigna-trained	96.7 (92.0-98.7)	91.0 (86.4-94.1)	80.5 (75.8-84.3)
MammaPrint-trained	93.5 (90.5-95.6)	N/A	78.2 (73.0-82.5)
	HR+ve/HERany cases		
Oncotype-Trained	92.6 (89.1-95.1)	86.6 (82.2-90.0)	73.0 (66.7-78.3)
Prosigna-trained	95.0 (90.4-97.4)	90.9 (86.7-93.8)	78.6 (74.5-82.2)
MammaPrint-trained	93.2 (90.4-95.3)	N/A	76.3 (71.8-80.1)
	NODE POSITIVE NO CHEMOTHERAPY		
	ER+ve/HER-ve cases		
Oncotype-Trained	86.4 (83.1-89.1)	77.0 (72.0-81.2)	55.1 (46.4-63.0)
Prosigna-trained	90.1 (85.8-93.2)	86.2 (82.1-89.4)	63.9 (58.6-68.7)
MammaPrint-trained	85.9 (83.1-88.3)	N/A	62.4 (56.5-67.7)
	HR+ve/HERany cases		
Oncotype-Trained	86.2 (83.0-88.9)	77.7 (73.0-81.7)	56.8 (49.7-63.2)
Prosigna-trained	90.1 (85.9-93.1)	85.8 (81.9-88.9)	64.0 (59.3-68.3)
MammaPrint-trained	85.8 (83.1-88.2)	N/A	62.5 (57.4-67.3)
	CHEMOTHERAPY TREATED		
	ER+ve/HER-ve cases		
Oncotype-Trained	85.4 (81.2-88.8)	73.8 (68.8-78.2)	70.8 (63.0-77.2)
Prosigna-trained	90.9 (86.3-94.0)	77.8 (72.5-82.2)	70.4 (65.2-74.9)
MammaPrint-trained	82.3 (78.8-85.3)	N/A	71.2 (65.8-75.9)
	HR+ve/HERany cases		
Oncotype-Trained	85.9 (81.9-89.1)	74.4 (69.7-78.6)	68.3 (62.6-73.3)
Prosigna-trained	90.1 (85.7-93.2)	78.3 (73.5-82.3)	69.4 (65.0-73.4)
MammaPrint-trained	82.4 (79.1-85.3)	N/A	70.0% (65.6-74.1)

Risk groups (low, intermediate, high) represent those described for each test in the text). For intermediate or moderate risk (terminology used for Oncotype recurrence scores and Prosigna scores respectively) cases we use the term intermediate risk throughout. Results are 10 year distant metastasis free survival as percentages with 95% confidence intervals (95%CI) in brackets.

Supplementary Figure 1: Performance of BINARY test results in ER+ve/HER-ve samples from TEAM pathology cohort

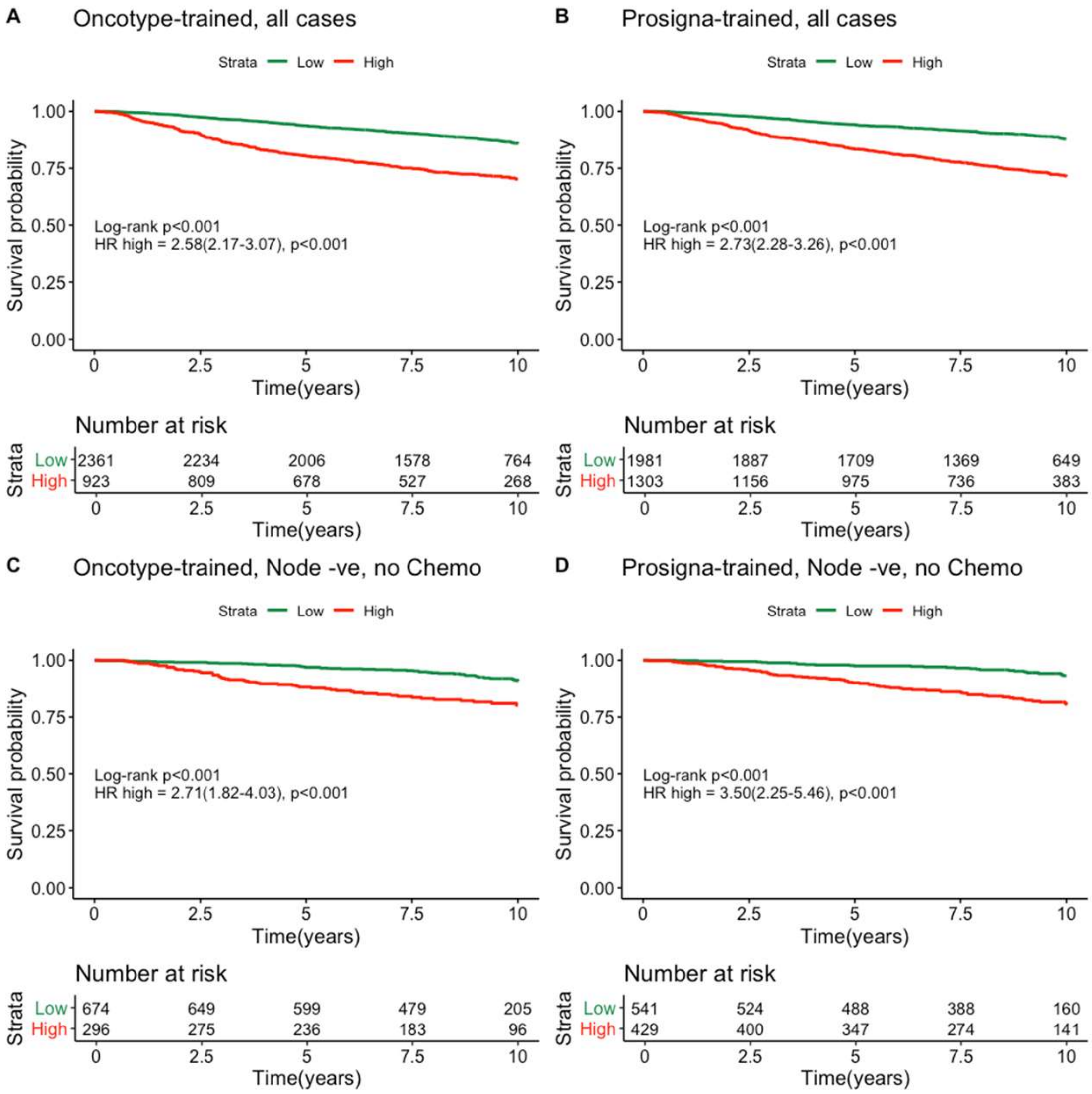

Supplementary Figure 2: Forest plot of DMFS10 by test and reclassification: Binary Oncotype first, All ER+ve/HER2-ve cases

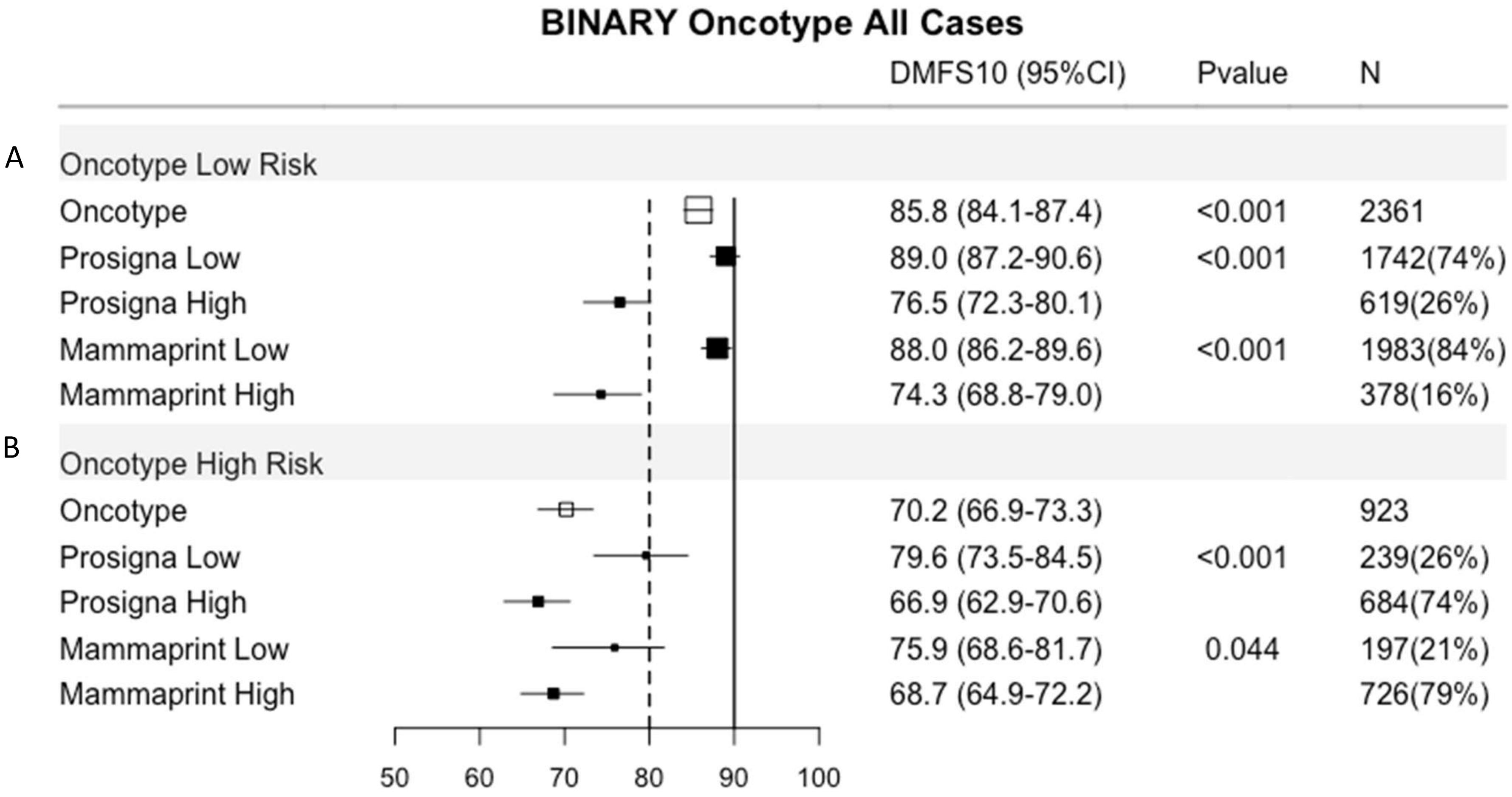

Supplementary Figure 3: Forest plot of DMFS10 by test and reclassification: Binary Oncotype first, ER+ve/HER2-ve cases – Node-ve, no chemotherapy

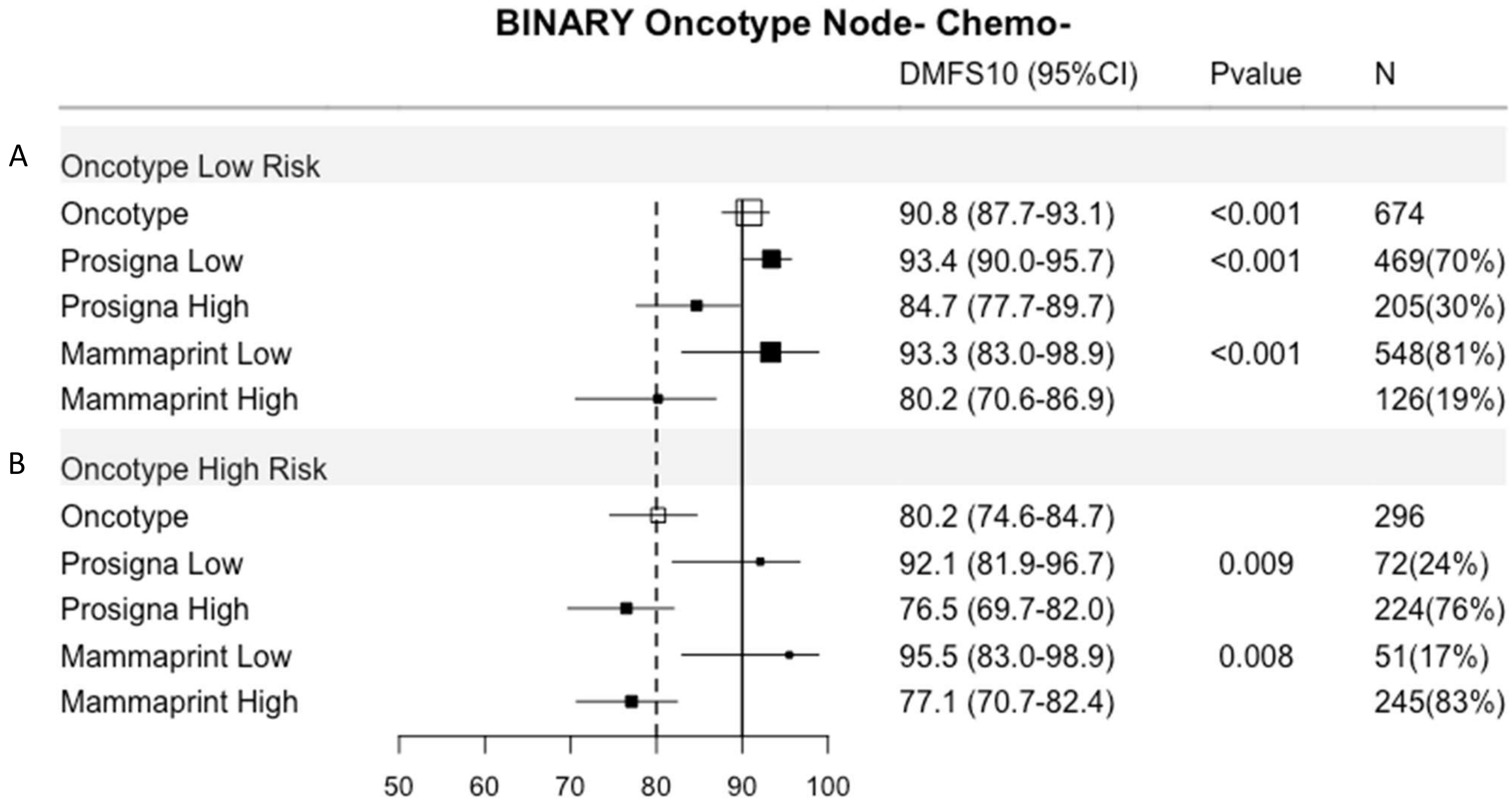

Supplementary Figure 4: Forest plot of DMFS10 by test and reclassification: Prosigna first, All ER+ve/HER2-ve cases

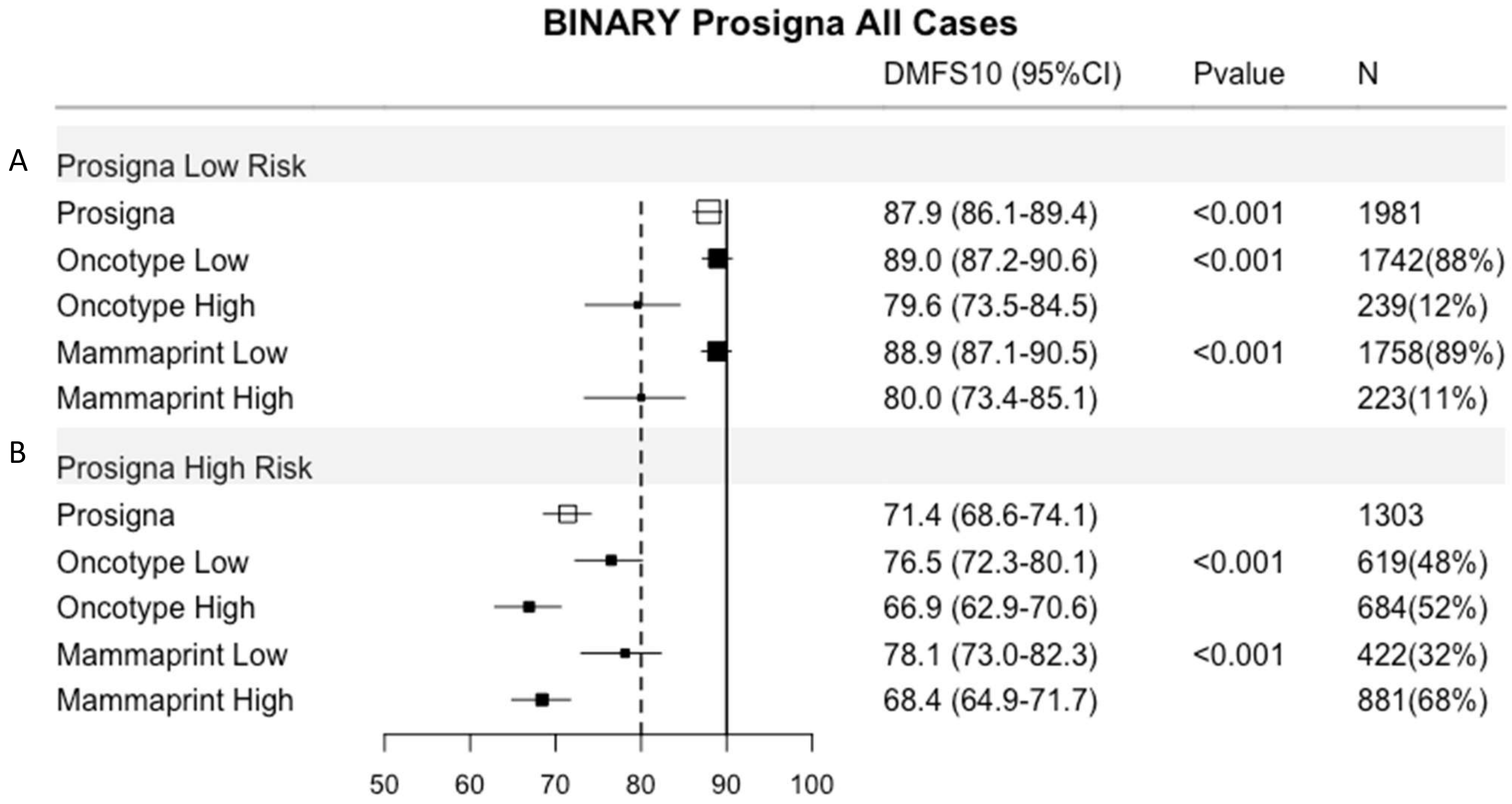

Supplementary Figure 5: Forest plot of DMFS10 by test and reclassification: Prosigna first, ER+ve/HER2-ve cases – Node-ve, no chemotherapy

BINARY Prosigna Node- Chemo-

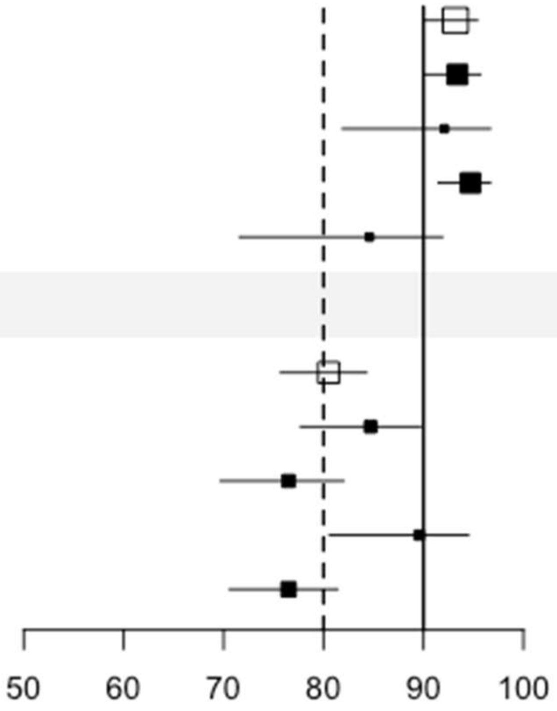

		DMFS10 (95%CI)	Pvalue	N
A	**Prosigna Low Risk**			
	Prosigna	93.2 (90.1-95.4)	<0.001	541
	Oncotype Low	93.4 (90.0-95.7)	0.419	469(87%)
	Oncotype High	92.1 (81.9-96.7)		72(13%)
	Mammaprint Low	94.7 (91.5-96.7)	<0.001	466(86%)
	Mammaprint High	84.6 (71.6-91.9)		75(14%)
B	**Prosigna High Risk**			
	Prosigna	80.5 (75.7-84.3)		429
	Oncotype Low	84.7 (77.7-89.7)	0.012	205(48%)
	Oncotype High	76.5 (69.7-82.0)		224(52%)
	Mammaprint Low	89.6 (80.6-94.5)	0.002	133(31%)
	Mammaprint High	76.5 (70.6-81.4)		296(69%)

Supplementary Figure 6: Forest plot of DMFS10 by test and reclassification: Mammaprint first, All ER+ve/HER2-ve cases

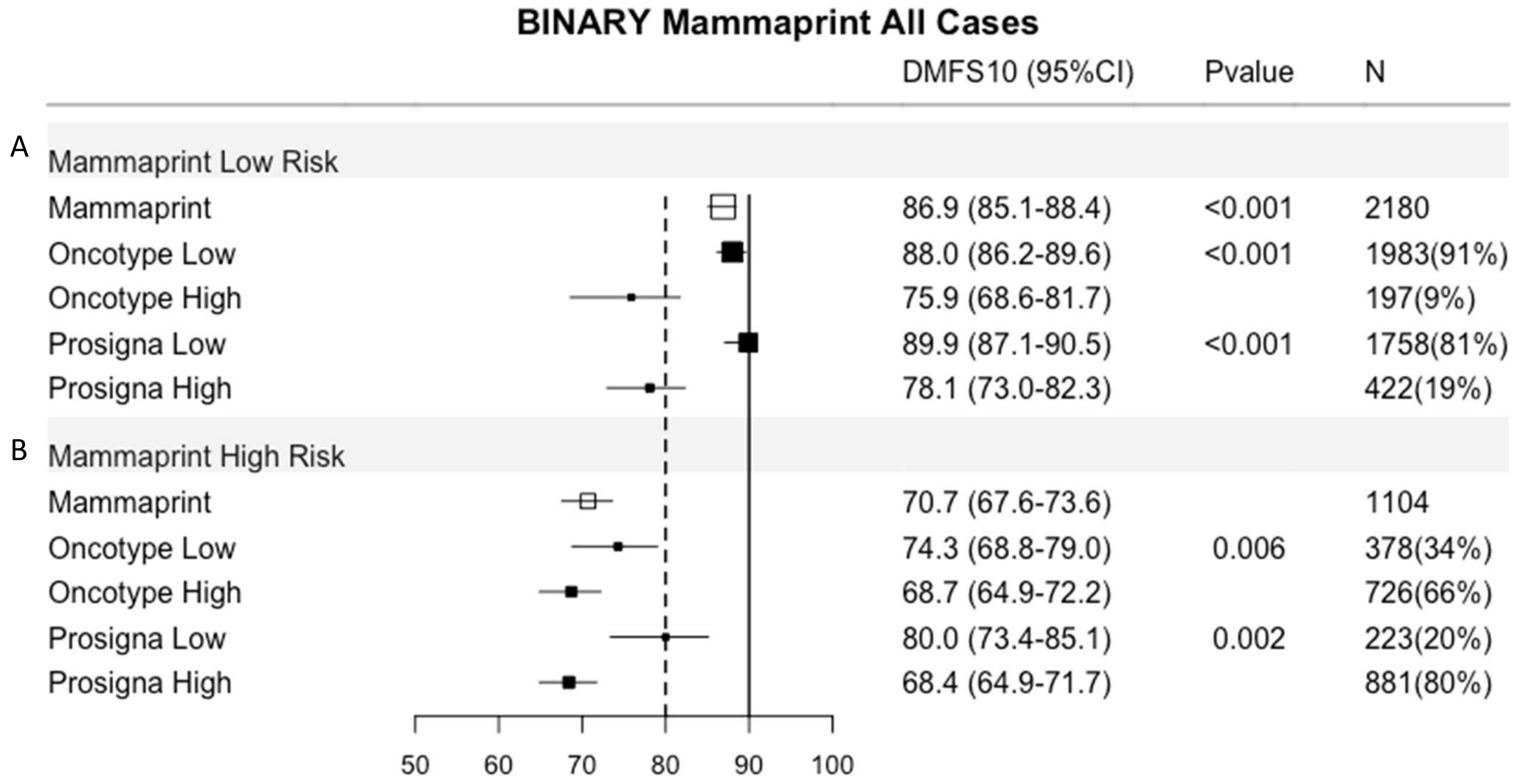

Supplementary Figure 7: Forest plot of DMFS10 by test and reclassification: Mammaprint first, ER+ve/HER2-ve cases – Node-ve, no chemotherapy

BINARY Mammaprint Node- Chemo-

	DMFS10 (95%CI)	Pvalue	N
A Mammaprint Low Risk			
Mammaprint	93.5 (90.5-95.6)	<0.001	599
Oncotype Low	93.3 (90.1-95.5)	0.799	548(91%)
Oncotype High	95.5 (83.0-98.9)		51(9%)
Prosigna Low	94.7 (91.5-96.7)	0.069	466(78%)
Prosigna High	89.6 (80.6-94.5)		133(22%)
B Mammaprint High Risk			
Mammaprint	78.2 (73.0-82.5)		371
Oncotype Low	80.2 (70.6-86.9)	0.248	126(34%)
Oncotype High	77.1 (70.7-82.4)		245(66%)
Prosigna Low	84.6 (71.6-91.9)	0.098	75(20%)
Prosigna High	76.5 (70.6-81.4)		296(80%)

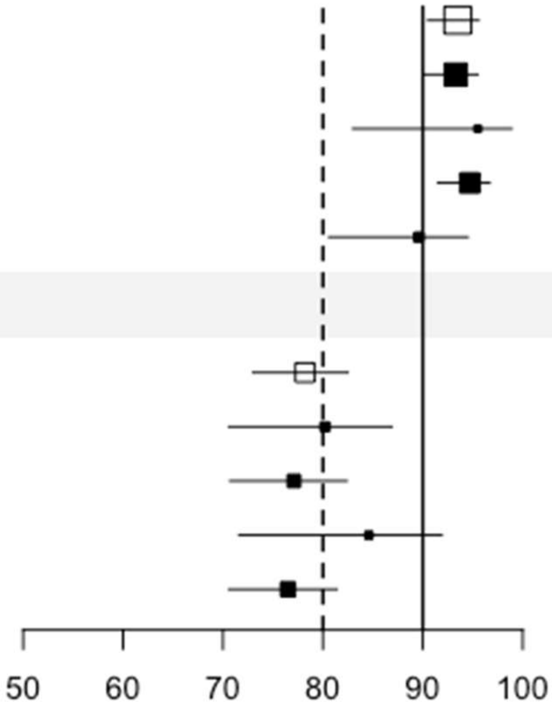

Supplementary Figure 8: Performance of “signature-trained” results in ER+ve/HER-ve - Node+ve, no chemotherapy

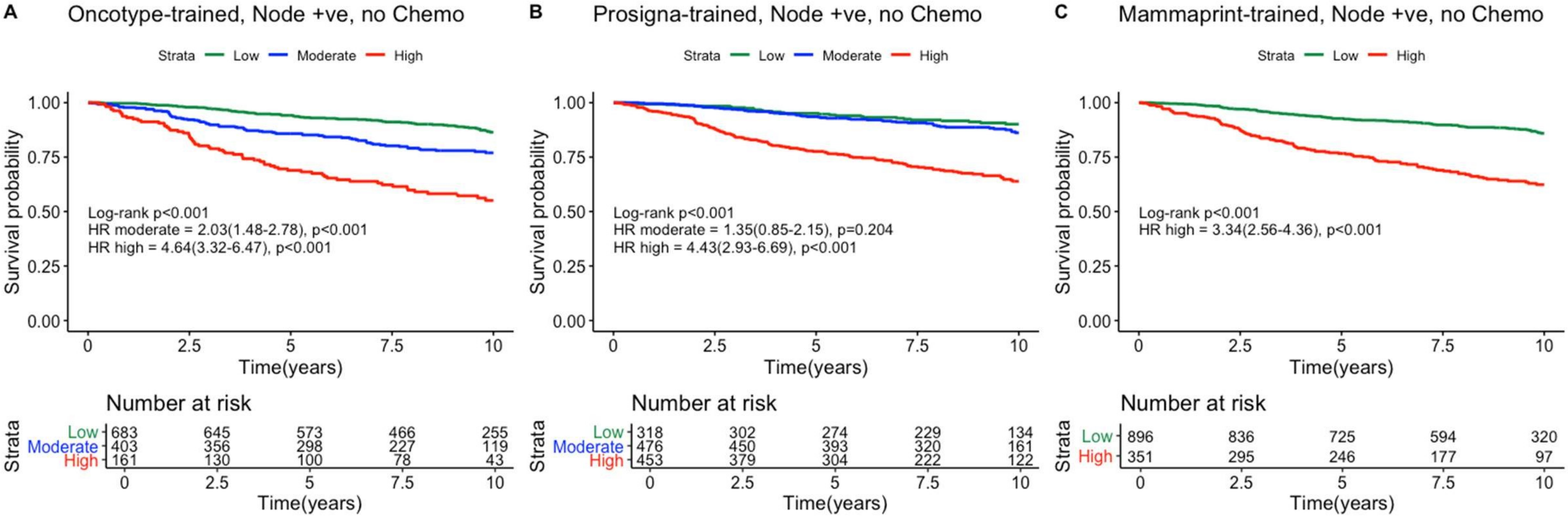

Supplementary Figure 9: Forest plot of DMFS by test and reclassification: Oncotype first, ER+ve/HER2-ve cases – Node+ve, no chemotherapy

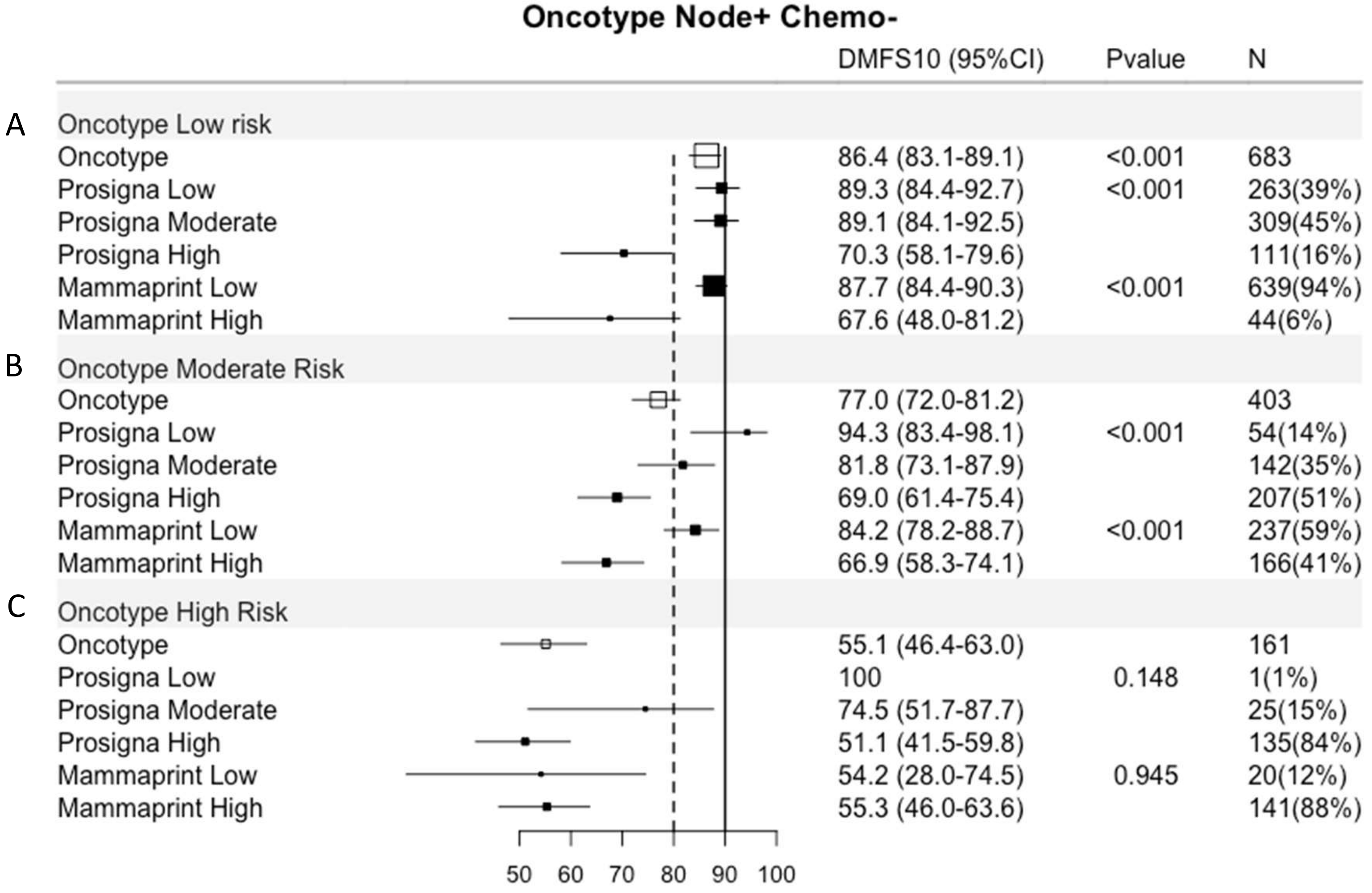

Supplementary Figure 10: Performance of “signature-trained” results in ER+ve/HER-ve – with chemotherapy

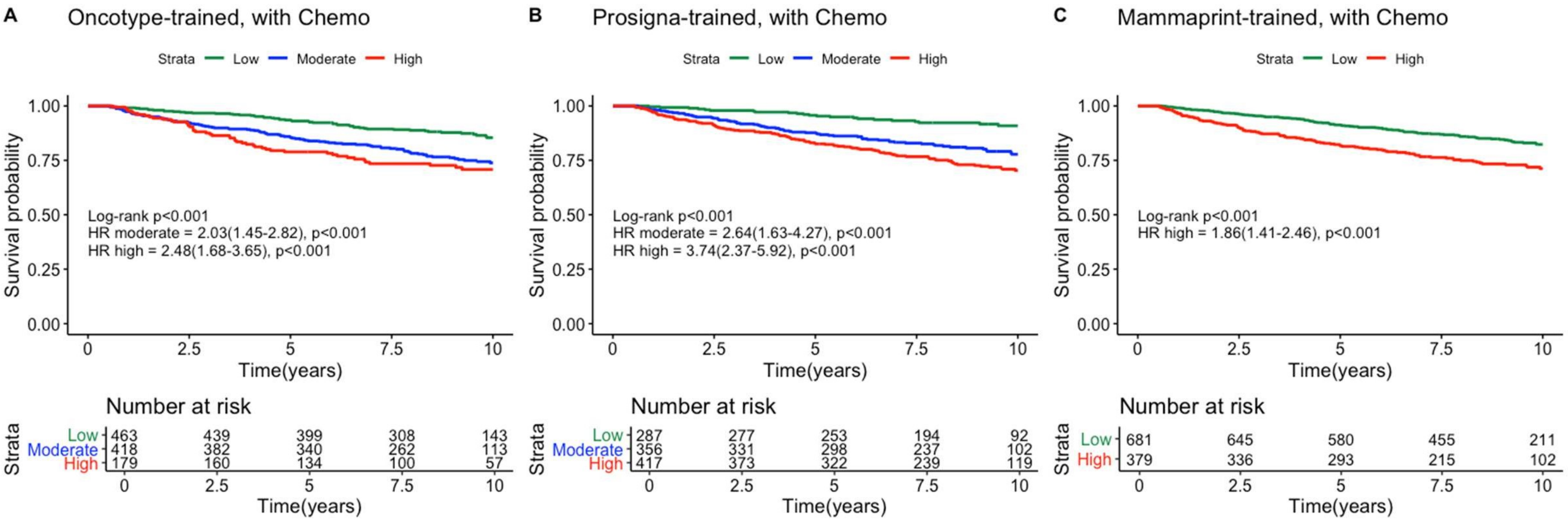

Supplementary Figure 11 Forest plot of DMFS by test and reclassification: Oncotype first, ER+ve/HER2-ve cases – with chemotherapy

Oncotyoe Chemo+

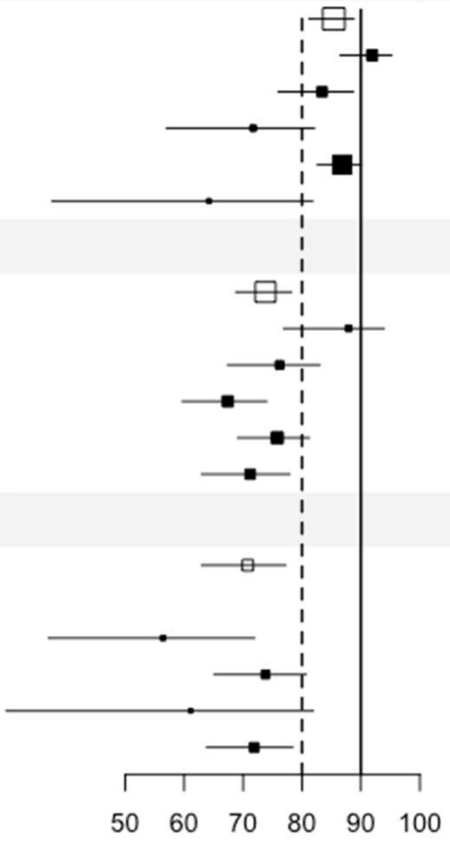

		DMFS10 (95%CI)	Pvalue	N
A	Oncotype Low risk			
	Oncotype	85.4 (81.2-88.8)	<0.001	463
	Prosigna Low	91.9 (86.5-95.2)	<0.001	205(44%)
	Prosigna Moderate	83.4 (76.0-88.7)		185(40%)
	Prosigna High	71.7 (57.0-82.1)		73(16%)
	Mammaprint Low	86.8 (82.6-90.0)	0.002	434(94%)
	Mammaprint High	64.2 (37.6-81.8)		29(6%)
B	Oncotype Moderate Risk			
	Oncotype	73.8 (68.8-78.2)		418
	Prosigna Low	87.9 (76.9-93.9)	0.007	77(18%)
	Prosigna Moderate	76.2 (67.4-83.0)		133(32%)
	Prosigna High	67.4 (59.7-74.0)		208(50%)
	Mammaprint Low	75.8 (69.1-81.2)	0.318	233(56%)
	Mammaprint High	71.2 (63.0-77.9)		185(44%)
C	Oncotype High Risk			
	Oncotype	70.8 (63.0-77.2)		179
	Prosigna Low	100	0.108	5(3%)
	Prosigna Moderate	56.4 (37.0-71.9)		38(21%)
	Prosigna High	73.8 (65.1-80.7)		136(76%)
	Mammaprint Low	61.1 (29.8-81.9)	0.537	14(8%)
	Mammaprint High	71.9 (63.8-78.4)		165(92%)

Supplementary Figure 12: Forest plot of DMFS by test and reclassification: Prosigna first, ER+ve/HER2-ve cases – Node+ve, no chemotherapy

Prosigna Node+ Chemo-

		DMFS10 (95%CI)	Pvalue	N
A	Prosigna Low risk			
	Prosigna	90.1 (85.8-93.2)	<0.001	318
	Oncotype Low	89.3 (84.4-92.7)	0.701	263(83%)
	Oncotype Moderate	94.3 (83.4-98.1)		54(17%)
	Oncotype High	100		1(0.3%)
	Mammaprint Low	90.0 (85.7-93.1)	0.601	315(99%)
	Mammaprint High	100		3(1%)
B	Prosigna Moderate Risk			
	Prosigna	86.2 (82.1-89.4)		476
	Oncotype Low	89.1 (84.1-92.5)	0.008	309(65%)
	Oncotype Moderate	81.8 (73.1-87.9)		142(30%)
	Oncotype High	74.5 (51.7-87.7)		25(5%)
	Mammaprint Low	86.6 (82.2-90.0)	0.371	415(87%)
	Mammaprint High	83.6 (70.7-91.1)		61(13%)
C	Prosigna High Risk			
	Prosigna	63.9 (58.6-68.7)		453
	Oncotype Low	70.3 (58.1-79.6)	<0.001	111(24%)
	Oncotype Moderate	69.0 (61.4-75.4)		207(46%)
	Oncotype High	51.1 (41.5-59.8)		135(30%)
	Mammaprint Low	75.7 (66.8-82.5)	<0.001	166(37%)
	Mammaprint High	57.4 (50.8-63.5)		287(63%)

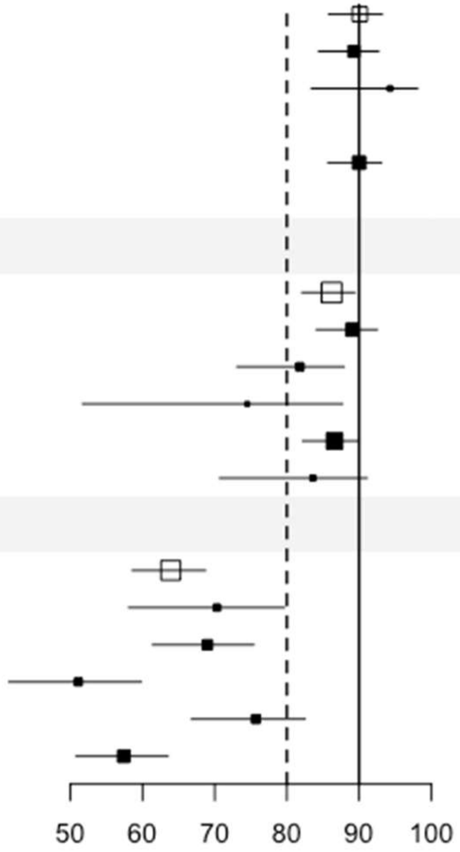

Supplementary Figure 13: Forest plot of DMFS by test and reclassification: Prosigna first, ER+ve/HER2-ve cases – with chemotherapy

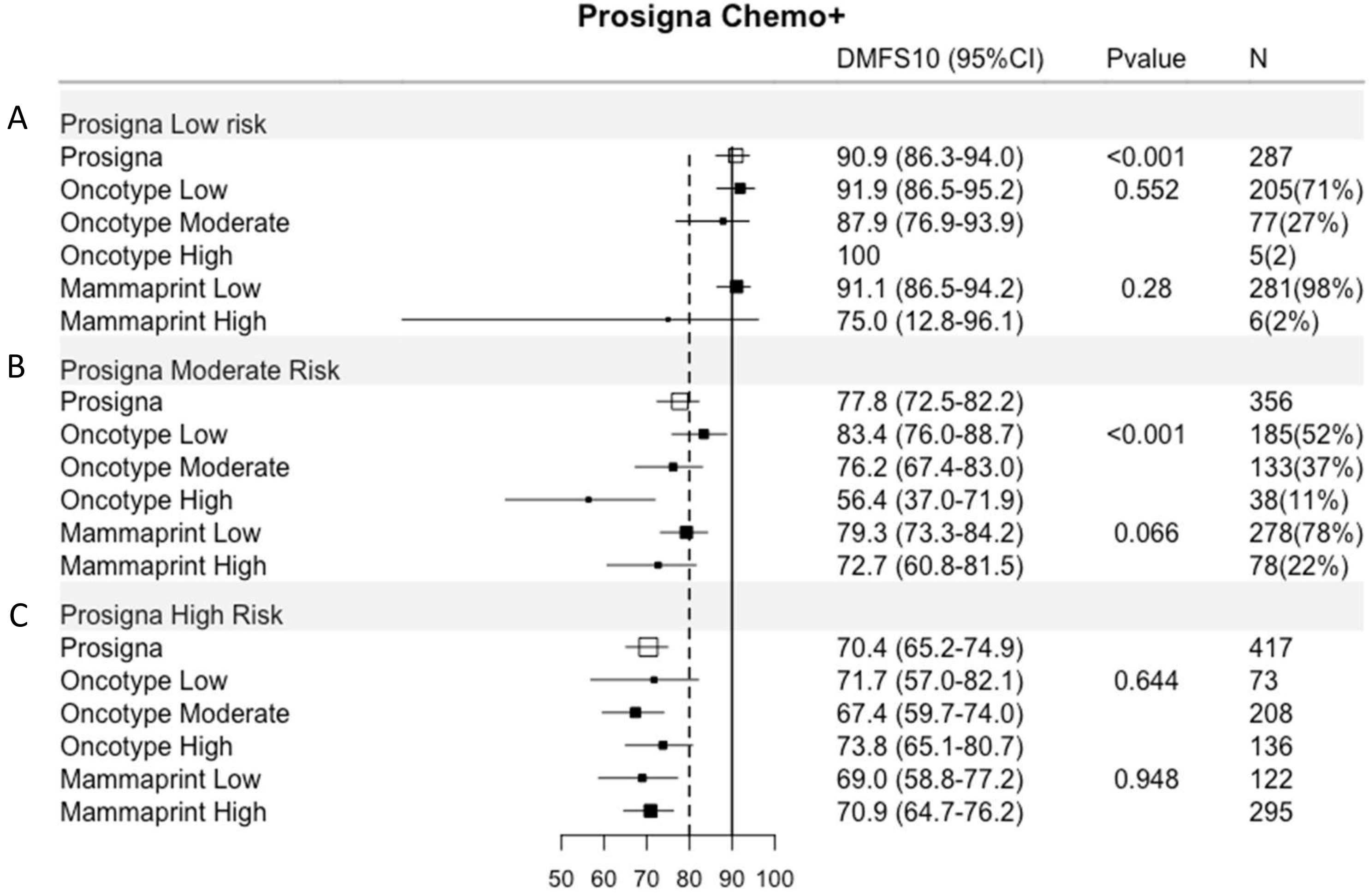

Supplementary Figure 14: Forest plot of DMFS by test and reclassification: Mammaprint first, ER+ve/HER2-ve cases – Node+ve, no chemotherapy

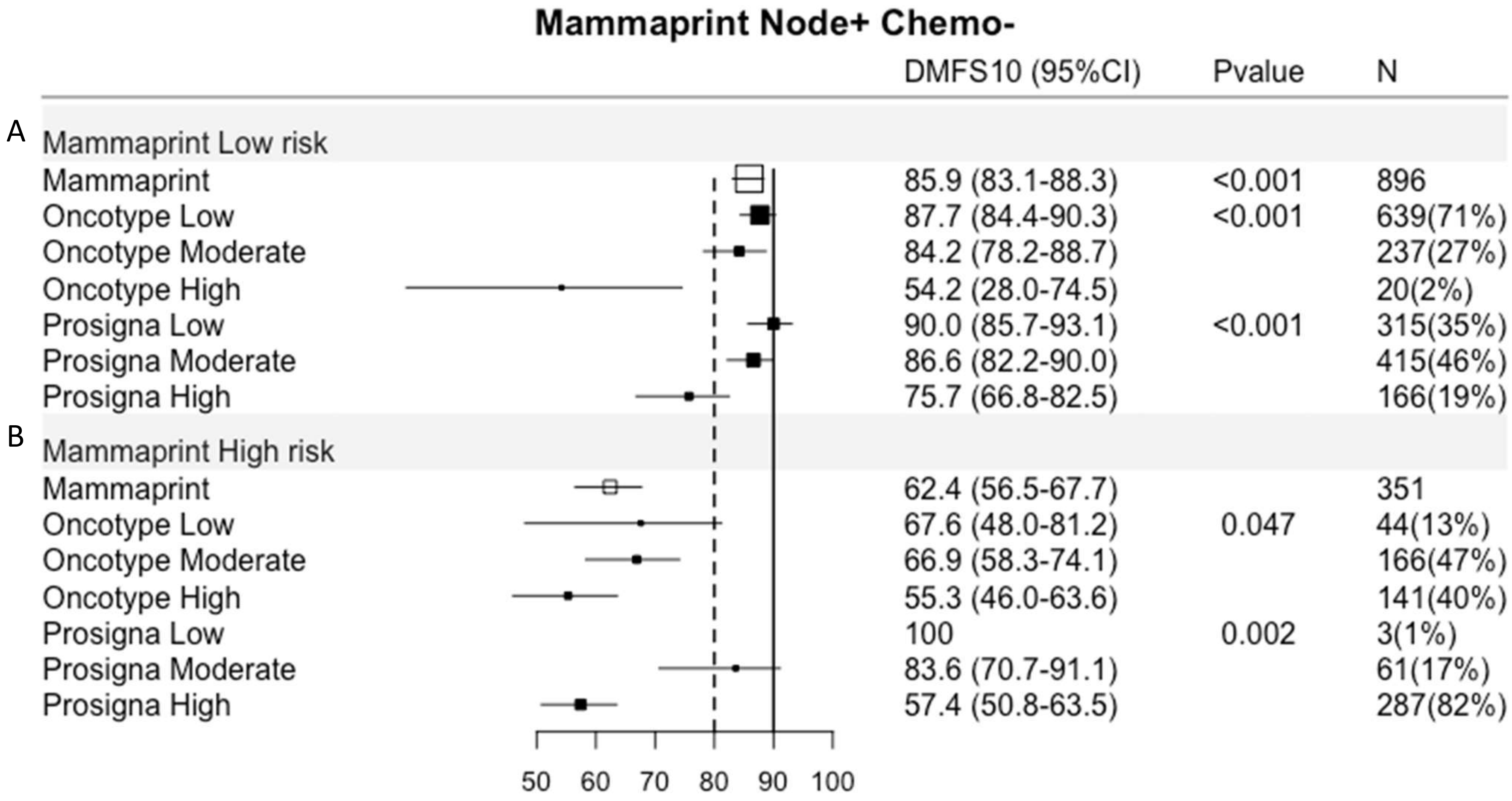

Supplementary Figure 15: Forest plot of DMFS by test and reclassification: Mammaprint first, ER+ve/HER2-ve cases – with chemotherapy

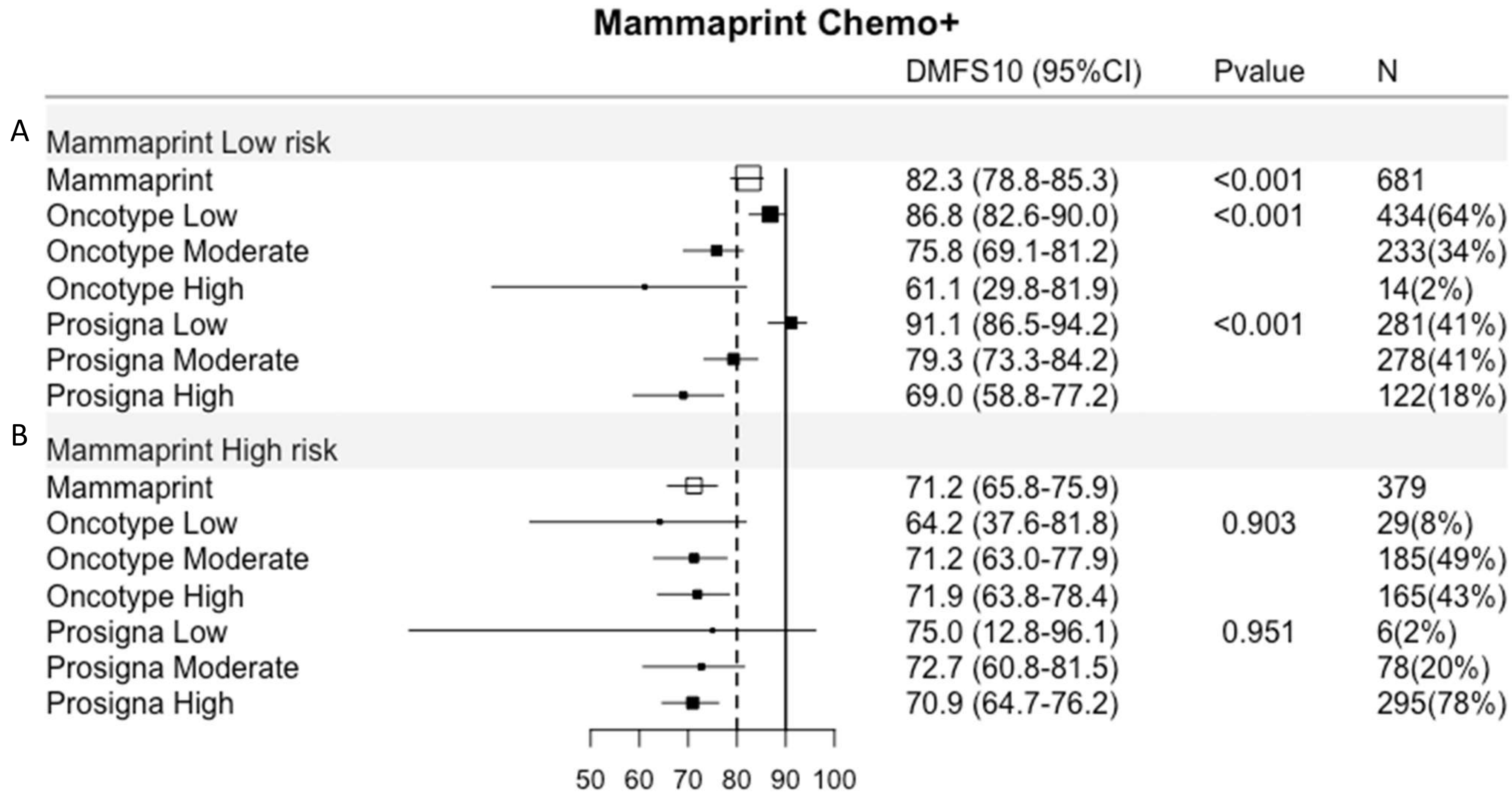

Supplementary Figure 16: Performance of "signature-trained" results in HR+ve/HER2+ve and HER2-ve patients in the TEAM trial cohort

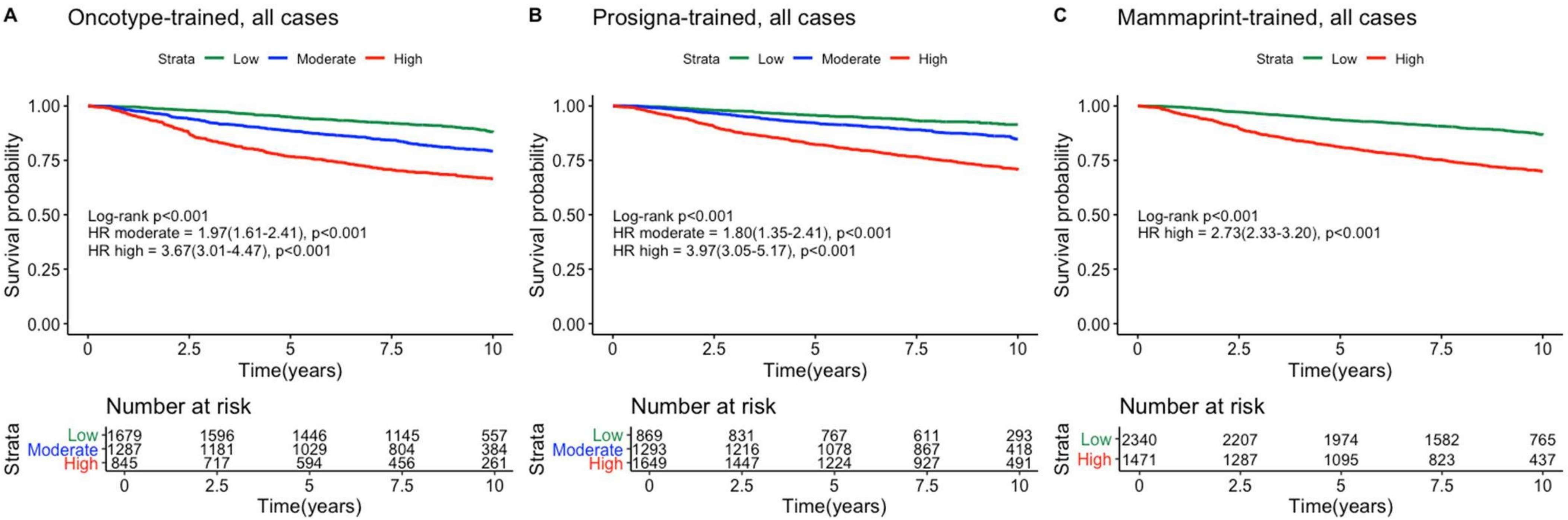

Supplementary Figure 17: Forest plot of Oncotype cases (HR+ve/HER2any) reclassified by Prosigna and Mammaprint

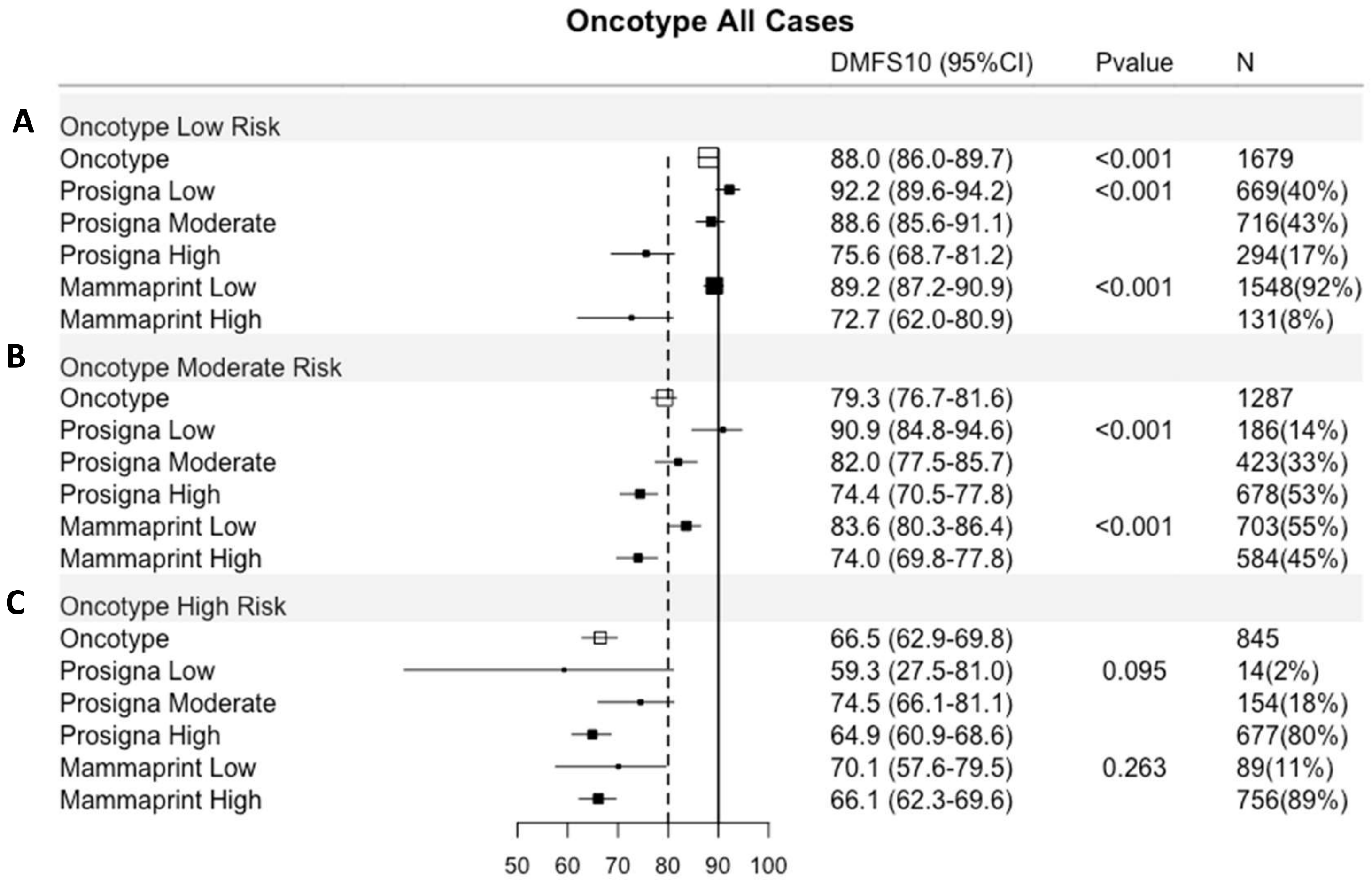

Supplementary Figure 18: Forest plot of Prosigna cases (HR+ve/HER2any) reclassified by Oncotype and Mammaprint

Prosigna All Cases

		DMFS10 (95%CI)	Pvalue	N
A	**Prosigna Low risk**			
	Prosigna	91.4 (89.1-93.3)	<0.001	869
	Oncotype Low	92.2 (89.6-94.2)	<0.001	669(77%)
	Oncotype Moderate	90.9 (84.8-94.6)		186(21%)
	Oncotype High	59.3 (27.5-81.0)		14(2%)
	Mammaprint Low	91.9 (89.6-93.7)	<0.001	852(98%)
	Mammaprint High	67.0 (37.7-84.9)		17(2%)
B	**Prosigna Moderate Risk**			
	Prosigna	84.8 (82.4-86.9)		1293
	Oncotype Low	88.6 (85.6-91.1)	<0.001	716(55%)
	Oncotype Moderate	82.0 (77.5-85.7)		423(33%)
	Oncotype High	74.5 (66.1-81.1)		154(12%)
	Mammaprint Low	86.4 (83.8-88.7)	<0.001	1017(79%)
	Mammaprint High	78.7 (72.8-83.4)		276(21%)
C	**Prosigna High Risk**			
	Prosigna	70.8 (68.2-73.1)		1649
	Oncotype Low	75.6 (68.7-81.2)	<0.001	294(18%)
	Oncotype Moderate	74.4 (70.5-77.8)		678(41%)
	Oncotype High	64.9 (60.9-68.6)		677(41%)
	Mammaprint Low	78.0 (73.2-82.0)	<0.001	471(29%)
	Mammaprint High	68.0 (64.9-70.8)		1178(71%)

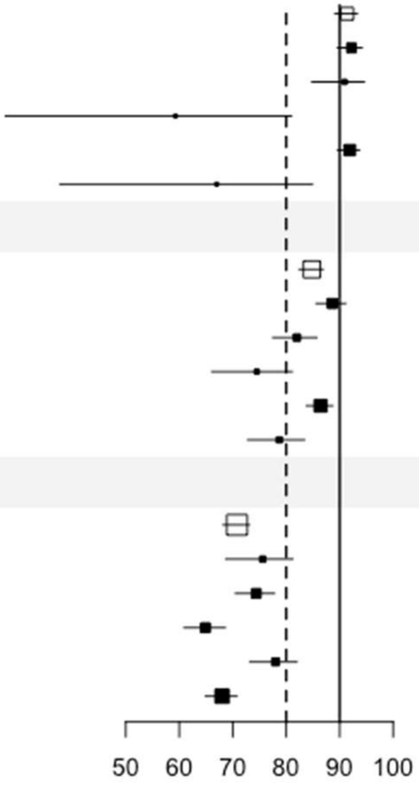

Supplementary Figure 19: Forest plot of Mammaprint cases (HR+ve/HER2any) reclassified by Oncotype and Prosigna

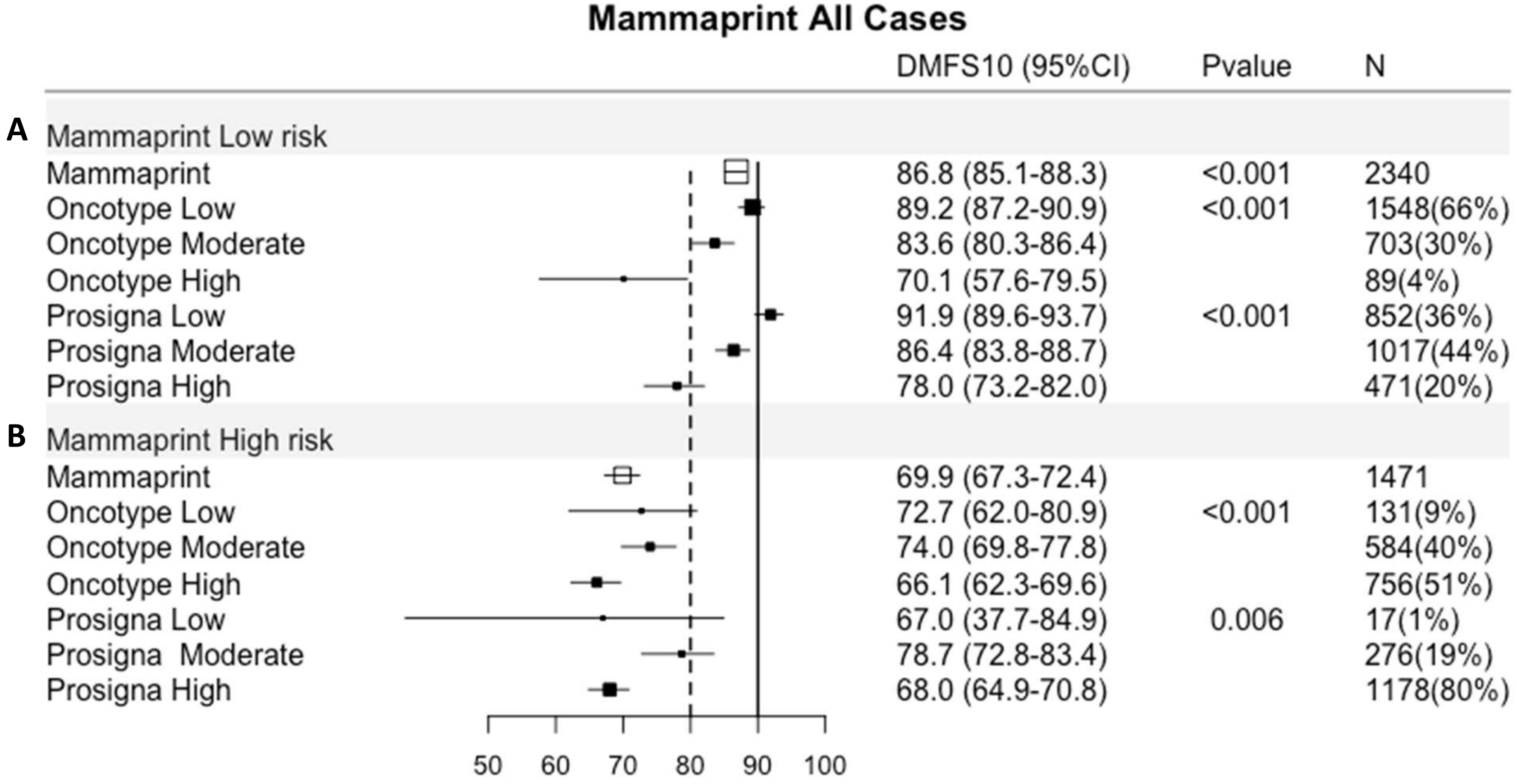

Supplementary Figure 20:Performance of “signature-trained” results in HR+ve/HER2+ve and HER2-ve node negative patients treated without chemotherapy in the TEAM trial cohort

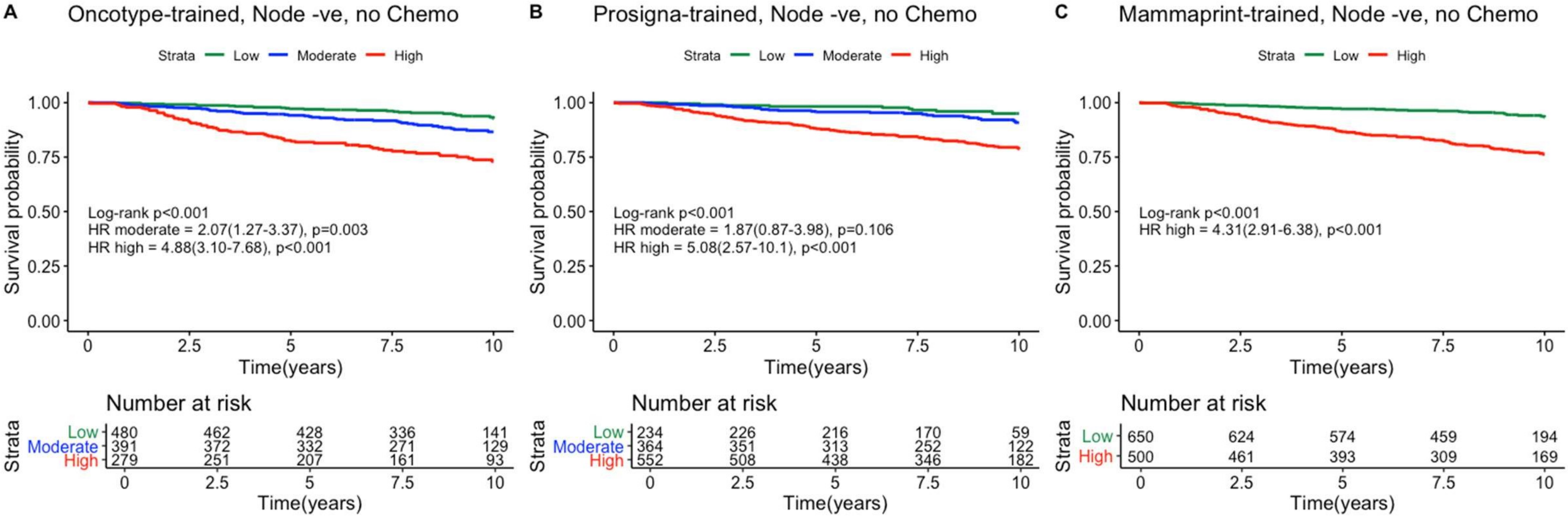

Supplementary Figure 21: Forest plot of Oncotype cases (HR+ve/HER2any) node-ve no chemotherapy reclassified by Prosigna and Mammaprint

Oncotype Node- Chemo-

		DMFS10 (95%CI)	Pvalue	N
A	Oncotype Low risk			
	Oncotype	92.6 (89.1-95.1)	<0.001	480
	Prosigna Low	96.7 (92.2-98.6)	0.023	182(38%)
	Prosigna Moderate	92.7 (86.9-96.0)		199(41%)
	Prosigna High	84.6 (71.6-92.0)		99(21%)
	Mammaprint Low	93.8 (90.2-96.2)	0.006	425(89%)
	Mammaprint High	82.6 (66.0-91.6)		55(11%)
B	Oncotype Moderate Risk			
	Oncotype	86.6 (82.2-90.0)		391
	Prosigna Low	94.1 (65.0-99.1)	0.098	46(12%)
	Prosigna Moderate	87.2 (78.0-92.8)		121(31%)
	Prosigna High	84.4 (78.3-88.9)		224(57%)
	Mammaprint Low	92.0 (86.1-95.5)	0.003	197(50%)
	Mammaprint High	81.4 (74.4-86.6)		194(50%)
C	Oncotype High Risk			
	Oncotype	73.0 (66.7-78.3)		279
	Prosigna Low	50.0 (11.1-80.4)	0.014	6(2%)
	Prosigna Moderate	92.3 (77.5-97.5)		44(16%)
	Prosigna High	70.2 (63.1-76.2)		229(82%)
	Mammaprint Low	92.7 (73.9-98.1)	0.043	28(10%)
	Mammaprint High	70.9 (64.2-76.6)		251(90%)

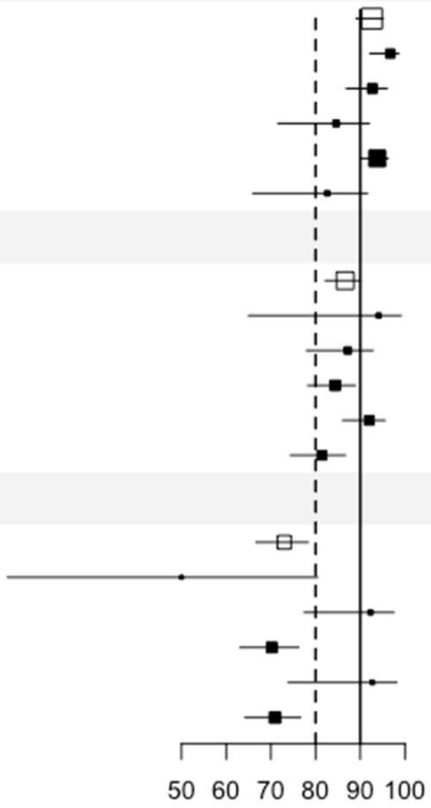

Supplementary Figure 22:Performance of "signature-trained" results in HR+ve/HER2+ve and HER2-ve node positive patients treated without chemotherapy in the TEAM trial cohort

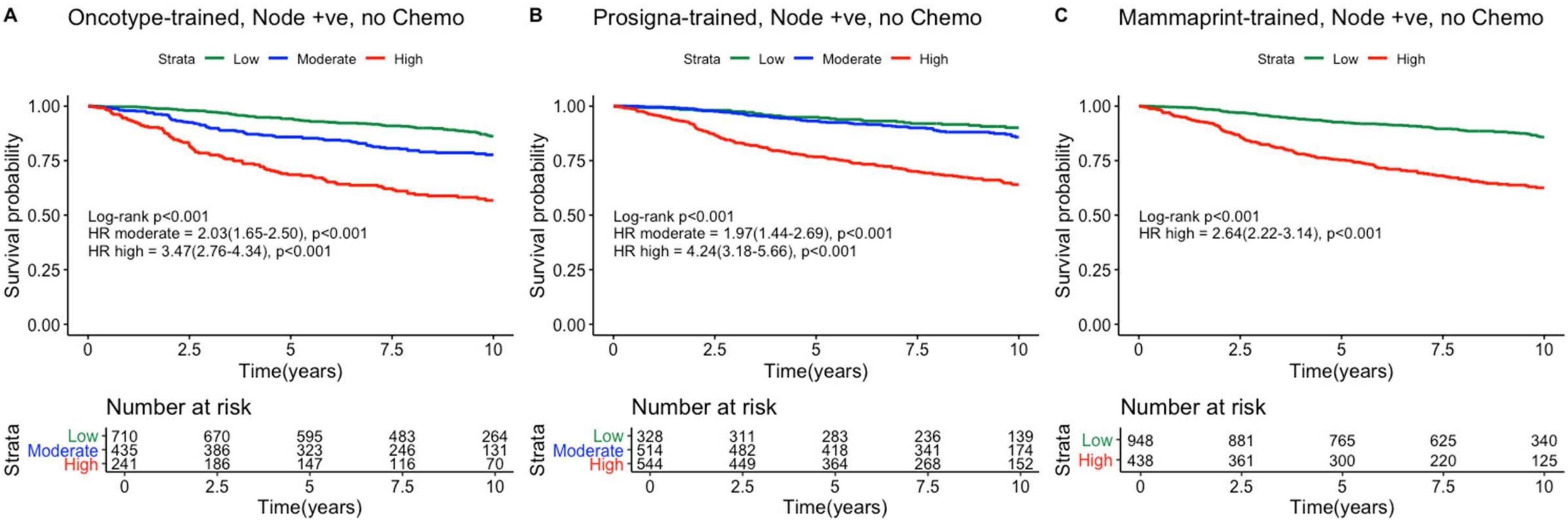

Supplementary Figure 23: Forest plot of Oncotype cases (HR+ve/HER2any) node+ve no chemotherapy reclassified by Prosigna and Mammaprint

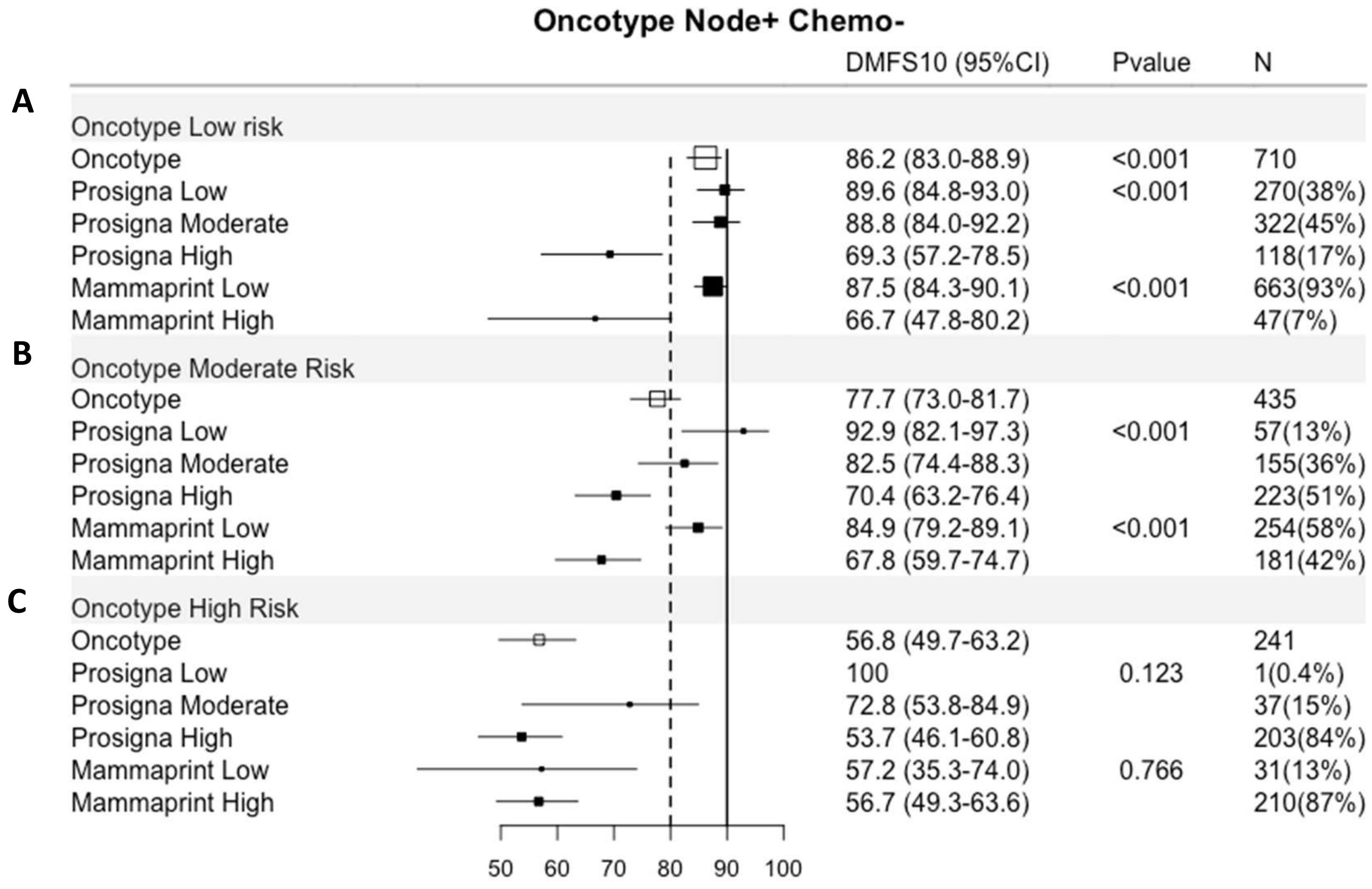

Supplementary Figure 24:Performance of "signature-trained" results in HR+ve/HER2+ve and HER2-ve any nodal status patients treated with chemotherapy in the TEAM trial cohort

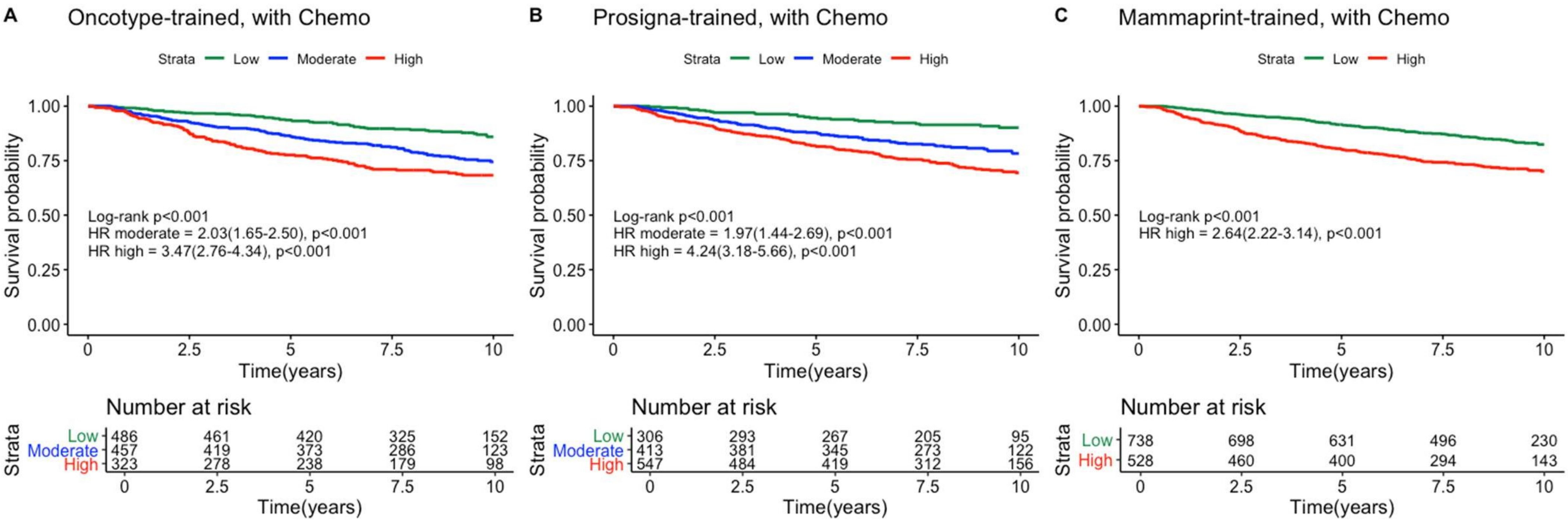

Supplementary Figure 25: Forest plot of Oncotype cases (HR+ve/HER2any) node any treated with chemotherapy reclassified by Prosigna and Mammaprint

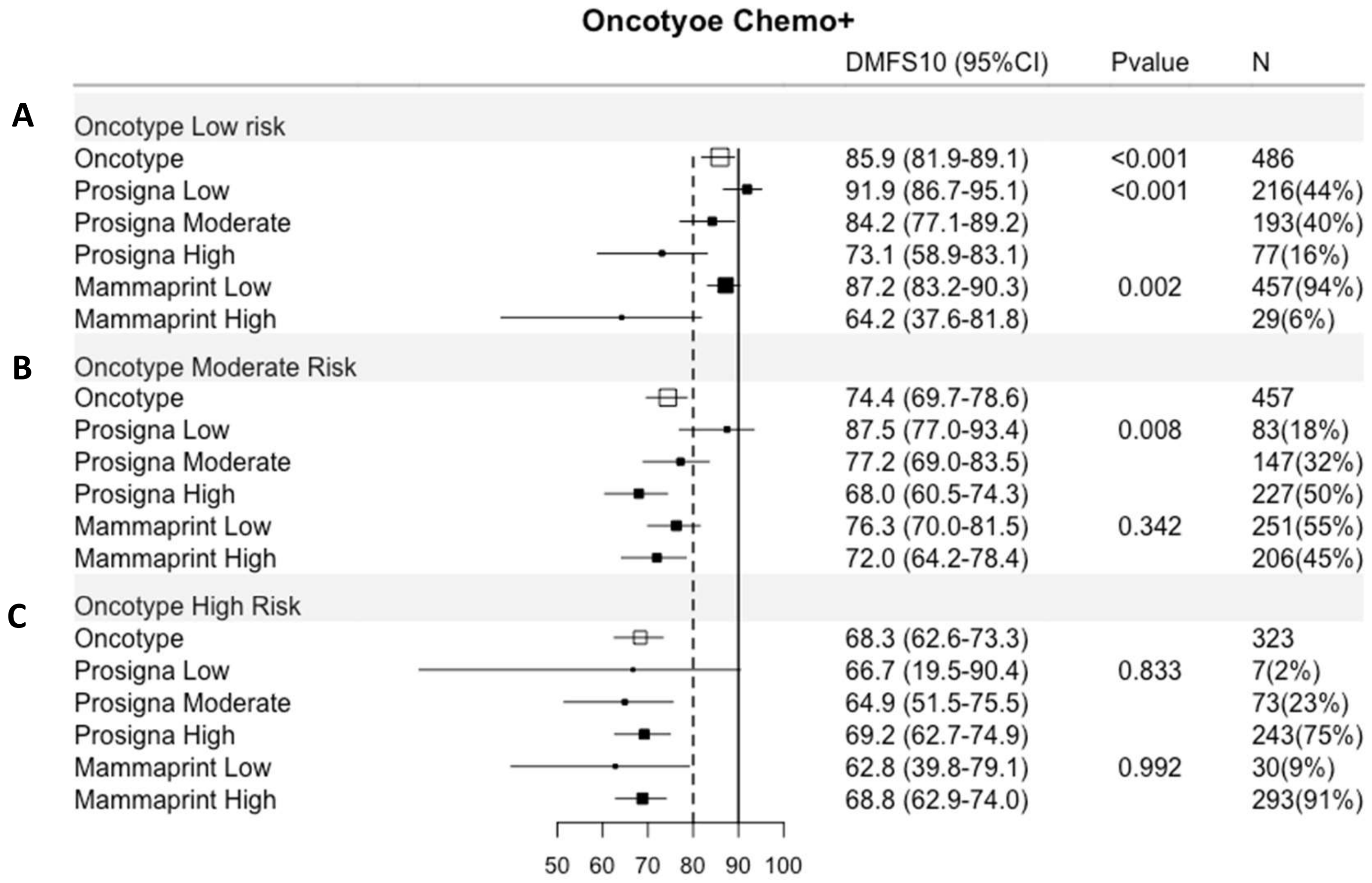

Supplementary Figure 26: Forest plot of Prosigna cases (HR+ve/HER2any) node-ve no chemotherapy reclassified by Oncotype and Mammaprint

Prosigna Node- Chemo-

		DMFS10 (95%CI)	Pvalue	N
A	Prosigna Low risk			
	Prosigna	95.0 (90.4-97.4)	<0.001	234
	Oncotype Low	96.7 (92.2-98.6)	<0.001	182(78%)
	Oncotype Moderate	94.1 (65.0-99.1)		46(20%)
	Oncotype High	21.5 (5.13-90.3)		6(2%)
	Mammaprint Low	95.9 (91.3-98.1)	<0.001	228(97%)
	Mammaprint High	66.7 (19.5-90.4)		6(3%)
B	Prosigna Moderate Risk			
	Prosigna	90.9 (86.7-93.8)		364
	Oncotype Low	92.7 (86.9-96.0)	0.324	199(55%)
	Oncotype Moderate	87.2 (78.0-92.8)		121(33%)
	Oncotype High	92.3 (77.5-97.5)		44(12%)
	Mammaprint Low	93.2 (88.5-96.0)	0.015	269(74%)
	Mammaprint High	84.3 (73.5-91.0)		95(26%)
C	Prosigna High Risk			
	Prosigna	78.6 (74.5-82.2)		552
	Oncotype Low	84.6 (71.6-92.0)	<0.001	99(18%)
	Oncotype Moderate	84.4 (78.3-88.9)		224(41%)
	Oncotype High	70.2 (63.1-76.2)		229(41%)
	Mammaprint Low	89.6 (81.7-94.2)	<0.001	153(28%)
	Mammaprint High	74.5 (69.4-78.9)		399(72%)

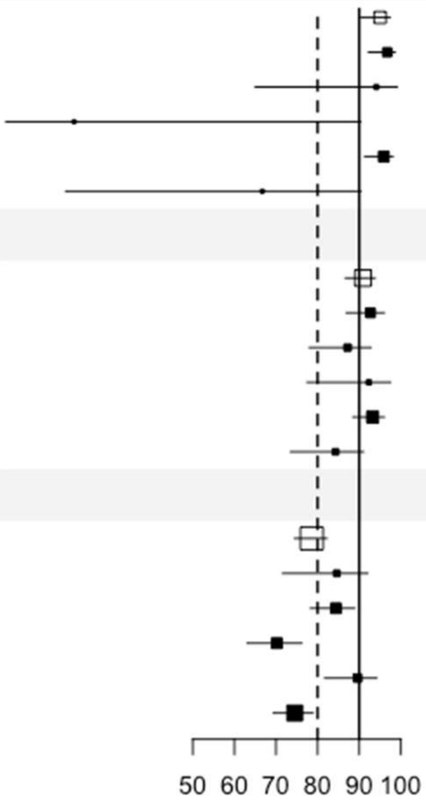

Supplementary Figure 27: Forest plot of Prosigna cases (HR+ve/HER2any) node+ve no chemotherapy reclassified by Oncotype and Mammaprint

Prosigna Node+ Chemo-

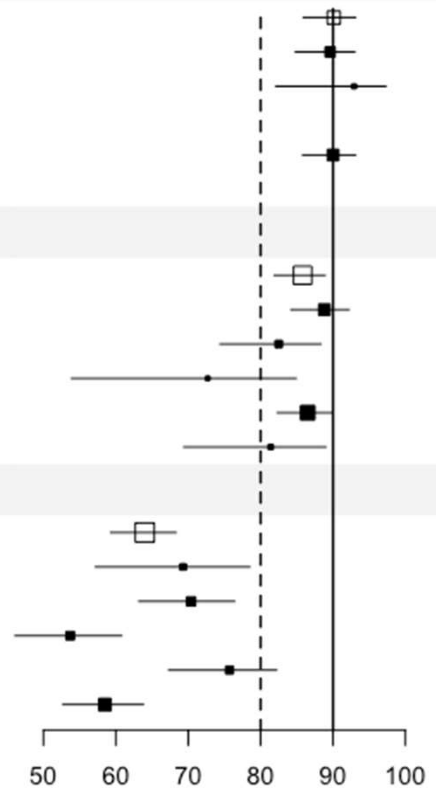

		DMFS10 (95%CI)	Pvalue	N
A	Prosigna Low risk			
	Prosigna	90.1 (85.9-93.1)	<0.001	328
	Oncotype Low	89.6 (84.8-93.0)	0.886	270(82%)
	Oncotype Moderate	92.9 (82.1-97.3)		57(17%)
	Oncotype High	100		1(0.3%)
	Mammaprint Low	90.0 (85.8-93.1)	0.6	325(99%)
	Mammaprint High	100		3(1%)
B	Prosigna Moderate Risk			
	Prosigna	85.8 (81.9-88.9)		514
	Oncotype Low	88.8 (84.2-92.2)	0.003	322(63%)
	Oncotype Moderate	82.5 (74.4-88.3)		155(30%)
	Oncotype High	72.7 (53.9-84.9)		37(7%)
	Mammaprint Low	86.5 (82.3-89.8)	0.137	441(86%)
	Mammaprint High	81.4 (69.4-89.0)		73(14%)
C	Prosigna High Risk			
	Prosigna	64.0 (59.3-68.3)		544
	Oncotype Low	69.3 (57.2-78.5)	<0.001	118(22%)
	Oncotype Moderate	70.4 (63.2-76.4)		223(41%)
	Oncotype High	53.7 (46.1-60.8)		203(37%)
	Mammaprint Low	75.7 (67.3-82.2)	<0.001	182(33%)
	Mammaprint High	58.5 (52.7-63.8)		362(67%)

Supplementary Figure 28: Forest plot of Prosigna cases (HR+ve/HER2any) node any and treated with chemotherapy reclassified by Oncotype and Mammaprint

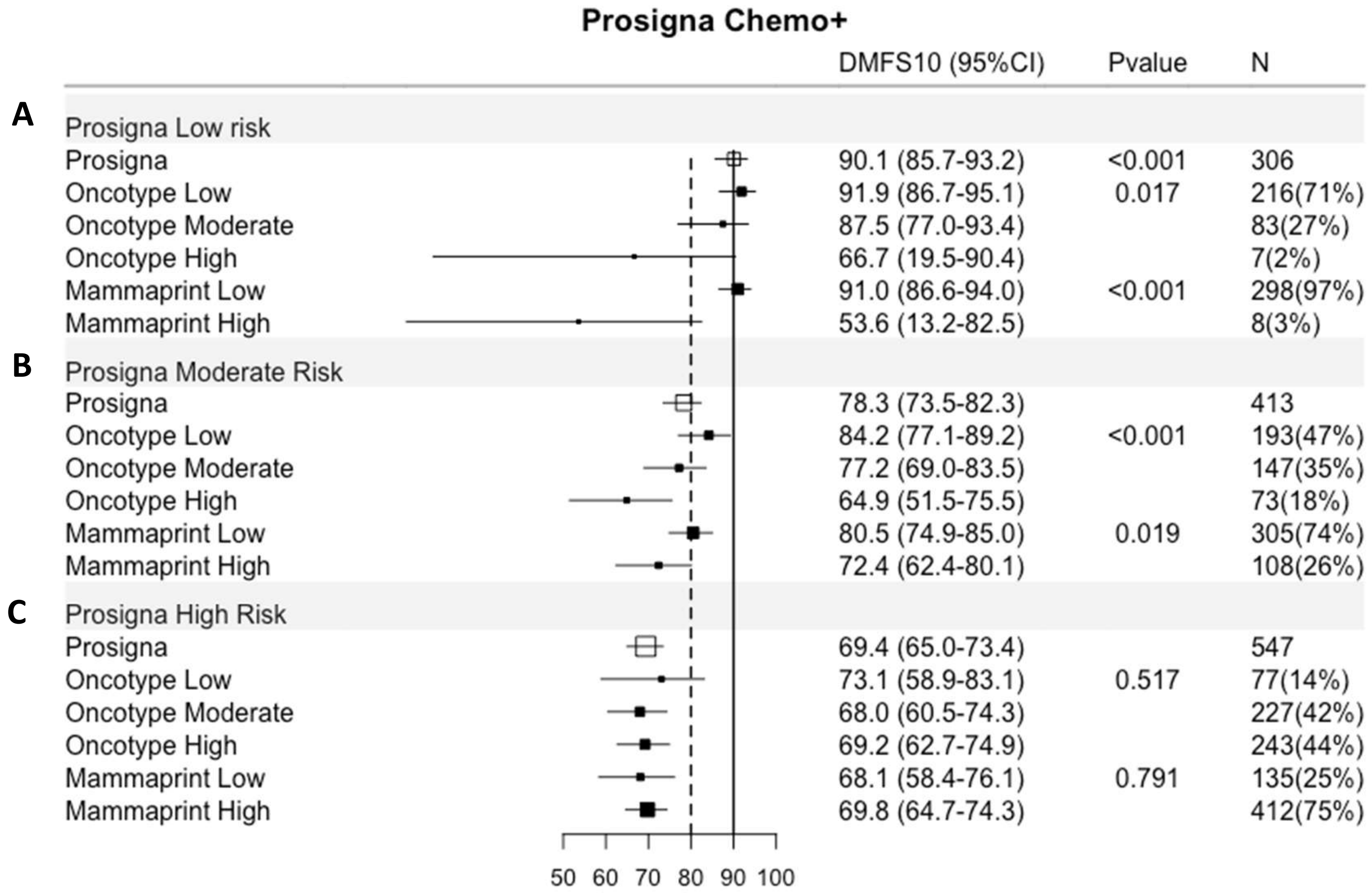

Supplementary Figure 29: Forest plot of Mammaprint cases (HR+ve/HER2any) node-ve no chemotherapy reclassified by Oncotype and Prosigna

Mammaprint Node- Chemo-

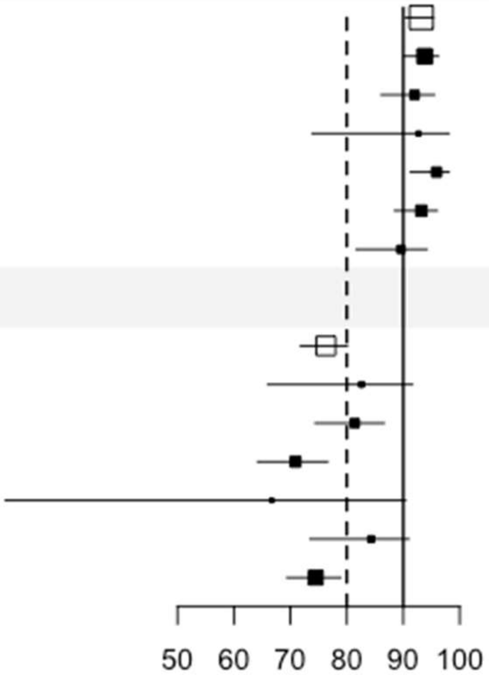

		DMFS10 (95%CI)	Pvalue	N
A	Mammaprint Low risk			
	Mammaprint	93.2 (90.4-95.3)	<0.001	650
	Oncotype Low	93.8 (90.2-96.2)	0.562	425(66%)
	Oncotype Moderate	92.0 (86.1-95.5)		197(30%)
	Oncotype High	92.7 (73.9-98.1)		28(4%)
	Prosigna Low	95.9 (91.3-98.1)	0.112	228(35%)
	Prosigna Moderate	93.2 (88.5-96.0)		269(41%)
	Prosigna High	89.6 (81.7-94.2)		153(24%)
B	Mammaprint High risk			
	Mammaprint	76.3 (71.8-80.1)		500
	Oncotype Low	82.6 (66.0-91.6)	0.01	55(11%)
	Oncotype Moderate	81.4 (74.4-86.6)		194(39%)
	Oncotype High	70.9 (64.2-76.6)		251(50%)
	Prosigna Low	66.7 (19.5-90.4)	0.103	6(1%)
	Prosigna Moderate	84.3 (73.5-91.0)		95(19%)
	Prosigna High	74.5 (69.4-78.9)		399(80%)

Supplementary Figure 30: Forest plot of Mammaprint cases (HR+ve/HER2any) node+ve no chemotherapy reclassified by Oncotype and Prosigna

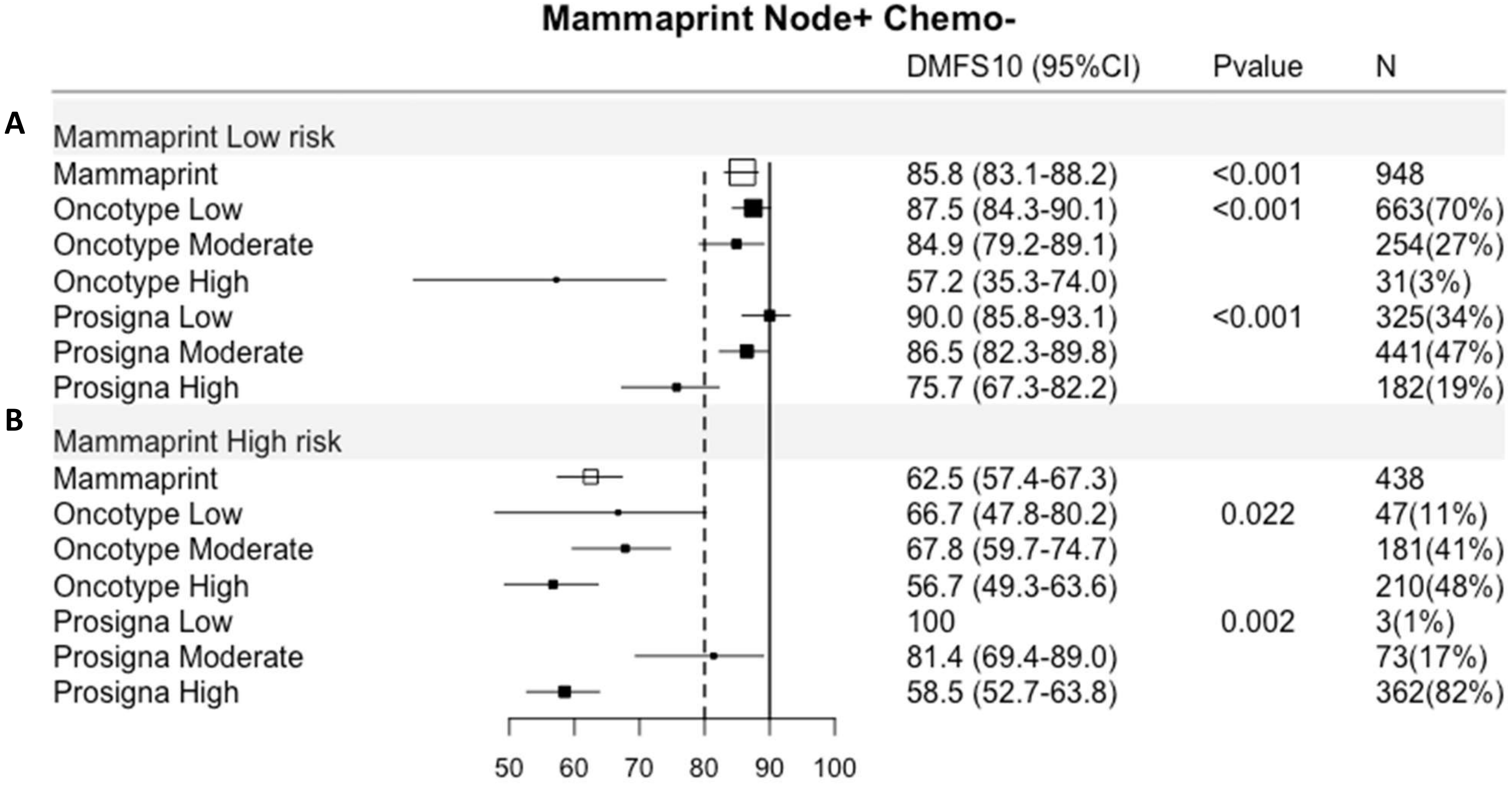

Supplementary Figure 31: Forest plot of Mammaprint cases (HR+ve/HER2any) node any treated with chemotherapy reclassified by Oncotype and Prosigna

Mammaprint Chemo+

		DMFS10 (95%CI)	Pvalue	N
A	Mammaprint Low risk			
	Mammaprint	82.4 (79.1-85.3)	<0.001	738
	Oncotype Low	87.2 (83.2-90.3)	<0.001	457(62%)
	Oncotype Moderate	76.3 (70.0-81.5)		251(34%)
	Oncotype High	62.8 (39.8-79.1)		30(4%)
	Prosigna Low	91.0 (85.8-93.1)	<0.001	298(41%)
	Prosigna Moderate	80.5 (74.9-85.0)		305(41%)
	Prosigna High	68.1 (58.4-76.1)		135(18%)
B	Mammaprint High risk			
	Mammaprint	70.0 (65.6-74.1)		528
	Oncotype Low	64.2 (37.6-81.8)	0.391	29(5%)
	Oncotype Moderate	72.0 (64.2-78.4)		206(39%)
	Oncotype High	68.8 (62.9-74.0)		293(56%)
	Prosigna Low	53.6 (13.2-82.5)	0.65	8(2%)
	Prosigna Moderate	72.4 (62.4-80.1)		108(20%)
	Prosigna High	69.8 (64.7-74.3)		412(78%)

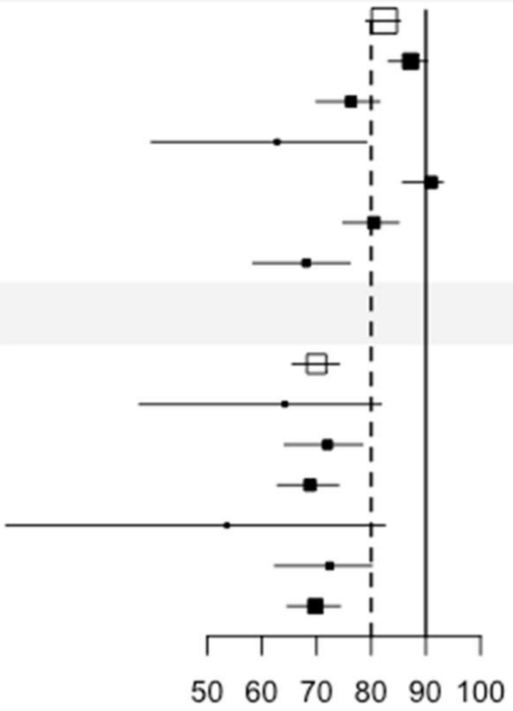

Supplement: Supplementary file 1 — Supplementary Information [file 41523_2021_297_MOESM1_ESM.pdf]
